# Improvement of precise large-fragment deletion with a versatile nuclease prime editor

**DOI:** 10.1038/s41421-026-00923-3

**Published:** 2026-08-14

**Authors:** Xiangyang Li, Wenjun Sun, Gefei Wang, Xinpeng Ma, Mengyao Dai, Yang Zhao, Liqun Lei, Xuechao Cai, Shuaifeng Li, Shisheng Huang, Xingxu Huang, Xia Lin, Tingbo Liang

**Affiliations:** 1 The Key Laboratory of Pancreatic Diseases of Zhejiang Province, The First Affiliated Hospital, Zhejiang University School of Medicine, Hangzhou, China; 2 School of Life Science and Technology, ShanghaiTech University, Pudong New Area, Shanghai, China; 3 Department of Hepatobiliary and Pancreatic Surgery, The First Affiliated Hospital, Zhejiang University School of Medicine, Hangzhou, China

**Keywords:** Biological techniques, DNA damage and repair

Dear Editor,

Among 241,074 known pathogenic or likely pathogenic human genetic variants, ~27.7% were deletions or replacements (i.e., insertions following deletions)^1^. Many abnormal deletions and replacements involved relatively large DNA fragments, including ~953 variants with deletions exceeding 1 kb (Supplementary Fig. S1a), posing a major challenge for genome editing. The prime editing (PE) system represented a significant breakthrough for diverse genome editing, while it was limited for deleting larger DNA fragments^2,3^. The recently reported nuclease-based prime editor (PEn) combined with paired pegRNAs provided a novel prime editing tool for large-fragment deletions and simultaneous insertions^4,5^. However, the current platforms based on PEn still exhibited overall suboptimal efficiencies and produced unwanted edits, significantly hindering the applicability of this versatile tool.

i53 inhibits NHEJ by blocking 53BP1 recruitment^6^, enabling the development of our uPEn platform for improved PEn-mediated precise editing^7^. We therefore explored whether the NHEJ-inhibition strategy could enhance large-fragment deletion outcomes. In this work, we developed a novel strategy by combining uPEn protein with paired pegRNAs (Fig. 1a). Here, we designated the system as ‘uDAR’ (uPEn-mediated large-fragment deletion and simultaneous replacement), which showed significant improvements in purity and efficiency over PEn-mediated large-fragment edits.

**Fig. 1:**
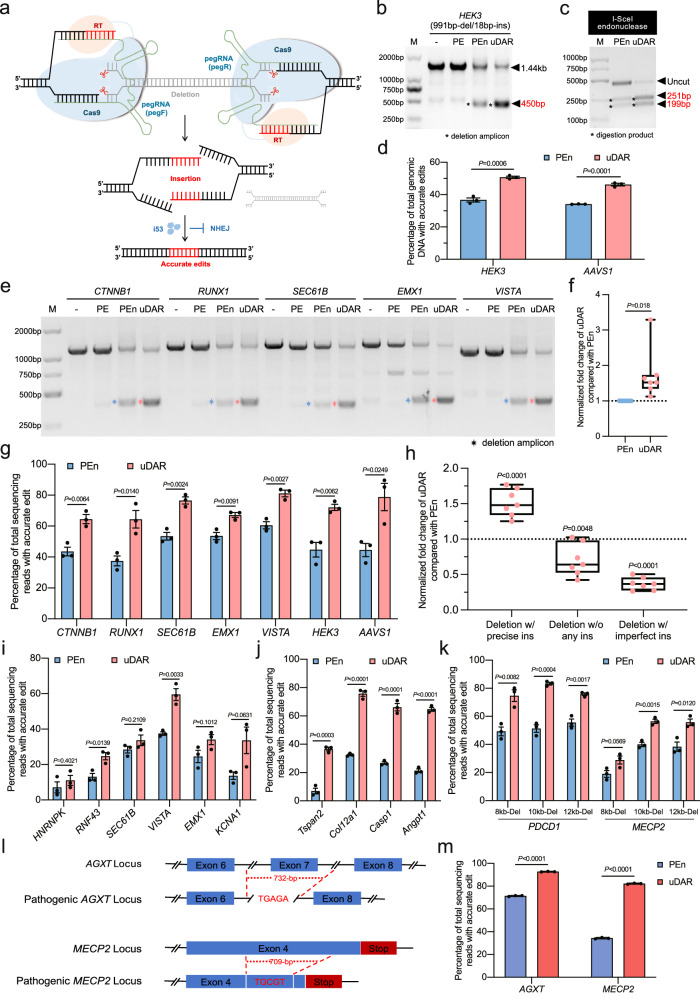
Enhancement of large-fragment deletions by increasing the generation of deletion events and the purity of RT-driven edits using designed uDAR system. **a** Overview of the uDAR system with paired pegRNAs to generate large-fragment deletion and simultaneous insertion edits. **b** Prime editing of a 991-bp DNA fragment deletion and simultaneous insertion of I-SceI recognition sequence (18-bp) at the *HEK3* site with PE, PEn and uDAR. The PCR band of ~450-bp was the intended deletion amplicon (denoted with an asterisk (*)), and the ~1.44-kb band was the amplicon without deletion. **c** PEn- or uDAR-mediated deletion amplicons in **b** were incubated by I-SceI endonuclease and analyzed on a 2% agarose gel. Digested products were marked by arrows. The original amplicon was marked as ‘Uncut’. The band with insertion of I-SceI recognition sequence was denoted with an asterisk (*). **d** PEn- or uDAR-mediated absolute editing efficiencies of all the editing events in total genomic DNA at the *HEK3* or *AAVS1* site via qPCR analysis. **e** The uDAR system induced more deletion amplicons at the five endogenous sites in HEK293T cells. The short PCR bands are the intended deletion amplicon (denoted with an asterisk (*)). **f** Statistical analysis of normalized increase of deletion amplicons for large-fragment deletions by the uDAR and PEn. The mean deletion amplicons induced by PEn for each locus was set to 1, and other samples were normalized correspondingly. *n* = 7 (sites) for each group. **g** Deep sequencing of PEn- or uDAR-induced deletion amplicons showed the percentage of precise edits at five endogenous genomic loci in HEK293T cells. **h** Statistical analysis of normalized increase of prime editing outcomes for large-fragment deletions by the uDAR and PEn. *n* = 7 (sites) for each group. **i**, **j** Deep sequencing of PEn- or uDAR-induced deletion amplicons showed the percentage of precise edits at six endogenous sites in HeLa cells (**i**) and four endogenous sites in N2a cells (**j**). **k** The uDAR system increased the purity of targeted large-fragment deletions ranging from 8 to 12 kb at *PDCD1* and *MECP2* loci in HEK293T cells. **l** Schematic illustration of uDAR generated pathogenic large-fragment deletions simultaneous insertions for the *AGXT* and *MECP2* loci. The pathogenic mutations in *AGXT* involved a 732-bp deletion and “TGAGA” sequence insertion. The pathogenic mutations in *MECP2* involved a 709-bp deletion and “TGCGT” sequence insertion. **m** The editing purity of pathogenic DNA fragment deletion simultaneous insertion at the *AGXT* and *MECP2* sites by PEn and uDAR system in H1 hESCs. For **d**–**m**, data were represented as mean ± s.e.m. (*n* = 3 biologically independent samples). The *P* value was determined by a two-tailed Student’s *t* test.

To characterize the large-fragment editing profiles by uDAR, we first established a stable HEK293T cell line carrying an EGFP reporter (Supplementary Fig. S1b), and then used it to assess the efficiency of PEn-dRT^8^, PEn (representing both WT-PE system^4^ and PEDAR^5^) and uDAR. FACS analysis revealed that, the uDAR triggered 10.80% of EGFP positivity, representing a 1.62-fold increase over PEn (6.66%) (Supplementary Fig. S1c, d). To further evaluate uDAR function at endogenous loci, we targeted *HEK3* and *AAVS1* loci for large-fragment deletion/I-SceI sequence insertion, and compared the editing outcomes among PE (representing PRIME-Del system^9^), PEn and uDAR. Agarose gel electrophoresis of PCR products revealed ~450-bp (*HEK3*) and ~470-bp (*AAVS1*) deletion amplicons in PEn- and uDAR-edited groups, while the PE group showed only the ~1.44-kb full-length control fragment (Fig. 1b and Supplementary Fig. S1e). Following I-SceI digestion of deletion amplicons, agarose gel analysis confirmed precise integration in both systems, but revealed more efficient editing by uDAR compared to PEn (Fig. 1c; Supplementary Fig. S1f). Furthermore, using real-time quantitative PCR (qPCR) (Supplementary Fig. S2), we found that uDAR outperformed PEn in precise editing efficiency, with averages of 50.75% vs 36.70% at *HEK3* locus and 46.20% vs 34.15% at *AAVS1* locus (Fig. 1d), indicating higher precise edits for large-fragment deletions by uDAR.

Notably, the observed more deletion amplicons by uDAR at *HEK3* and *AAVS1* loci suggested that NHEJ inhibition promoted the generation of large-fragment deletion events (Supplementary Fig. S1g). To further substantiate this observation, we characterized deletion amplicons resulting from uDAR at five endogenous loci, and uDAR consistently produced more deletion amplicons across all five sites compared to PEn (Fig. 1e). Statistical analysis revealed uDAR resulted in an average 1.74-fold increase in generating deletion amplicons relative to PEn (Fig. 1f and Supplementary Fig. S3a). Subsequently, we individually amplified each pegRNA target site (pegF/pegR) from the PEn or uDAR treatment and performed Sanger sequencing and TIDE analysis. The results confirmed slightly reduced indel levels at both sites following uDAR treatment (Supplementary Fig. S3b–f). Based on the above results, we suggest a plausible repair mechanism where uDAR enhanced the total deletion events through inhibiting the NHEJ-promoted double-strand break repair at both cleavage sites (Supplementary Fig. S3g).

To further assess the impact of uDAR for large-fragment deletion outcomes, we purified the deletion amplicons and performed deep sequencing analysis (Fig. 1g). At the *HEK3* and *AAVS1* site, both PEn and uDAR systems generated complex mutagenic profiles, including large-fragment deletion with precise insertions, no insertion or imperfect insertions (Supplementary Fig. S4a, b). Genotyping analysis showed that uDAR significantly reduced Cas9-dependent direct deletions and all imprecise insertions. More importantly, it enhanced the purity of accurate deletion–insertion edits to 78.74% at *AAVS1* and 72.09% at *HEK3*, compared to 44.52% and 44.79%, respectively, with PEn (Supplementary Fig. S4c, d). We further assessed the editing purity of uDAR across five endogenous loci (Supplementary Fig. S5a–f), and the analysis showed the uDAR system outperformed PEn, achieving precise edits of 64.52%–81.08% compared to 37.44%–60.5% for PEn (Fig. 1g). Overall, the purity of deletion-insertions mediated by uDAR at all seven endogenous sites was 1.5-fold higher than that of PEn, while direct deletions and imperfect edits were reduced to 0.7-fold and 0.39-fold respectively (Fig. 1h). In summary, uDAR enhanced large-fragment deletion by promoting RT-driven precise edits through the suppression of error-prone NHEJ (Supplementary Fig. S5g).

We next comprehensively profiled large-fragment deletion outcomes by performing PCR-free long-read sequencing in PEn- and uDAR-treated HEK293T cells at *AAVS1* and *CTNNB1* loci (Supplementary Fig. S6a, b). We observed that uDAR consistently mediated higher precise editing efficiency, and uDAR generated more deletion products (both precise and imperfect edits) at both target sites (Supplementary Fig. S6c), which was in strong agreement with our previous gel electrophoresis results. Moreover, among total deletion events, the purity of precise editing was similar to that obtained by short-amplicon PCR sequencing (Supplementary Fig. S6d). Thus, agarose gel electrophoresis and short-amplicon PCR were reliable for detecting large-fragment deletions, whereas PCR-free long-read sequencing enable accurate resolution of the full distribution of individual editing outcomes.

Then, we also benchmarked the performance of uDAR in HeLa cells over six genomic sites. The results showed uDAR yielded more deletion amplicons than PEn and also produced more I-SceI insertions (Supplementary Fig. S7a, b). Deep sequencing confirmed that uDAR mediated accurate deletion ranging from 11.03% to 59.51%, compared to 7.13% to 37.50% for PEn (Fig. 1i), resulting in an overall increase in editing purity of 1.7-fold over PEn (Supplementary Fig. S7c). Additionally, in the N2a cell line, uDAR consistently improved deletion amplicons and I-SceI digestion products compared to PEn at all four sites (Supplementary Fig. S7d, e), and achieved accurate deletion rates ranging from 36.54% to 75.75%, whereas PEn yielded rates ranging from 7.07% to 32.61%, with an overall 3.2-fold enhancement in editing purity (Fig. 1j and Supplementary Fig. S7f).

Next, we evaluated larger genomic deletions (8–100 kb) across multiple loci in HEK293T cells. uDAR consistently outperformed PEn for deletions (8–12 kb) at the *PDCD1* and *MECP2* loci, as confirmed by gel electrophoresis and deep sequencing (Fig. 1k and Supplementary Fig. S8a). At the *DMD* and *RUNX1* loci, uDAR demonstrated higher efficiency and precision than PEn in generating large deletions (25–100 kb), as validated by gel electrophoresis and deep sequencing (Supplementary Fig. S8b–g). Interestingly, uDAR maintained similar high-purity editing across diverse deletion lengths, likely attributable to the NHEJ suppression mediated by i53^6^. Beyond precise deletion, uDAR also outperformed PEn in accurate insertion after deletions, achieving purities ranging from 68.23% to 81.78% across diverse insertion lengths (44–78 bp) compared to 28.66%–42.91% by PEn (Supplementary Fig. S9a, b). Notably, the length of the 3’ extension could influence large-fragment editing outcomes^9,10^. To further explore this, we designed two additional dual-pegRNA strategies (TW and PD) (Supplementary Fig. S9c) and compared the efficiencies of uDAR, TW, and PD in HEK293T cells at three endogenous loci targeting ~1-kb deletions with diverse insertions lengths (32–62 bp). Agarose gel electrophoresis of PCR products consistently revealed similar deletion amplicons across all three systems (Supplementary Fig. S9d). Nevertheless, deep sequencing indicated that no single system consistently outperformed the others across all three loci (Supplementary Fig. S9e). To further explore the effect of 3′ extensions length in dual pegRNAs on uDAR editing purity, we designed 3′ extensions with homologous region (HR) lengths ranging from 5 to 40 bp and compared editing purity across different HR lengths, as well as TW and PD, at four endogenous loci (Supplementary Fig. S10a). The results showed that uDAR consistently improved deletion products and editing purity across all extension lengths and all four sites compared to PEn (Supplementary Fig. S10b–e). Notably, within the 5–20 bp HR range, editing purity increased with increasing HR length across all four loci. However, when the HR length extended to 20–40 bp and TW design, uDAR showed comparable editing purity at the *AAVS1* and *RUNX1* loci, while a clear reduction was observed at the *HEK3* and *SEC61B* loci. Statistical analysis showed that editing purity increased with HR sizes from 5 to 15 bp and reached a plateau between 15 and 25 bp, which was higher than that observed for TW and PD designs (Supplementary Fig. S10f). Therefore, we recommend a general optimal HR length of approximately 20 bp for uDAR design to achieve efficient performance, while noting that the outcome remains influenced by target-site context and that parallel testing of different design configurations may facilitate identification of the best-performing setup for a given locus.

Furthermore, we specifically evaluated uDAR’s performance in non-coding genomic regions. Similarly, uDAR outperformed PEn in generating precise 24-kb and 26-kb deletions at lncRNA loci, achieving accurate deletion–insertion rates of 77% and 85%, whereas PEn yielded rates of 50.69% and 23.67%, respectively (Supplementary Fig. S11a, b). We then extended our application of uDAR to H1 human embryonic stem cell lines (H1 hESCs), a fundamental platform for disease modeling^11^. As expected, uDAR successfully simulated two pathogenic large-fragment deletions with synchronous insertions at the *AGXT* and *MECP2* loci (Fig. 1l), achieving high-precision editing with purities of 92.75% and 82.24%, compared to 71.45% and 34.30% for PEn (Fig. 1m and Supplementary Fig. S11c), demonstrating its potential for creating disease models in hESCs.

Given that uDAR uses dual pegRNA-guided Cas9 cleavage to generate paired DSBs, raising potential safety concerns regarding off-target cleavage^12^, we evaluated potential off-target effects by predicting six candidate sites (allowing up to 4 mismatches^13^) for each pegRNA across four targets (Supplementary Figs. S12a, d, S13a, d). Deep sequencing analyses of off-target sites showed the uDAR induced mostly similar levels of off-target effects to PEn (Supplementary Figs. S12b, c, e, f, S13b, c, e, f). Furthermore, we generated monoclonal cell lines following editing at the *CTNNB1* and *AAVS1* loci by PEn or uDAR (Supplementary Fig. S14a). WGS analysis revealed that uDAR did not lead to an increased number of off-target editing events compared to PEn (Supplementary Fig. S14b–e). To further assess uDAR-induced chromosomal aberrations, we analyzed PCR-free long-read sequencing results within ~20-kb windows flanking the target sites at the *AAVS1* and *CTNNB1* loci (Supplementary Fig. S15a, b). While no additional large deletions were detected at *AAVS1* locus with either method, uDAR induced two distinct off-target deletions at the *CTNNB1* locus (red dashed box) (Supplementary Fig. S15b), suggesting that uDAR fidelity may be influenced by local chromatin context or sequence features. Quantification of various structural variants revealed that uDAR exhibited a similar overall frequency of chromosomal aberrations as PEn alone (Supplementary Fig. S15c, d). Together, these findings indicated that uDAR did not increase pegRNA-dependent small off-target edits or induce genome instability, although a more complex spectrum of byproducts may be generated at certain genomic loci.

In summary, our work established uDAR as a robust and versatile large-fragment deletion genome editing system with broad utility in functional studies and disease modeling, while further investigation is warranted to evaluate its potential for clinical translation.

## Supplementary information

Supplementary Methods, Figures and Tables
