# Supplementary material for: Improvement of precise large-fragment deletion with a versatile nuclease prime editor
Source: Cell Discov. 2026 Aug 14;12:62. doi: 10.1038/s41421-026-00923-3 (PMC13476446; doi:10.1038/s41421-026-00923-3)

Supplementary Information

Improvement of precise large-fragment deletion with a versatile nuclease prime editor

Xiangyang Li[1,*], Wenjun Sun[2,*], Gefei Wang[1], Xinpeng Ma[1], Mengyao Dai[1], Yang Zhao[1], Liqun Lei[1], Xuechao Cai[1], Shuaifeng Li[1], Shisheng Huang[2], Xingxu Huang[1,2,#], Xia Lin[1,#], Tingbo Liang[1,3,#]

[1]The Key Laboratory of Pancreatic Diseases of Zhejiang Province, The First Affiliated Hospital, Zhejiang University School of Medicine, Hangzhou, 310003, China

[2]School of Life Science and Technology, ShanghaiTech University, 100 Haike Rd., Pudong New Area, Shanghai, 201210, China

[3]Department of Hepatobiliary and Pancreatic Surgery, The First Affiliated Hospital, Zhejiang University School of Medicine, Hangzhou 310003, China

[*]These authors contributed equally: Xiangyang Li, Wenjun Sun

[#]Correspondence: Xingxu Huang (huangxx@shanghaitech.edu.cn); Xia Lin (linxia1935@outlook.com); Tingbo Liang (liangtingbo@zju.edu.cn)

Materials and Methods

Cell culture, transfection and harvest

HEK293T (ATCC CRL-3216), N2a (ATCC CCL-131) and HeLa (ATCC CCL-2) cell lines were cultured in Dulbecco's Modified Eagle's Medium supplemented with 10% fetal bovine serum and 1% penicillin/streptomycin at 37 °C with 5% CO_2. The H1 hESCs was kindly provided by Dr. Zhaobo Lin at ShanghaiTech University and was used in accordance with institutional guidelines and applicable regulations for human embryonic stem cell research. H1 hESCs were cultured in mTeSR™ 1 Basal Medium at 37 °C with 5% CO2. For plasmid transfection, HEK293T, N2a or HeLa cells were seeded in 24-well cell culture plate a day before and transfected at about 70% density using lipofectamine 2000 (Thermo Fisher Scientific) twice [2 μl/μg] the amount of plasmid DNA as per the manufacturer's instructions. Cells were transfected with 900 ng of editor plasmids, 300 ng of pegF plasmids and 300 ng of pegR plasmids. For H1 hESCs, cells were seeded in 48-well cell culture plate a day before and transfected at about 50% density using Lipofectamine™ Stem Transfection Reagent (Thermo Fisher Scientific) twice [2 μl/μg] the amount of plasmid DNA as per the manufacturer's instructions. Cells were transfected with 420 ng of editor plasmids, 140 ng of pegF plasmids and 140 ng of pegR plasmids. Three days after transfection, cells were harvested from Fluorescence Activating Cell Sorter (FACS) with $EGFP^+$/$mCherry^+$ selection. A total of 10,000 positive cells were collected by FACS for subsequent genomic DNA preparation.

Flow cytometry analysis

The HEK293T cell lines with stable EGFP reporter were obtained through lentivirus packaging and continuous screening of puromycin. Cells with mCherry marker represented the transfected cells, and cells with EGFP marker represented that the precise editing restored the expression of EGFP protein. Three days after transfection, cells were analyzed for FACS (BD Aria III, programmed via the FACSDiva (8.0.1) software). Transfection of paired pegRNAs along with PEn-dRT, which losing

reverse transcriptional activity served as the negative control for gating. All data were analyzed using FlowJo 10.0 software.

Plasmid construction

To construct EGFP reporter plasmid, the plasmid backbone was amplified from lentiCRISPR v2 (Addgene, #52961) using Phanta® Max Super Fidelity DNA Polymerase (Vazyme). A dead EGFP with inactivated mCherry sequence was synthesized and amplified by PCR and then cloned into EGFP reporter plasmid backbone using ClonExpress Ultra One Step Cloning Kit V2 (Vazyme). Complete sequences for some key constructs are listed in the section of **Supplementary Notes**.

The pegRNA plasmid was constructed according to the reported protocol partially[1]. To construct pegF plasmids, the plasmid backbone was amplified from pGL3-U6-sgRNA-EGFP (Addgene, #107721) using Phanta® Max Super Fidelity DNA Polymerase (Vazyme). The backbone amplicon was then cut by BsaI-HFv2 (NEB) for overhangs. Spacer oligos of pegRNAs (the top strand oligo includes 5′ ACCG and 3′ GTTTT overhangs, while the bottom strand oligo comprises a 5′ CTCTAAAAC overhang), pegRNA 3′ extension (the top strand oligo included 5′ GTGC overhang while the bottom strand oligo included 5′ AAAA overhang), and sgRNA scaffold oligos (featuring compatible overhangs) were synthesized by Tsingke Biotechnology. Next, the four fragments are assembled with T4 DNA Ligase (NEB). For pegR plasmids, the plasmid backbone was amplified from pGL3-U6-sgRNA-mCherry (Addgene, #159788), and pegR plasmids was generated in the same way as above. Sequences for pegRNAs used in the research are listed in **Supplementary Table S1 and S2**. Primers used are listed in **Supplementary Table S3**.

PEmax plasmid was purchased from Addgene (Addgene, #174820). For PEn plasmid construction, oligos were synthesized and the H840A Cas9 mutation in the PEmax construct was reverted to the original histidine[2]. For PEn-dRT editor plasmid construction, R110S and K104L mutations in the RT region were introduced into PEn plasmid[3]. For uDAR construction, sequences encoding P2A and i53 were synthesized by GeneScript, then they were cloned into PEn plasmid to generate uDAR plasmids,

respectively. Complete sequences for some key constructs are listed in the section of **Supplementary Notes**.

Genomic DNA extraction, amplification and digestion

The genomic DNA was extracted using QuickExtract™ DNA Extraction Solution (Lucigen) according to manufacturer's protocols. Target sequences were amplified using Phanta® Max Super-Fidelity DNA Polymerase (Vazyme). Primers used are listed in **Supplementary Table S4**. PCR products were analyzed by electrophoresis on 1% agarose gels, and target amplicons were extracted using a DNA extraction kit (Axygen). The band intensity was analyzed by ImageJ software. Two hundred nanograms of purified PCR products were incubated with I-SceI endonuclease (NEB) according to manufacturer's instructions. One hour after incubation, the products were visualized and analyzed by electrophoresis in 2% agarose gels.

Quantification of total genomic DNA to evaluate the absolute edits

qPCR was used to determine the absolute edits in total genomic DNA at the *HEK3* site or *AAVS1* site according to the reported protocol[4], employing a commercial reaction mixture from Vazyme (AceQ qPCR SYBR Green Master Mix [Low ROX Premixed]). Specific primers were designed to target the deletion region (P1 and P2), span the deletion boundary (P3 and P4), or bridge the deletion/insertion junction (P5 and P6) **(Supplementary Fig. 2a)**. Primers used are listed in **Supplementary Table S5**. Two synthetic DNA fragments were generated (corresponding to the unedited wild-type and the precise edited sequence) and serially diluted, using as standard templets **(Supplementary Fig. 2b)**. qPCR assays were performed using the defined primer sets and templates, resulting in the establishment of three distinct standard curves, which illustrated the correlation between qPCR cycle numbers and the concentrations of DNA representing the unedited, other edited, or accurate edited states **(Supplementary Fig. 2c, d)**. Subsequently, three separate qPCR reactions were carried out using edited genomic DNA as the template, along with the respective primer combinations (P1 + P2, P3 + P4, or P5 + P6). These standard curves were then applied

to quantify the absolute copy numbers of total genomic DNA. The absolute editing rates were computed using the following formulas: accurate editing rate = copy number of DNA with accurate deletion-insertion / copy number of DNA with and without deletion.

Targeted deep-sequencing

To quantify the proportion of accurate edits generated by different editors at the single-nucleotide level, deep sequencing analysis of sequences with large-fragment deletions was required. Using the specific primers listed in **Supplementary Table S6** (spanning approximately 100-bp on either side of the cut sites), the target fragments were amplified from total genomic DNA using Phanta® Max Super-Fidelity DNA Polymerase (Vazyme). For the analysis of the same site under different experimental conditions, 5' primers with distinct barcodes were used to generate amplicons. Long amplicons without deletions were removed by gel electrophoresis. PCR products with different barcodes were pooled together, and biological replicates were combined into separate sequencing samples. Gel-purified PCR products underwent end repair and adaptor ligation according to the manufacturer's instructions (Illumina). Deep sequencing experiments were conducted using the Illumina NovaSeq platform (PE150) at Novogene. Sequencing reads were demultiplexed using AdapterRemoval (v.2.2.2), and paired-end reads with an alignment length of 11 bp or more were merged into single consensus reads. All processed reads were mapped to the target sequences using the BWA-MEM algorithm (BWA v.0.7.17). The level of precise editing was determined by calculating the percentage of reads containing the target edited sequence with perfect alignment relative to the total number of mapped reads.

Whole-genome sequencing

DNA extracted from harvested cells was sequenced using Illumina NovaSeq (PE150) at Novogene. All cleaned reads were mapped to the human reference genome (GRCh38/hg38) using BWA v.0.7.17 with default parameters. Sequence reads were removed for duplicates using Sambamba v.0.6.7. Variants were identified by GATK (v.4.1.8.1) HaplotypeCaller and filtered with the following criteria: (1)

sequencing depth (for each individual) > 1/3× and < 3×; (2) variant confidence/quality by depth > 2; (3) RMS mapping quality (MQ) > 40.0; (4) Phred-scaled *P* value using Fisher's exact test to detect strand bias < 60; (5) Z-score from the Wilcoxon rank sum test of Alt vs. Ref read MQs (MQRankSum) > −12.5; and (6) Z-score from the Wilcoxon rank sum test of Alt vs. Ref read position bias (ReadPosRankSum) > −8. Reference sequencing data from un-transfected cells were used to filter pre-existing mutations. Potential off-target sites were predicted by Cas-OFFinder[5]. For long-read sequencing analyses, PCR-free libraries were prepared from genomic DNA of sorted cells and sequenced on the PacBio HiFi platform (Genewiz). Raw HiFi reads were demultiplexed using lima (v2.7.0) with dual-index barcodes and aligned to the GRCh38 reference genome using pbmm2 (v1.13.0). Sequencing depth was calculated using samtools (v1.19.2), and coverage plots were generated with R (v4.2.3). Structural variants were identified using sniffles (v2.2), and on-target deletion outcomes were manually inspected using IGV.

ClinVar data analysis

The ClinVar variant summary was obtained from the NCBI ClinVar database (accessed 8 April 2024). Variants with pathogenic significance were filtered by allele ID to remove duplicates. All pathogenic or likely pathogenic variants were categorized according to mutation type. The fractions of distinct mutation types were calculated using Origin2021.

Statistics and reproducibility

Targeted deep-sequencing data were processed with the CRISPResso2[6] algorithm in batch mode using default parameters. All quantitative data presented were based on three biological replicates. Analyses and graphing were performed using GraphPad Prism v.9. Data are presented as mean ± s.e.m. as indicated in the figure legends. Statistical significance of differences between groups was determined using two-tailed Student's t-test. In box plots, the center line represents the median, and the box limits correspond to the upper and lower quartiles.

Ethics statement

This study used the established H1 hESCs, which was kindly provided by Dr. Zhaobo Lin at ShanghaiTech University. No human participants were involved, and no new human biological materials were collected. The use of H1 hESCs was conducted according to institutional guidelines.

Data Availability

The NCBI ClinVar database is accessible at https://www.ncbi.nlm.nih.gov/clinvar/. Targeted amplicon sequencing, WGS and long-reads sequencing data have been deposited to the NCBI-SRA repository under BioProject number: PRJNA1184455 and PRJNA1483649. All other data are available upon reasonable request.

References

1 Anzalone, A. V. *et al.* Search-and-replace genome editing without double-strand breaks or donor DNA. *Nature* **576**, 149-157 (2019).

2 Li, X. *et al.* Development of a versatile nuclease prime editor with upgraded precision. *Nat. Commun.* **14**, 305 (2023).

3 Peterka, M. *et al.* Harnessing DSB repair to promote efficient homology-dependent and -independent prime editing. *Nat. Commun.* **13**, 1240 (2022).

4 Jiang, T., Zhang, X.-O., Weng, Z. & Xue, W. Deletion and replacement of long genomic sequences using prime editing. *Nat. Biotechnol.* **40**, 227-234 (2022).

5 Bae, S., Park, J. & Kim, J. S. Cas-OFFinder: a fast and versatile algorithm that searches for potential off-target sites of Cas9 RNA-guided endonucleases. *Bioinformatics* **30**, 1473-1475 (2014).

6 Clement, K. *et al.* CRISPResso2 provides accurate and rapid genome editing sequence analysis. *Nat. Biotechnol.* **37**, 224-226 (2019).

7 Brinkman, E. K., Chen, T., Amendola, M. & van Steensel, B. Easy quantitative assessment of genome editing by sequence trace decomposition. *Nucleic Acids Res.* **42**, e168 (2014).

8 Anzalone, A. V. *et al.* Programmable deletion, replacement, integration and inversion of large DNA sequences with twin prime editing. *Nat. Biotechnol.* **40**, 731-740 (2022).

9 Zhuang, Y. *et al.* Increasing the efficiency and precision of prime editing with guide RNA pairs. *Nat. Chem. Biol.* **18**, 29-37 (2022).

10 Choi, J. *et al.* Precise genomic deletions using paired prime editing. *Nat. Biotechnol.* **40**, 218-226 (2022).

Supplementary Figures

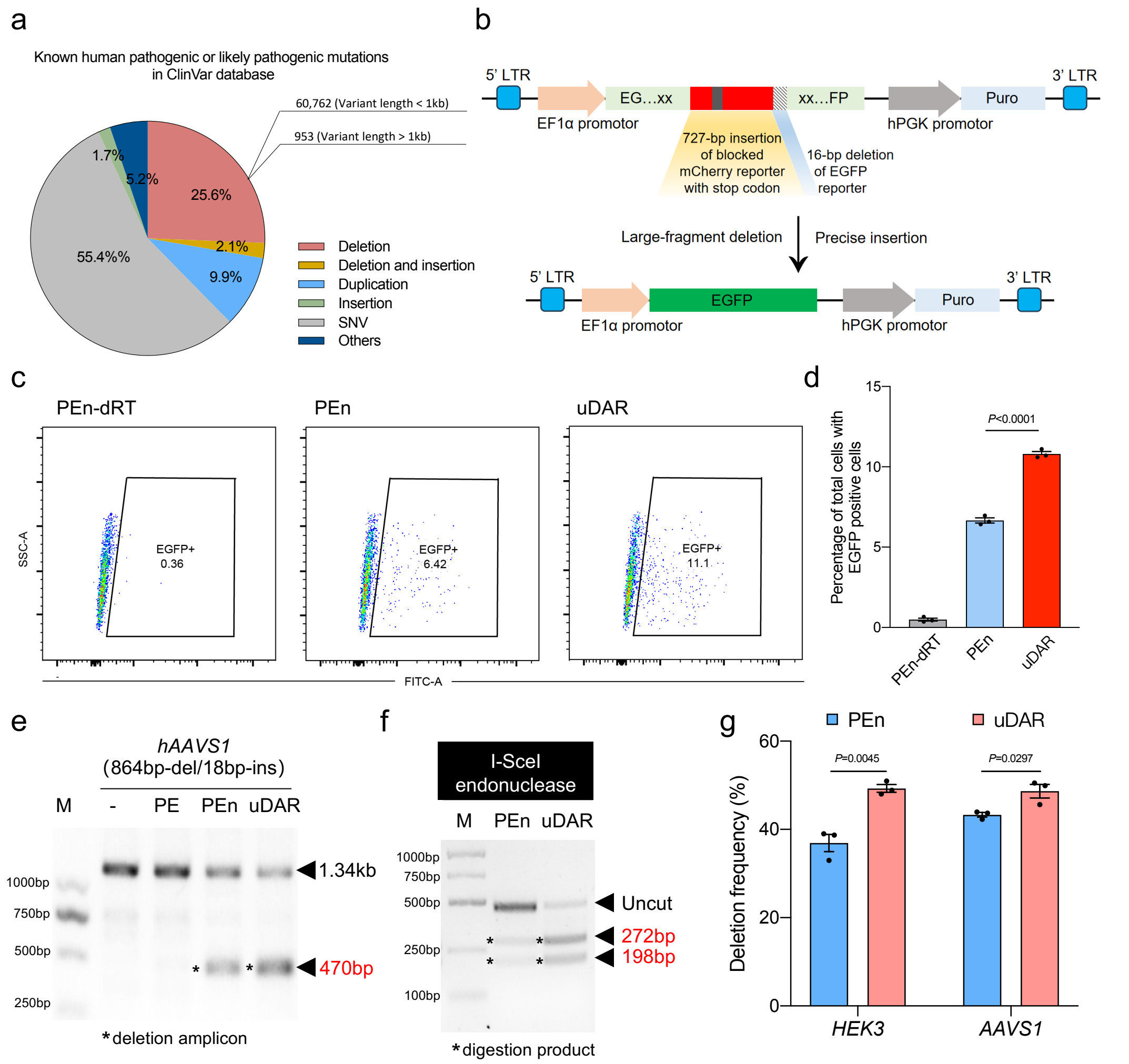

Supplementary Figure 1. The designed uDAR demonstrated enhanced large-fragment deletion and simultaneous insertion at reporter and endogenous genomic loci.

a. Classification of the 241,074 known human pathogenic or likely pathogenic genetic variants reported in the ClinVar database; SNV, single-nucleotide variation.

b. Schematic diagram of the fluorescent prime editing reporter system. EGFP sequence is interrupted by deletion of the 16-bp sequence and simultaneous insertion of the inactivated mCherry sequence. uDAR with paired pegRNAs restores the whole EGFP sequence by precise excrescent fragment deletion and simultaneous 16-bp EGFP sequence insertion.

c. A representative flow cytometry analyses of cells with EGFP reporter treated with PEn-dRT, PEn, and uDAR, respectively. PEn-dRT, which introduced with R110S and

K104L mutations that inactivate the reverse transcriptional activity and abolish prime editing, co-transfected with paired pegRNAs served as the negative control.

d. Following prime editing of EGFP reporter with paired pegRNAs along with PEn-dRT, PEn, or uDAR, the frequencies (%) of $EGFP^+$ were analyzed by flow cytometry. Data are represented as mean ± s.e.m. (n= 3 biologically independent samples). *P* values (directly marked on the graph) were determined by a two-tailed Student's t-tests.

e. Prime editing of a 864-bp DNA fragment deletion and simultaneous insertion of I-SceI recognition sequence (18-bp) at the *AAVS1* site with PE, PEn or uDAR. The PCR band of ~470-bp was the intended deletion amplicon, and the ~1.34-kb band was the amplicon without deletion.

f. PEn- or uDAR- mediated deletion amplicons in (e) were incubated by I-SceI endonuclease and analyzed on a 2% agarose gel. Digested products were marked by arrows. The original amplicon was marked as 'Uncut'. The band with insertion of I-SceI recognition sequence was denoted with an asterisk (*).

g. Quantification analyses of the deletion products by ImageJ software of the band intensity at the *HEK3* or *AAVS1* site. Error bars represented s.e.m. of three replicates. The *P* value (directly marked on the graph) was determined by a two-tailed Student's t-test.

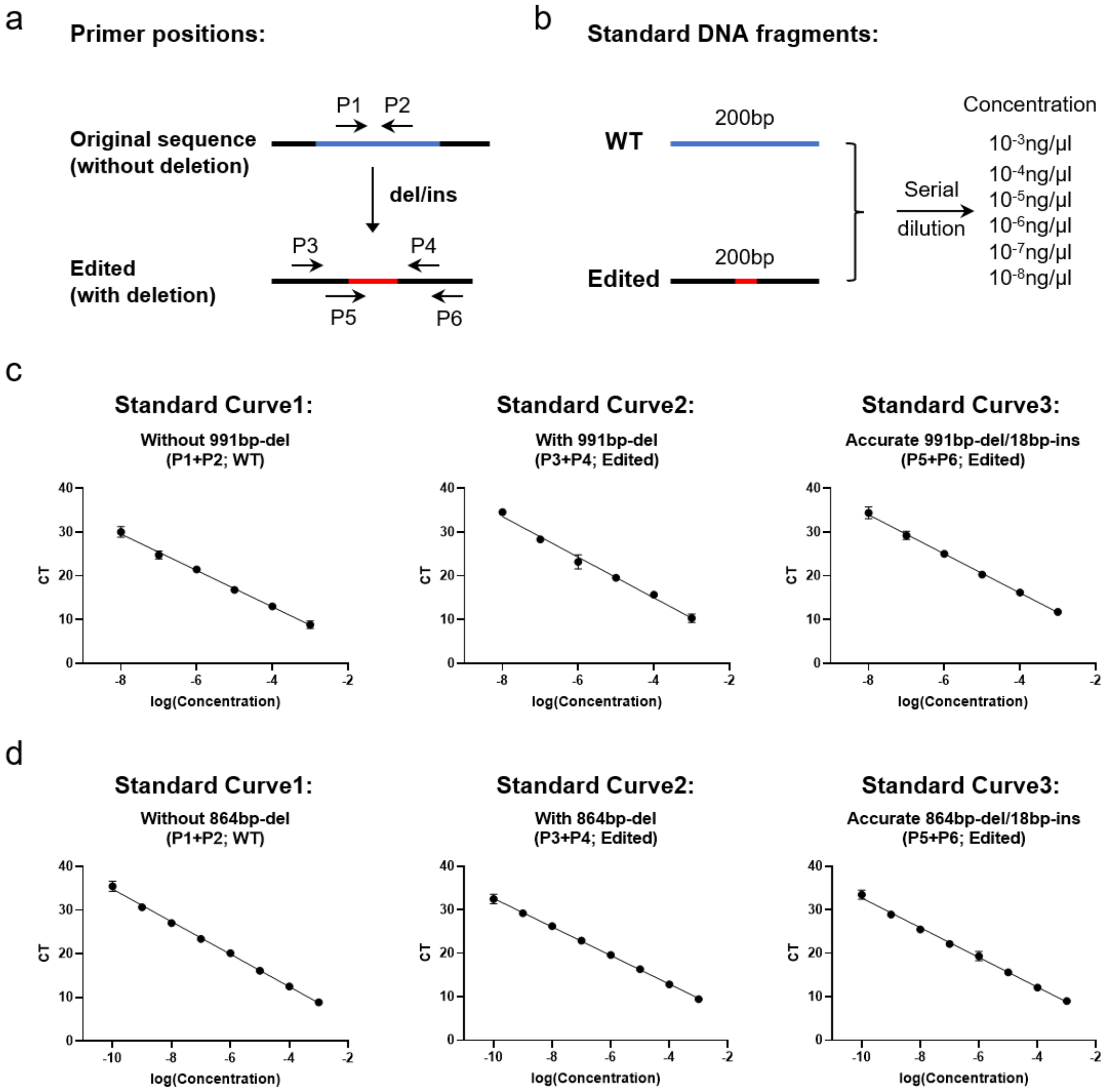

Supplementary Figure 2. The design of absolute quantification with genome DNA.

a. Three pairs of qPCR primers are designed and used to amplify respective target sequences.

b. The unedited or precisely edited target sequence are synthesized with the same length of 200-bp, labeled "WT" and "edited" respectively, and are used as the template for the standard curve after gradient dilution.

c, d. Three standard curves were generated by qPCR using specified paired primers (a) and template (b). The curves reflect the correlation between qPCR cycle number targeted *HEK3* (c) or *AAVS1* (d) sites and the DNA concentration without deletion (standard curve 1), with deletion (standard curve 2), or with accurate insertion of 18-bp after deletion (standard curve 3).

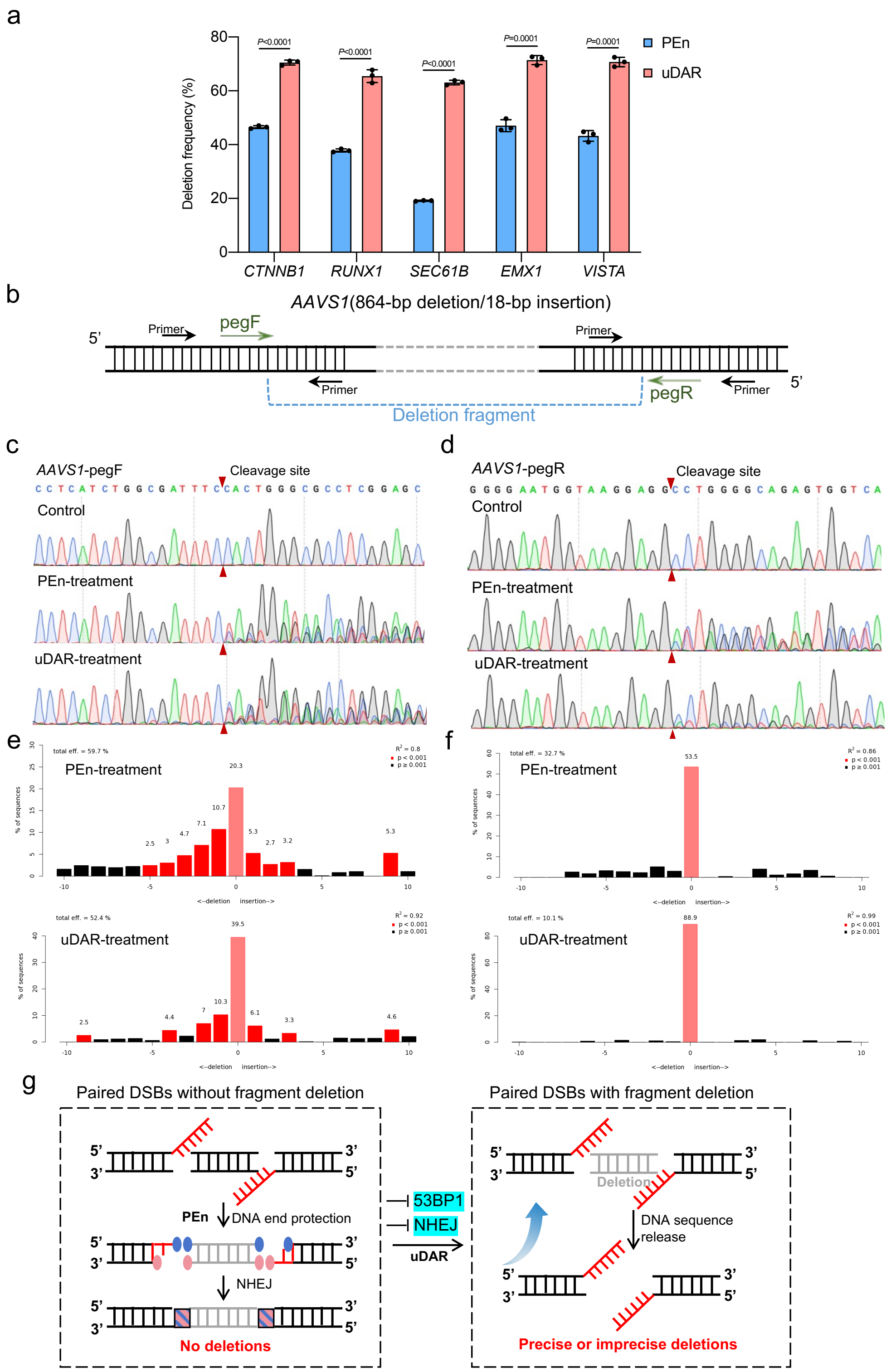

Supplementary Figure 3. The uDAR enhanced large-fragment deletion by increasing formation of deletion products at multiple endogenous sites.

a. Quantification analyses of the deletion amplicons by ImageJ software of the band intensity (deletion frequency (%) = size-normalized small band intensity/size-

normalized total intensity). Error bars represented s.e.m. of three replicates. The *P* value (directly marked on the graph) was determined by a two-tailed Student’s t-test.

b. Schematic illustration of primers for analyzing the edit outcomes of PEn or uDAR in the genomic targets of single pegRNA (pegF or pegR).

c, d. Sanger sequencing shows the editing outcomes of PEn and uDAR in pegF (c) and pegR (d) cleavage sites at *AAVS1* site. The target genomic region is assayed and sequencing by PCR amplification across pegF or pegR cut sites, respectively.

e, f. The indel analysis of PEn- or uDAR- mediated edits without deletion at pegF and pegR cleavage sites in (c, d) by TIDE web tool[7].

g. The potential mechanisms underlying uDAR-dependent large fragment deletion through increasing formation of deletion products. uDAR with paired pegRNAs induces paired DSBs and generates RT-driven extensions, then the deletion fragment is released, generating the potential intermediates. Note that the red segments within the overhang structure are homologous and depict the inserted sequence. If the DSBs is repaired prior to the overhang annealing, the NHEJ pathway will lead to indel formation at two cut sites instead of fragment deletion. The i53 activity in uDAR will inhibit NHEJ and stimulate the cleavage of the broken DNA ends, resulting in more deletion products.

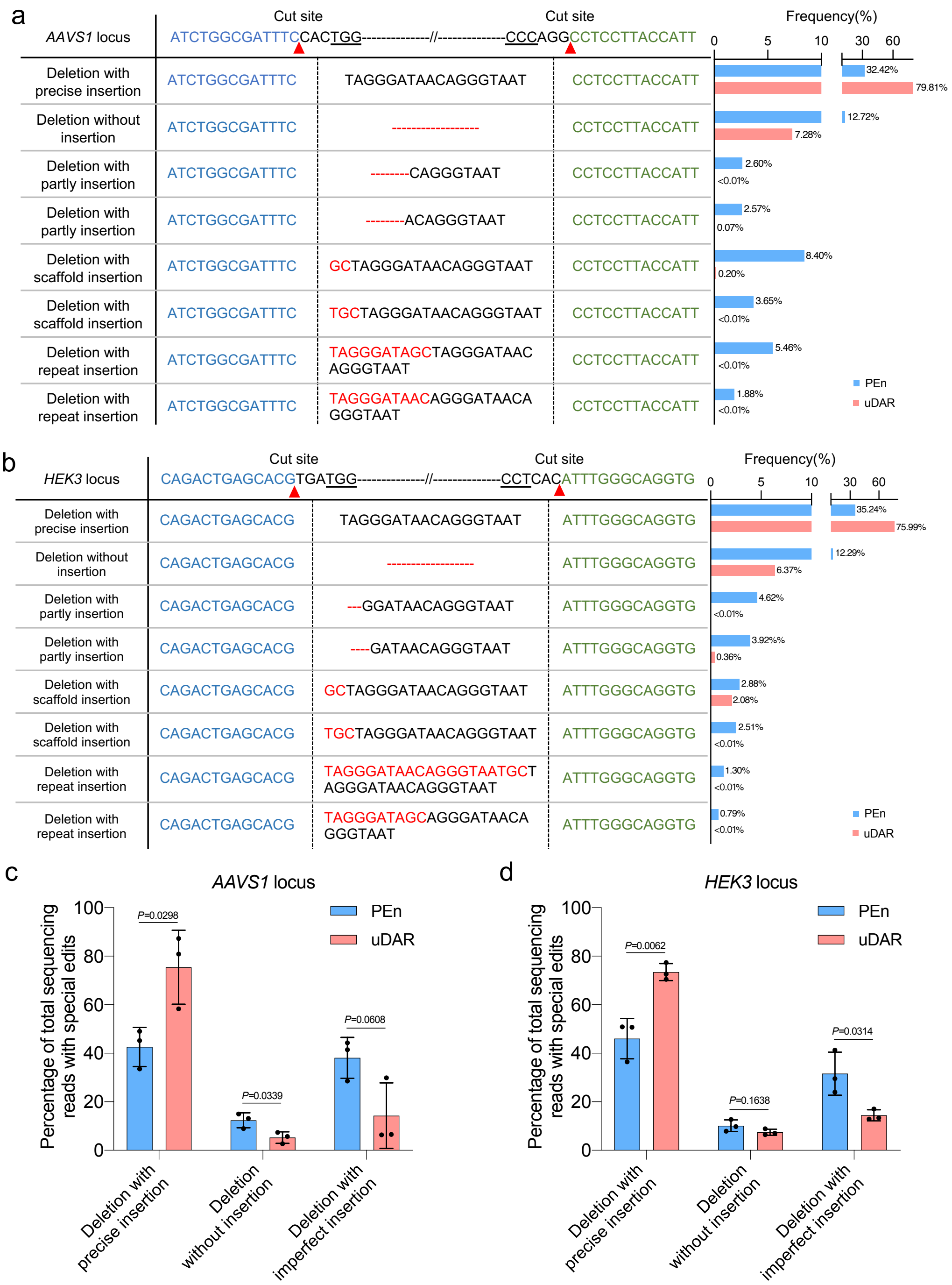

Supplementary Figure 4. Statistical analysis of editing outcomes by PEn/uDAR at *AAVS1* and *HEK3* sites for large fragment deletions.

a, b. Comparative analysis of large-fragment deletion profiles by PEn and uDAR at *AAVS1* and *HEK3* sites. DNA sequence of the target was edited to produce a range of synthetic outcomes that employ the PEn or uDAR. Deletion with precise insertions, deletion without insertions and top six imperfect edits were showed in the list of deletion outcomes, and the frequencies corresponding to these genotypes were showed on the

right. The 18-bp intended insertion was in black, and the imperfect edits were highlighted in red. PBS sequences of pegF and pegR were in blue and green. The PAM sequences were underlined.

c, d. The proportions of deletion with precise insertion, deletion without insertion and deletion with imperfect insertion generated by PEn or uDAR at *AAVS1* (a) or *HEK3* site (b). Deletions with precise insertion represent accurate edits. Deletions without any insertion represent direct deletions. Deletions with imperfect insertion represent imprecise edits. Data are represented as mean ± s.e.m. (n= 3 biologically independent samples). The *P* value is determined by a two-tailed Student's t-test.

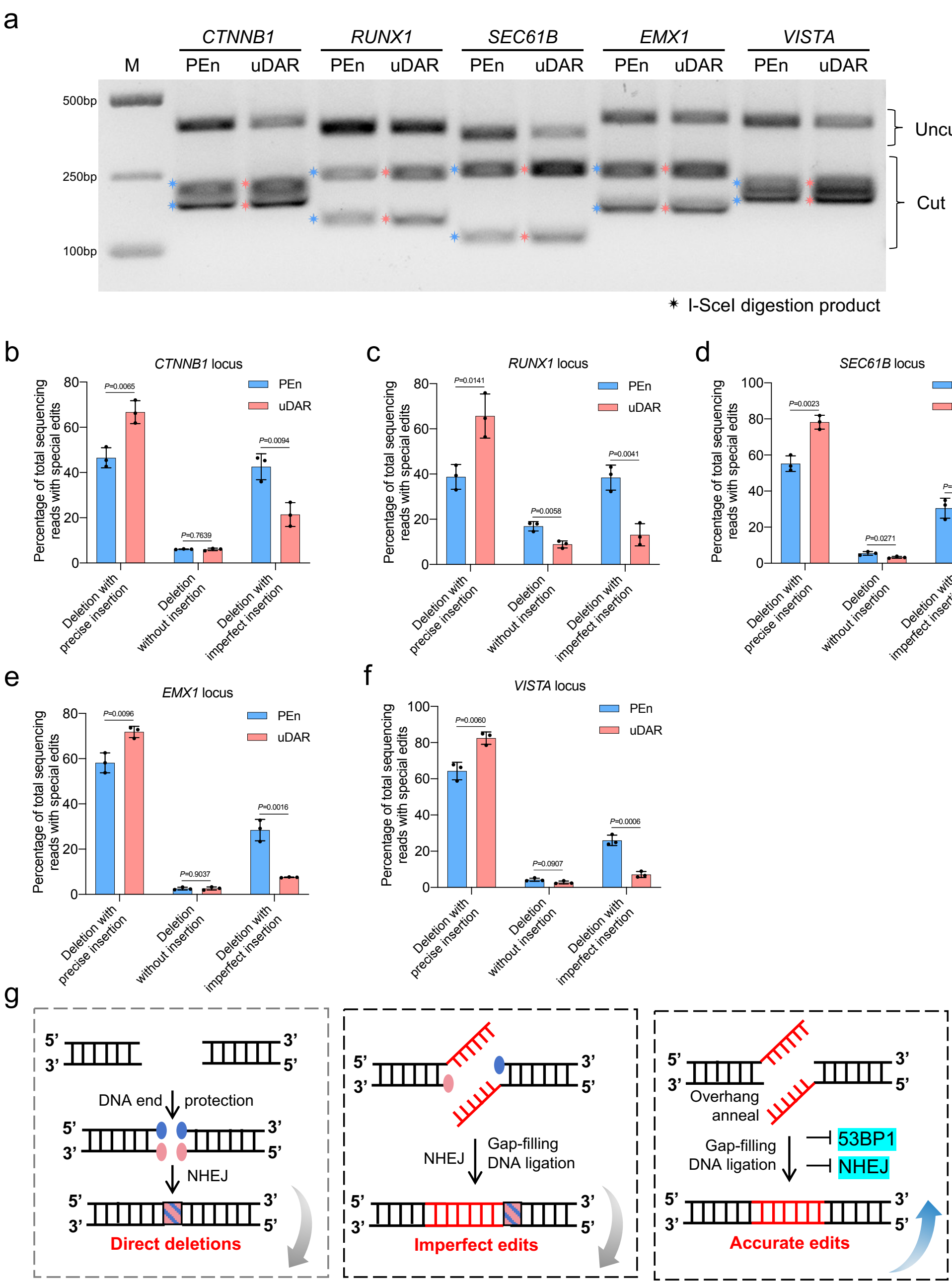

Supplementary Figure 5. The uDAR enhanced large-fragment deletion by increasing the purity of RT-driven edits at multiple endogenous genomic loci.

a. PEn- or uDAR- mediated deletion amplicons at multiple endogenous genomic loci are incubated by I-SceI endonuclease and analyzed on a 2% agarose gel. Digested products are marked by arrows. The original amplicon is marked as 'Uncut'. The band with insertion of I-SceI recognition sequence is denoted with an asterisk (*).

b-f. Deep sequencing of PEn- or uDAR- induced deletion amplicons showed the precise and imprecise edits at *CTNNB1* (b), *RUNX1* (c), *SEC61B* (d), *EMX1* (e) and

VISTA (f) sites in HEK293T cells. Data were represented as mean ± s.e.m. (n= 3 biologically independent samples). The *P* value was determined by a two-tailed Student's t-test.

g. The potential mechanisms underlying uDAR-dependent large fragment deletion through increasing the purity of RT-driven edits. The paired overhangs are annealed and the DNA gap is ligated to achieve accurate edits with desired insertions. However, the paired DSBs repaired through NHEJ pathway prefers to induce imperfect edits. Similarly, the 53BP1 blockade by uDAR can effectively increase the purity of RT-driven edits, generating more accurate edits.

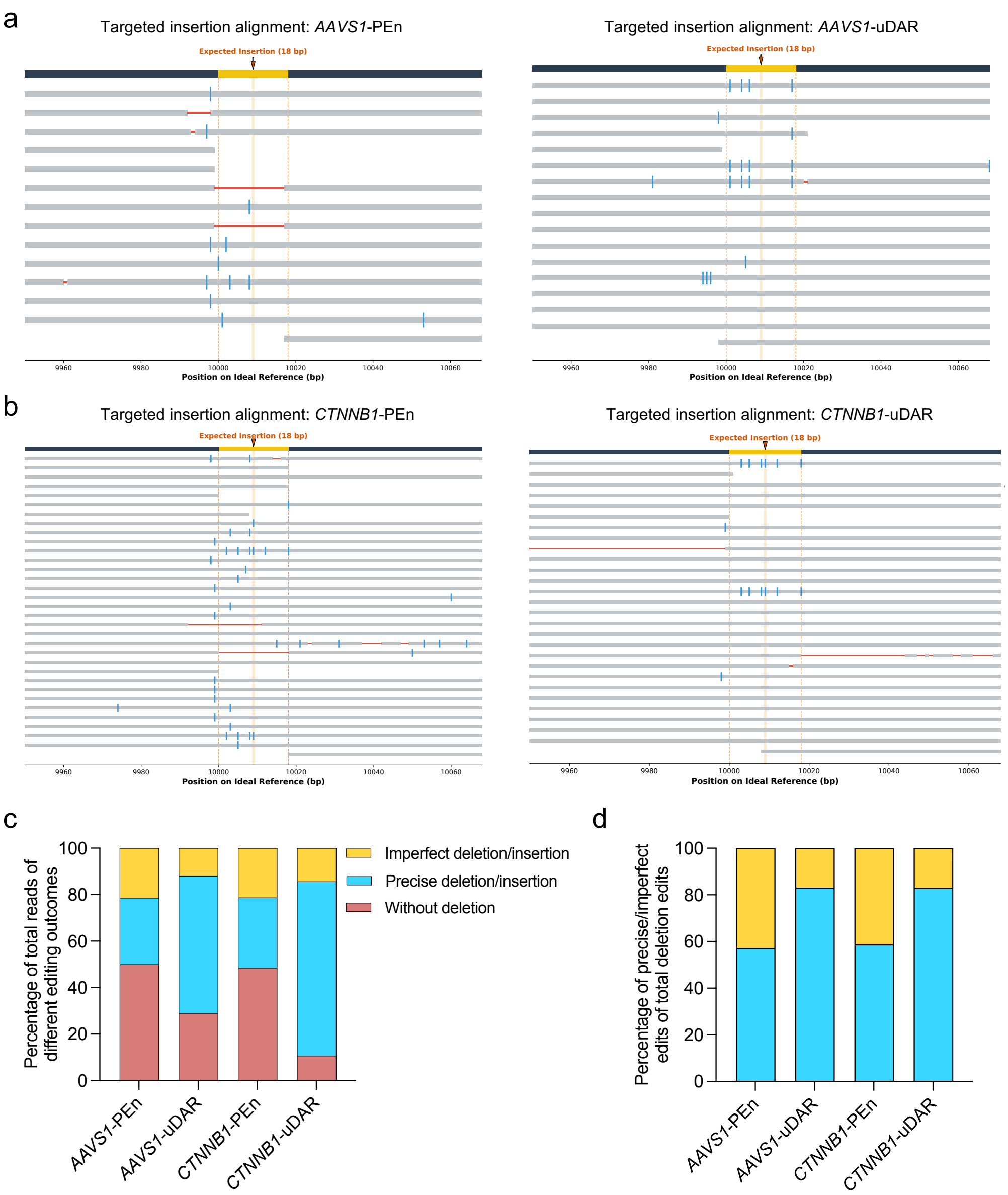

Supplementary Figure 6. Assessment of overall editing outcomes mediated by PEn or uDAR at the *AAVS1* and *CTNNB1* sites in HEK293T cells.

a, b. Target deletion/insertion analysis of PEn and uDAR at *AAVS1* (a) and *CTNNB1* (b) sites. The expected insertion has been clearly marked. Black lines indicate the ideal editing outcome; gray boxes indicate the matched region; red lines indicate the deletion sequences; blue lines indicate insertion sequences.

c. Proportions of no deletion, precise deletion/insertion, and imperfect deletion/insertion events among total editing outcomes following PEn and uDAR treatment at both sites.

d. Percentage of precise deletion/insertion and imperfect deletion/insertion events among total deletion edits following PEn and uDAR treatment at both sites.

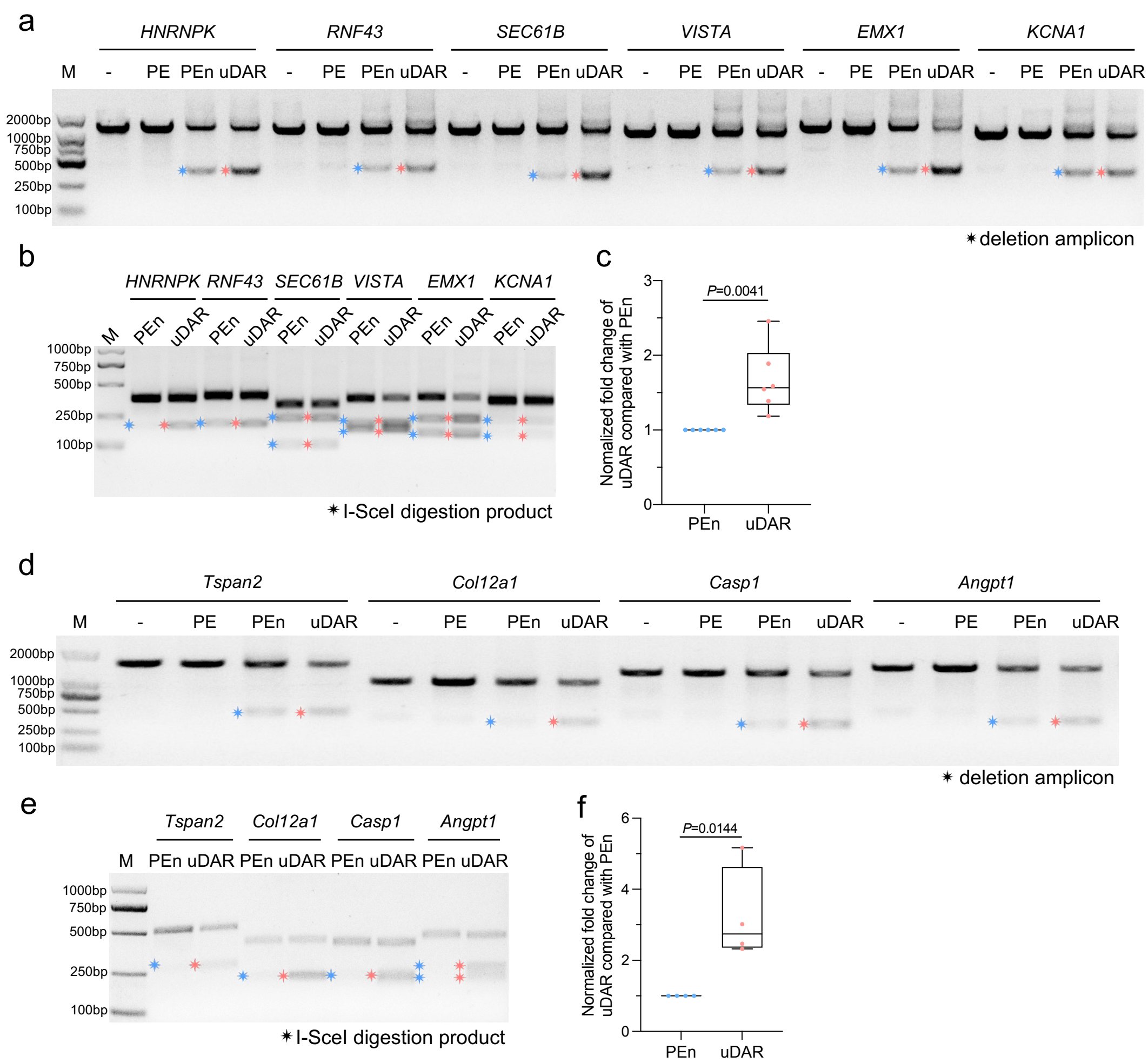

Supplementary Figure 7. The uDAR increased large-fragment deletion efficiencies in multiple mammalian cell lines.

a, d. Prime editing of ~1-kb DNA fragment deletion and simultaneous insertion of I-SceI recognition sequence (18-bp) with PE, PEn or uDAR at six endogenous sites in HeLa cells (a) and four endogenous sites in N2a cells (d). The PCR bands of deletion amplicon were denoted with asterisk (*), in which blue asterisks referred to the PEn-treated group and red asterisks referred to the uDAR-treated group.

b, e. PEn- or uDAR- mediated deletion amplicons in (a) or (d) were incubated by I-SceI endonuclease and analyzed on a 2% agarose gel. Digested products were marked by asterisk (*). The original amplicon was marked as ‘Uncut’. Blue asterisks referred to the PEn-treated group and red asterisks referred to the uDAR-treated group.

c, f. Precise editing rates for large-fragment deletion by the uDAR normalized to the rates by PEn at the sites in (b) or (e). The mean editing frequency induced by PEn for each locus was set to 1, and other samples were normalized correspondingly. The

center line showed medians of all data points and the box limits correspond to the upper the lower quartiles, while the whiskers extend to the largest and smallest values. n = 6 (sites) for HeLa cell group and n = 4 (sites) for N2a cell group. The *P* value was determined by a two-tailed Student's t-test.

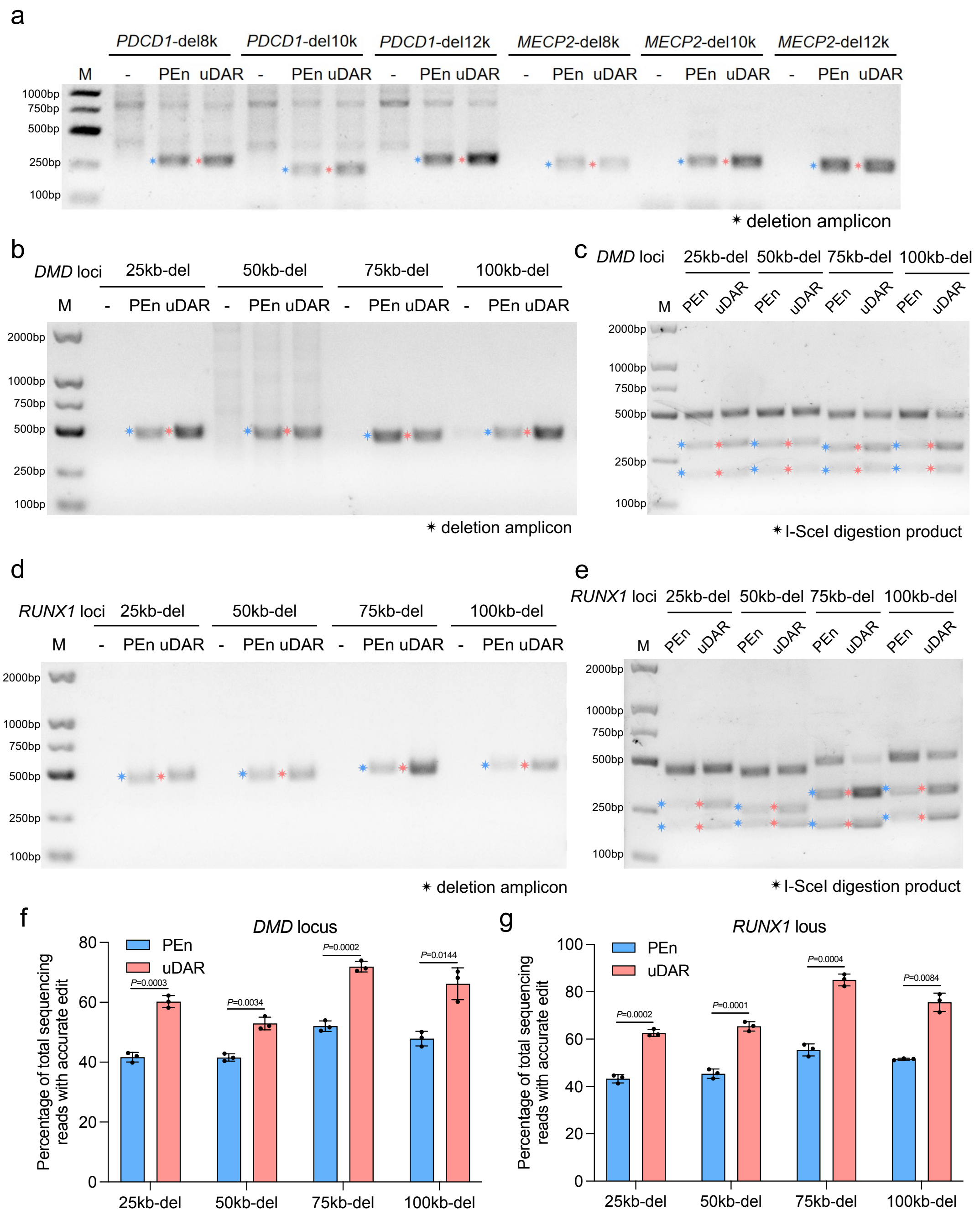

Supplementary Figure 8. The uDAR enable larger-fragment deletion and insertion with enhanced product purity.

a. Prime editing of different DNA fragment deletions ranging from 8-kb to 12-kb with PE, PEn and uDAR at *PDCD1* and *MECP2* sites. The target genomic region is generated by PCR amplification across two cut sites. The short PCR bands are the intended deletion amplicon (denoted with an asterisk (*)), and the long PCR band is the amplicon without deletion.

b, d. Prime editing of different DNA fragment deletions ranging from 25-kb to 100-kb with PEn and uDAR at *DMD* (b) and *RUNX1* (d) sites. The target genomic region is generated by PCR amplification across two cut sites. The short PCR bands are the intended deletion amplicon (denoted with an asterisk (*)).

c, e. PEn- or uDAR- mediated deletion amplicons in (b, d) are incubated by I-SceI endonuclease and analyzed on a 2% agarose gel. Digested products are marked by arrows. The original amplicon is marked as 'Uncut'. The band with insertion of I-SceI recognition sequence is denoted with an asterisk (*).

f, g. Deep sequencing of deletion amplicons generated by PEn and uDAR at the *DMD* (f) and *RUNX1* (g) loci for large deletions. Editing rate represented the reads with accurate editing/total deletion events. Data were represented as mean ± s.e.m. (n= 3 biologically independent samples). *P* values were determined by a two-tailed Student's t-test.

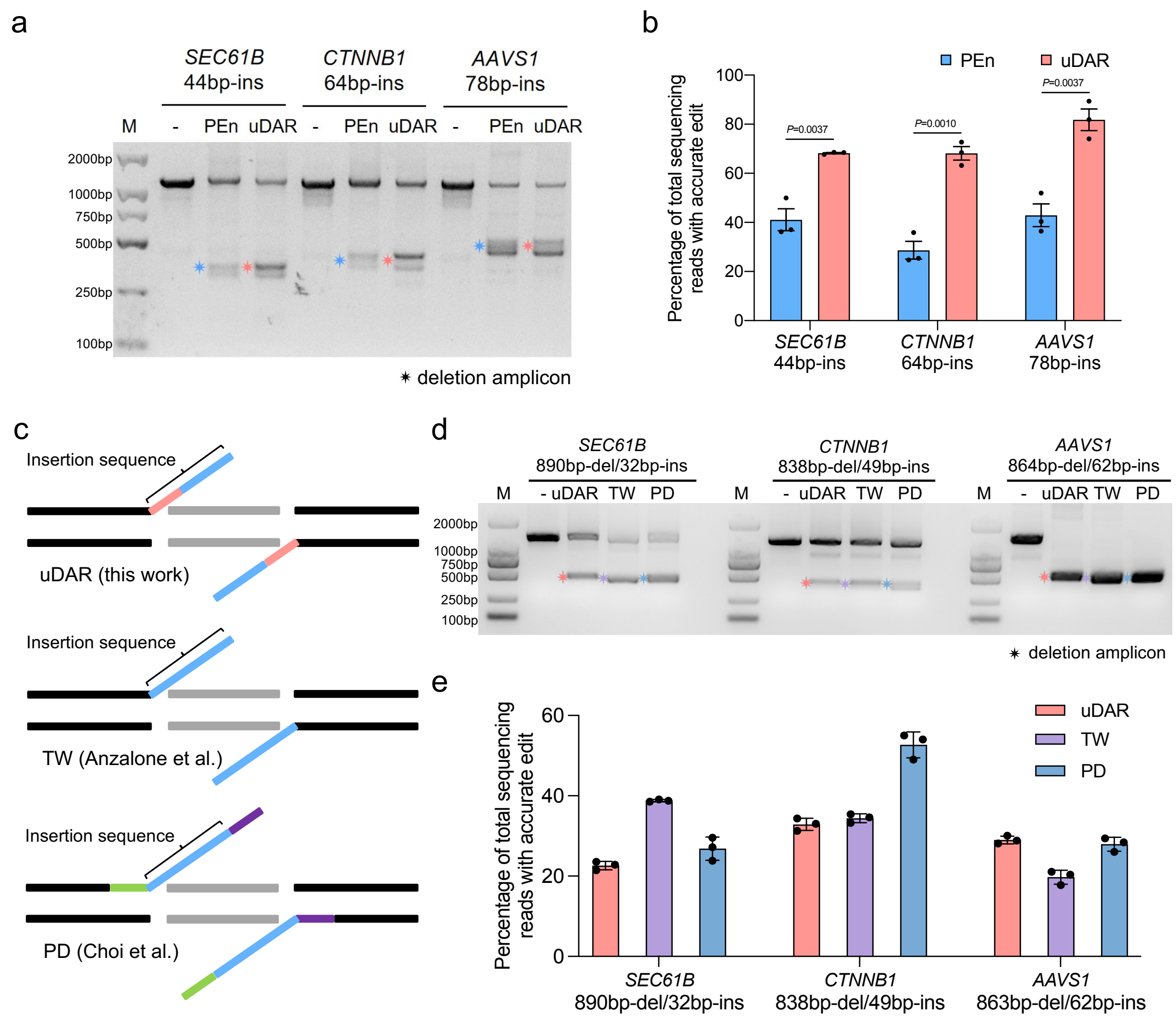

Supplementary Figure 9. The comparison of editing outcomes of uDAR with different 3′ extension lengths.

a. Large-fragment deletion(~1-kb) and insertion of different lengths' sequences (44-bp, 64-bp and 78-bp) were performed in HEK293T cells. The deletion amplicon and the no-deletion amplicon were separated by gel electrophoresis. Band denoted with asterisk (*) was the expected deletion amplicon, where blue referred to the PEn-treated group and red referred to the uDAR-treated group.

b. Deep sequencing of deletion amplicons shown in (a). Editing rate represented the reads with accurate editing/total deletion events. Data were represented as mean ± s.e.m. (n= 3 biologically independent samples). *P* values were determined by a two-tailed Student's t-test.

c. Schematic diagram illustrating designs of paired pegRNA for uDAR, TW and PD system. The TW system refers to uDAR incorporating pegRNA designs from TwinPE[8] or HOPE[9], and the PD system refers to uDAR incorporating pegRNA designs from

PRIME-Del[10] or HOPE. The insertion sequence has been clearly marked. Gray boxes indicate regions where DNA is excised; blue lines indicate the homologous region of heterologous DNA sequence; green and purple lines indicate homologous regions of genomic DNA sequence.

d. Large-fragment deletion(~1-kb) and insertion of different sequences (32-bp, 49-bp and 62-bp) were performed in HEK293T cells using uDAR, TW and PD. The deletion amplicon and the no-deletion amplicon were separated by gel electrophoresis. Band denoted with asterisk (*) was the expected deletion amplicon.

e. Deep sequencing of deletion amplicons shown in (d). Editing rate represented the reads with accurate edits/total deletion events. Data were represented as mean ± s.e.m. (n= 3 biologically independent samples). *P* values were determined by a two-tailed Student's t-test.

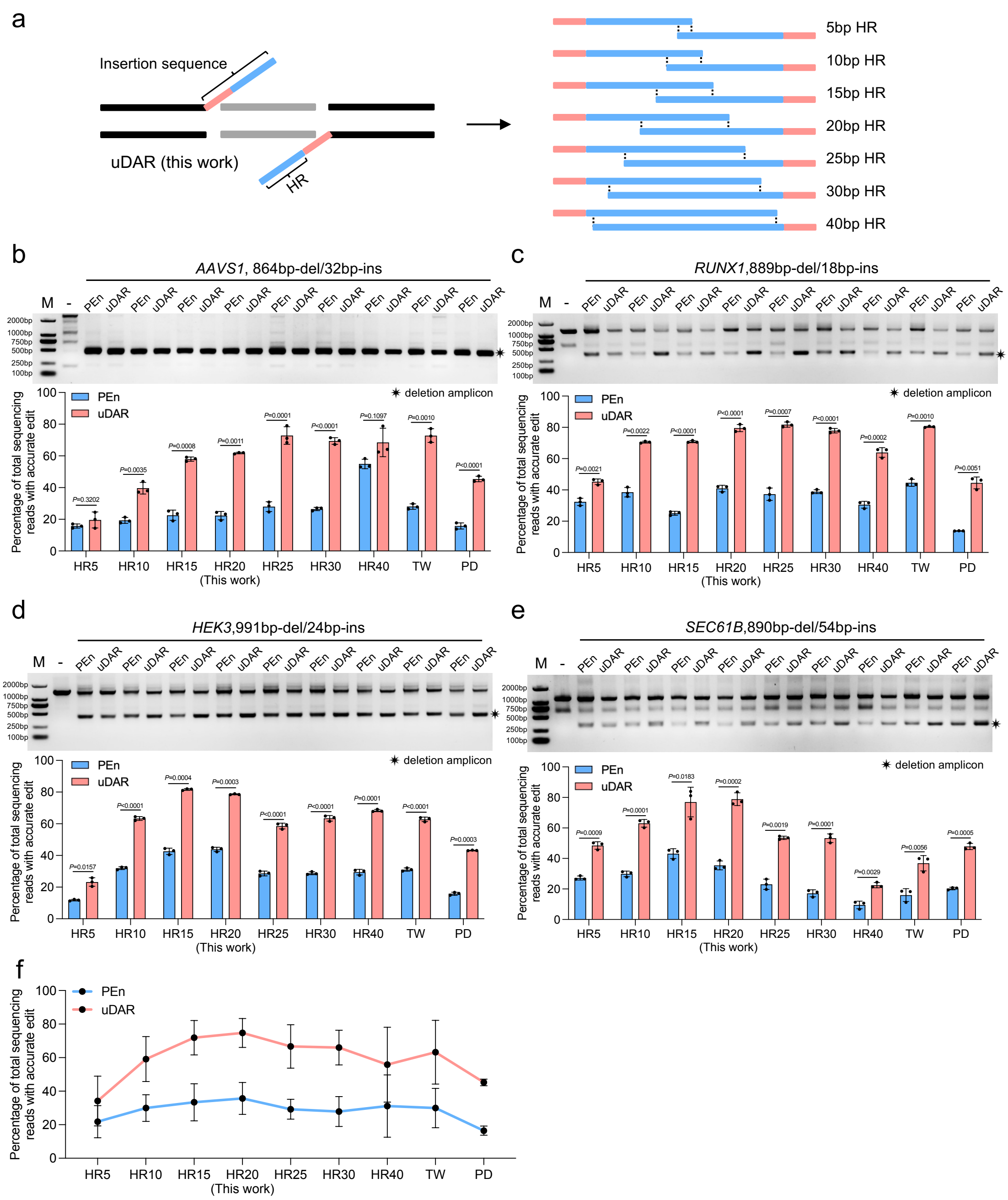

Supplementary Figure 10. Effect of 3′ extensions length in paired pegRNA on PEn and uDAR editing purity.

a. Schematic diagram illustrating designs of paired pegRNA for uDAR with homologous region lengths ranging from 5 to 40 bp.

b-e. Large-fragment deletion(~1-kb) and insertion of different sequences at *AAVS1* (b), *RUNX1* (c), *HEK3* (d) and *SEC61B* (e) loci were performed in HEK293T cells using uDAR (with different HR size), TW and PD. The deletion amplicon and the no-deletion amplicon were separated by gel electrophoresis. Band denoted with asterisk (*) was

the expected deletion amplicon. Deletion amplicons were subjected to deep sequencing, and editing rate represented the reads with accurate edits/total deletion events. Data were represented as mean ± s.e.m. (n= 3 biologically independent samples). *P* values were determined by a two-tailed Student's t-test.

f. The pattern of uDAR editing purity with increasing sizes of HR at four sites in (b-e).

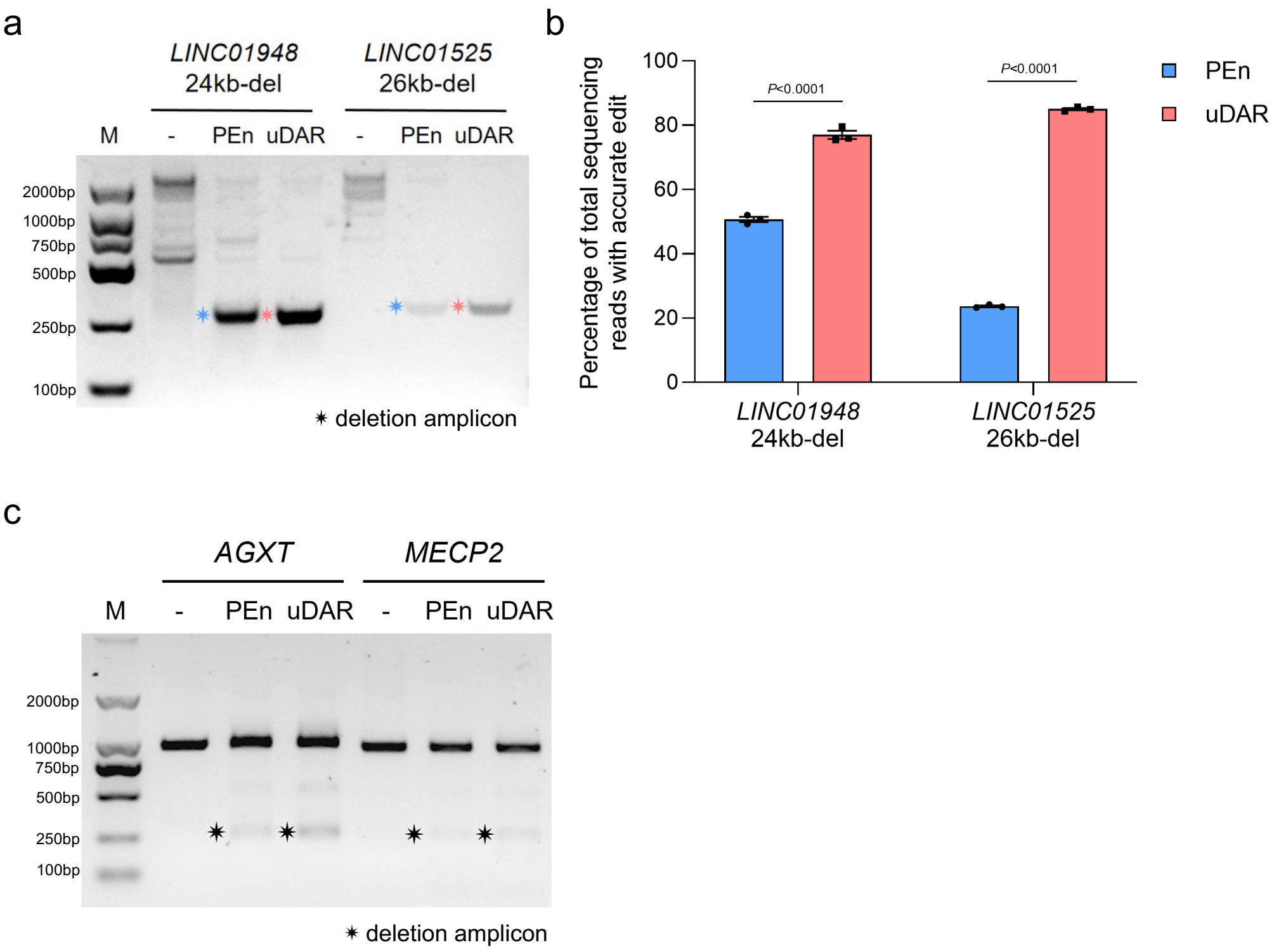

Supplementary Figure 11. The large-fragment deletions of long non-coding RNA (lncRNA) and pathogenic mutations induced by uDAR.

a. Large-fragment deletions and replacements at two lncRNA sites by PEn and uDAR are performed in HEK293T cells. Respectively ~24-kb deletion and 19-bp insertion at *LINC01948*, and ~26-kb deletion and 7-bp insertion at *LINC01525*. The PCR band denoted with asterisk (*) are the desired deletion amplicons.

b. Deep sequencing of PEn- or uDAR- induced deletion amplicons show the percentage of precise edits at the same sites in (a). Data are represented as mean ± s.e.m. (n= 3 biologically independent samples). The *P* value is determined by a two-tailed Student's t-test.

c. Prime editing of pathogenic DNA fragment deletion simultaneous insertion at the *AGXT* and *MECP2* sites by PEn and uDAR in H1 hESCs. Band denoted with asterisk (*) was the expected deletion amplicon.

a

HEK3-pegF	Chr.	Start position	End position
On	chr9	107422339	107422358
OT1	chr15	79457572	79457591
OT2	chr1	46540030	46540049
OT3	chr17	4740507	4740526
OT4	chrX	54951738	54951757
OT5	chr11	44587642	44587661
OT6	chr11	126159759	126159778

HEK3-pegR	Chr.	Start position	End position
On	chr9	107423344	107423363
OT1	chr15	63360022	63360041
OT2	chr5	95342165	95342184
OT3	chr1	30772770	30772789
OT4	chr22	20432325	20432344
OT5	chr2	126169921	126169940
OT6	chr12	132202261	132202280

b

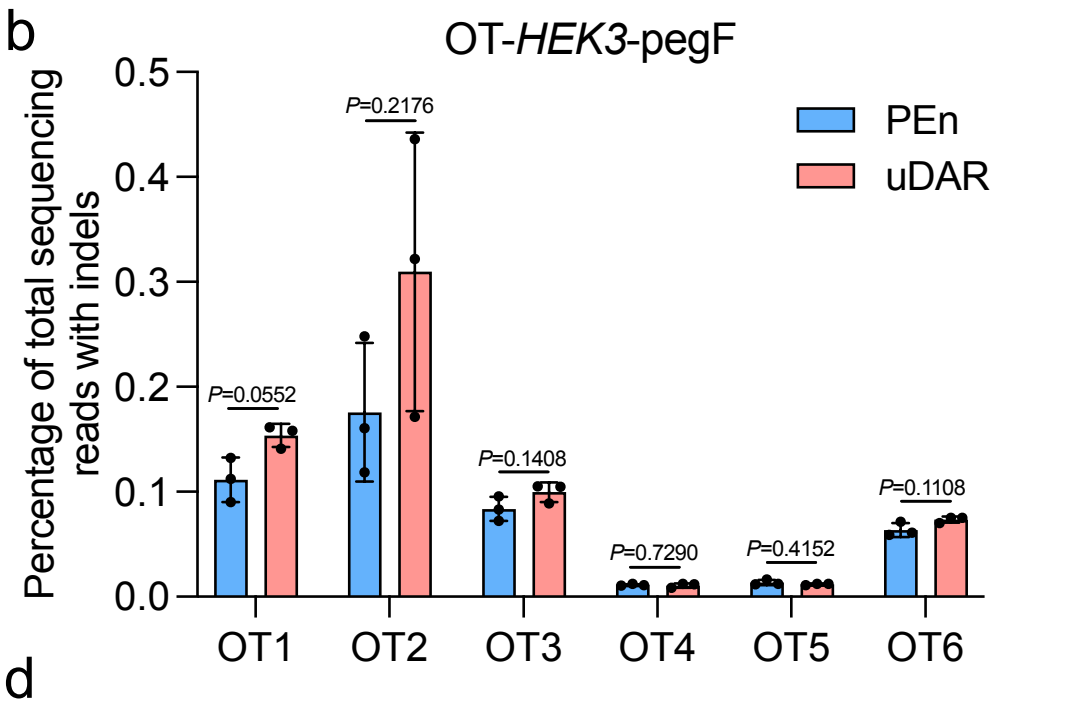

c

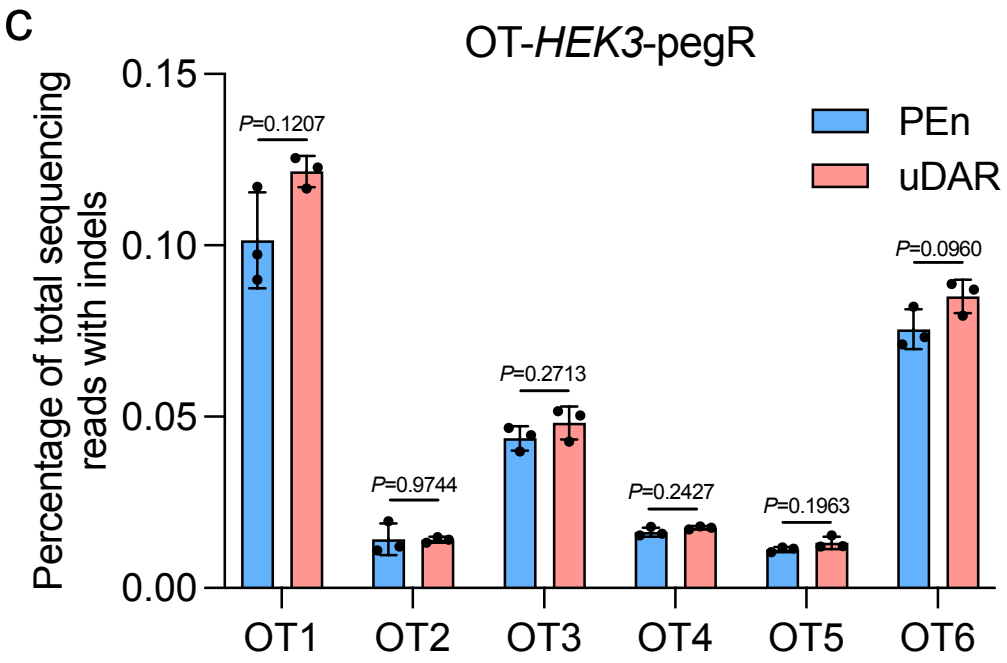

d

RUNX1-pegF	Chr.	Start position	End position
On	chr21	34982962	34982981
OT1	chr20	40649559	40649578
OT2	chr1	41695750	41695769
OT3	chr1	208189754	208189773
OT4	chr14	98892327	98892346
OT5	chr6	22104642	22104661
OT6	chr6	97828155	97828174

RUNX1-pegR	Chr.	Start position	End position
On	chr21	34982059	34982078
OT1	chrX	135172275	135172294
OT2	chr19	54206893	54206912
OT3	chr5	147954267	147954286
OT4	chr20	58405236	58405255
OT5	chr1	3975872	3975891
OT6	chr7	30249470	30249489

e

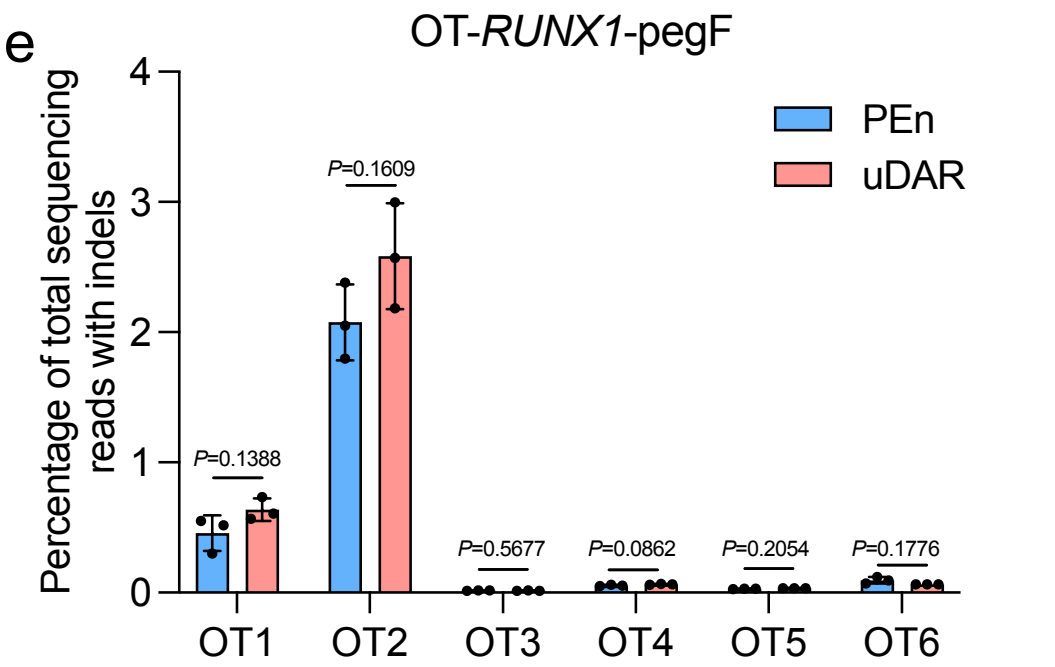

f

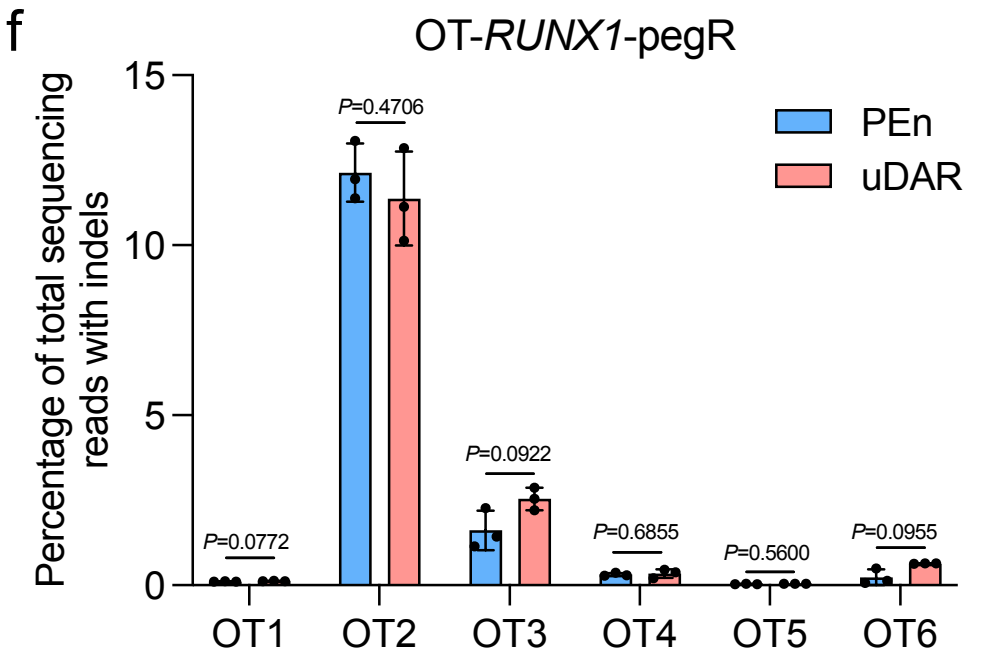

Supplementary Figure 12. Comparison of potential off-target edits of paired pegRNAs for PEn and uDAR at *HEK3* and *RUNX1* loci in HEK293T cells.

a, d. Information for the off-target sites with *HEK3* and *RUNX1 loci* as the desired targets.

b, c, e, f. Off-target analyses for PEn and uDAR-mediated targeting of *HEK3* (c, d) and *RUNX1* (e, f) loci. Values reflect the mean of three biological replicates. Data are

represented as mean ± s.e.m. (n= 3 biologically independent samples). The *P* value is determined by a two-tailed Student's t-test.

a

CTNN-pegF	Chr.	Start position	End position
On	chr3	41233645	41233664
OT1	chr19	28836433	28836452
OT2	chr7	43337717	43337736
OT3	chr4	25421891	25421910
OT4	chr9	108929453	108929472
OT5	chr10	7334139	7334158
OT6	chr10	115969059	115969078

CTNN-pegR	Chr.	Start position	End position
On	chr3	41234497	41234516
OT1	chr5	8701289	8701308
OT2	chr1	181995253	181995272
OT3	chr2	7348300	7348319
OT4	chr2	235986169	235986188
OT5	chr4	124242757	124242776
OT6	chr17	42853909	42853928

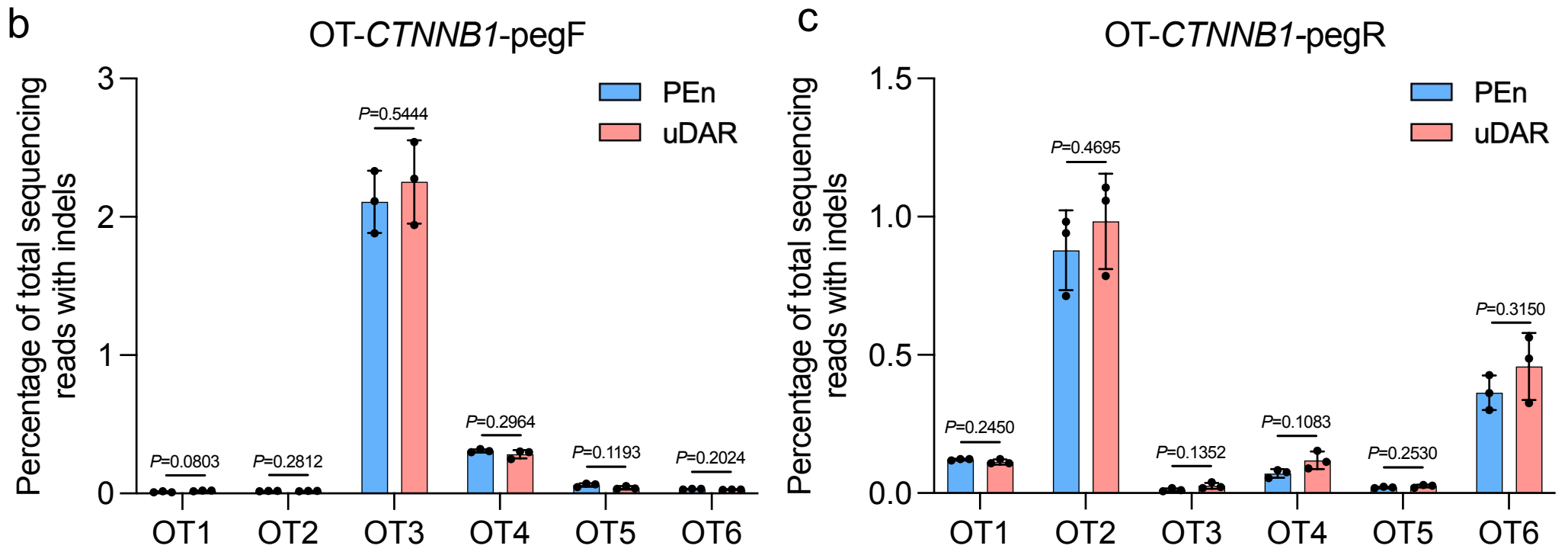

d

SEC-pegF	Chr.	Start position	End position
On	chr9	99223981	99224000
OT1	chr1	109785498	109785517
OT2	chr2	185630478	185630497
OT3	chr5	16654938	16654957
OT4	chr8	21668306	21668325
OT5	chr4	5277986	5278005
OT6	chr20	53413044	53413063

SEC-pegR	Chr.	Start position	End position
On	chr9	99224885	99224904
OT1	chr8	135172275	133907209
OT2	chr17	81415717	81415736
OT3	chrX	54710530	54710549
OT4	chr13	95604250	95604269
OT5	chr19	3681630	3681649
OT6	chr18	11416215	11416234

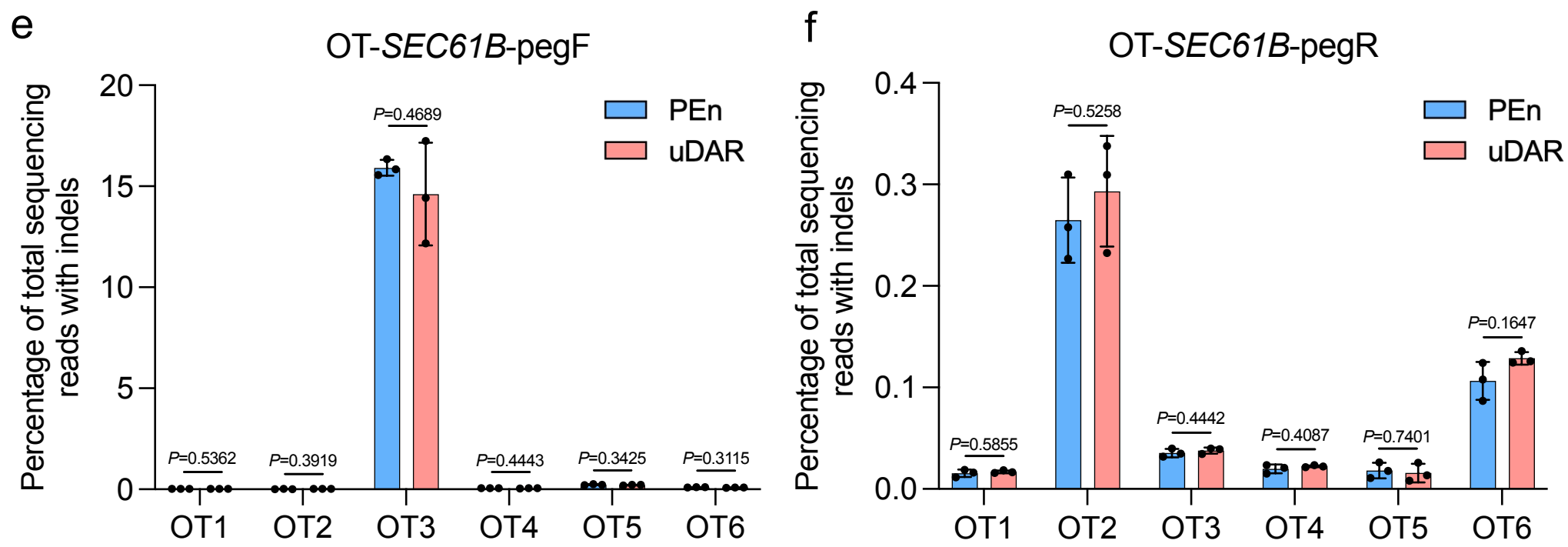

Supplementary Figure 13. Comparison of potential off-target edits of paired pegRNAs for PEn and uDAR at *CTNNB1* and *SEC61B* loci in HEK293T cells.

a, d. Information for the off-target sites with *CTNNB1* and *SEC61B1* loci as the desired targets.

b, c, e, f. Off-target analyses for PEn and uDAR-mediated targeting of *CTNNB1* (c, d) and *SEC61B1* (e, f) loci. Values reflect the mean of three biological replicates. Data

are represented as mean ± s.e.m. (n= 3 biologically independent samples). The *P* value is determined by a two-tailed Student’s t-test.

a

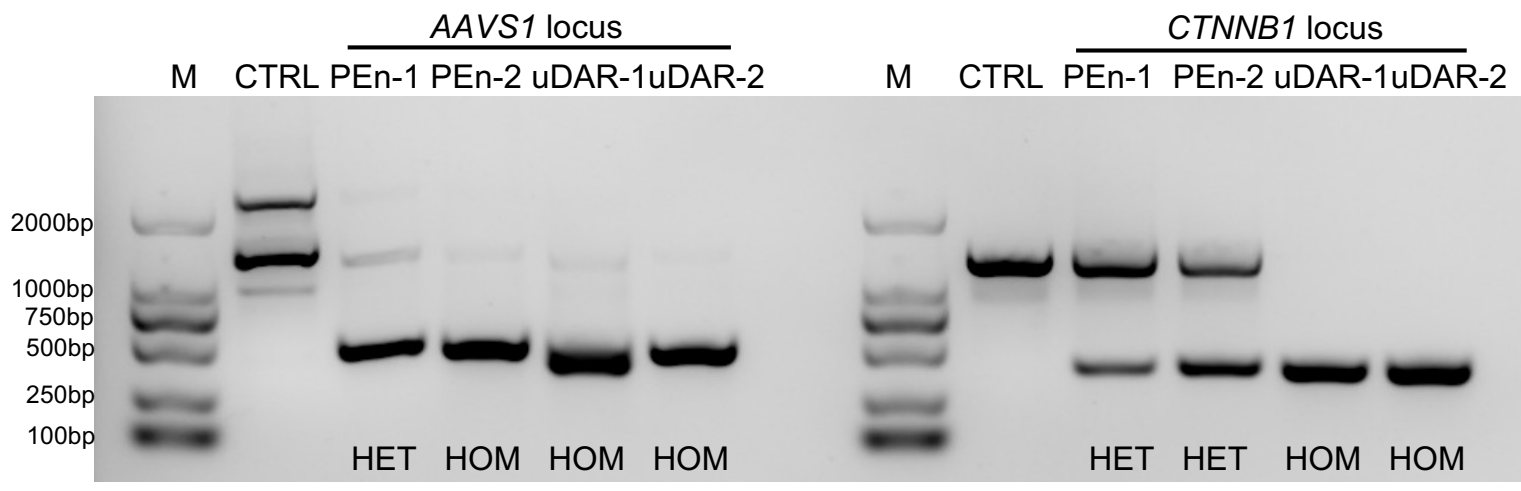

b

Sample	Sequenced reads	Mapping rate	Duplication	Mean depth
AAVS1-ctrl	776,046,328	99.88%	27.04%	34.07X
AAVS1-PEn-1	810,210,198	99.90%	24.82%	35.44X
AAVS1-PEn-2	762,091,528	99.91%	23.80%	36.83X
AAVS1-uDAR-1	878,987,666	99.89%	25.58%	35.57X
AAVS1-uDAR-2	865,141,302	99.90%	25.64%	35.66X

c

Mismatch	Predicted	*AAVS1*-PEn-1	*AAVS1*-PEn-2	*AAVS1*-uDAR-1	*AAVS1*-uDAR-2
1	0/1	0/0	0/0	0/0	0/0
2	0/8	0/0	0/0	0/0	0/0
3	10/52	0/0	0/0	0/0	0/0
4	147/681	0/0	0/0	0/0	0/0
5	1,351/5,146	0/0	0/1	0/0	1/1

d

Sample	Sequenced reads	Mapping rate	Duplication	Mean depth
CTNNB1-ctrl	811,849,410	99.91%	24.76%	32.71X
CTNNB1-PEn-1	1,021,453,804	99.91%	26.18%	41.49X
CTNNB1-PEn-2	832,161,740	99.91%	25.16%	30.56X
CTNNB1-uDAR-1	746,354,476	99.93%	24.71%	33.27X
CTNNB1-uDAR-2	778,251,940	99.92%	23.88%	35.11X

e

Mismatch	Predicted	*CTNNB1*-PEn-1	*CTNNB1*-PEn-2	*CTNNB1*-uDAR-1	*CTNNB1*-uDAR-2
1	0/0	0/0	0/0	0/0	0/0
2	3/0	0/0	1/0	0/0	0/0
3	20/9	0/0	0/0	0/0	0/0
4	486/469	0/1	0/1	1/0	0/0
5	3,818/12,819	4/8	3/7	2/3	1/5

Supplementary Figure 14. WGS analyses of off-target effects associated with PEn and uDAR in monoclonal cells.

a. Gel electrophoresis identification of monoclonal cell lines. HEK293T cells were respectively transfected with EGFP (control), PEn and uDAR. Monoclonal cell lines were obtained through FACS sorting, clonal expansion, and genotyping. HET denotes heterozygous clones, and HOM denotes homozygous clones.

b, d. Whole-genome sequencing sample information. For each locus, one control, two PEn-treated monoclonal cell lines, and two uDAR-treated monoclonal cell lines were included.

c, e. Summary of off-target analysis. Potential off-target sites with up to 5-nt mismatches from the spacer of the targeting pegRNA (pegF/pegR) were predicted by Cas-OFFinder[5] (requiring NRG as PAM).

a

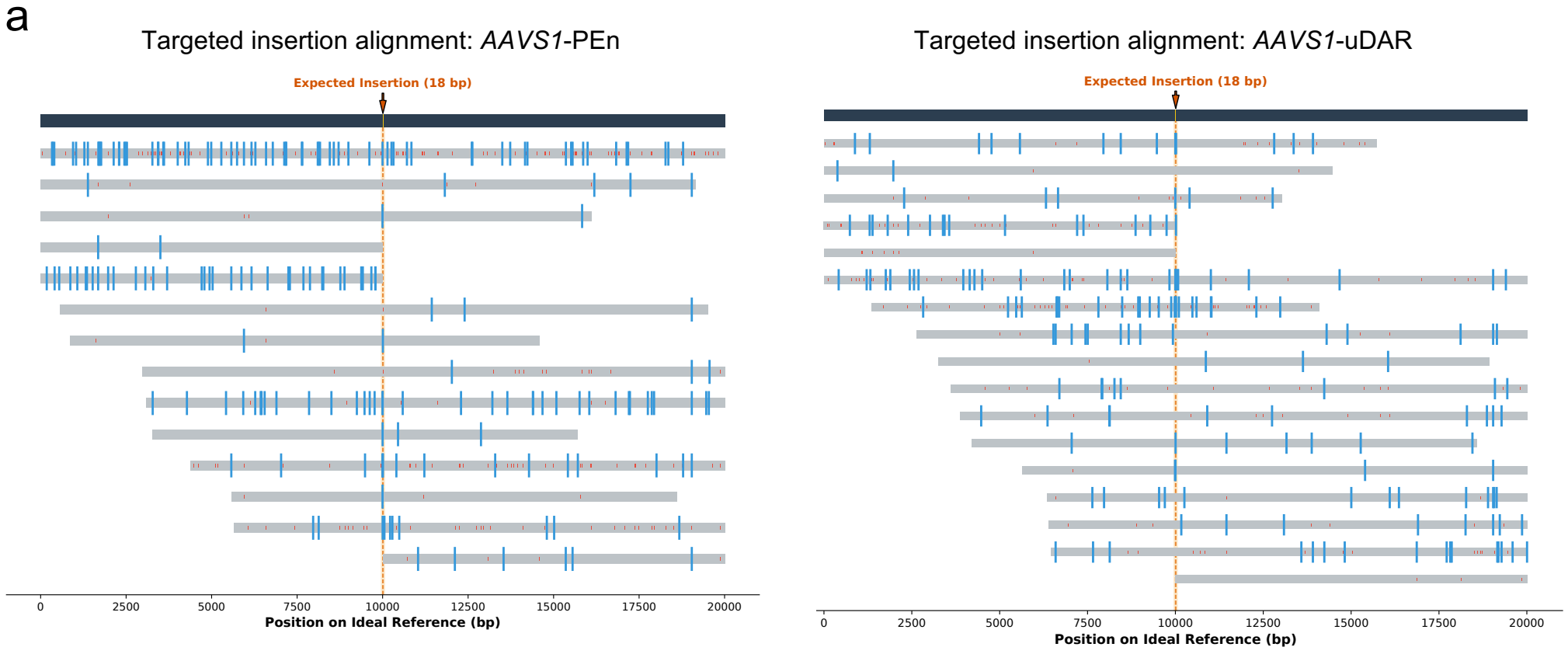

b

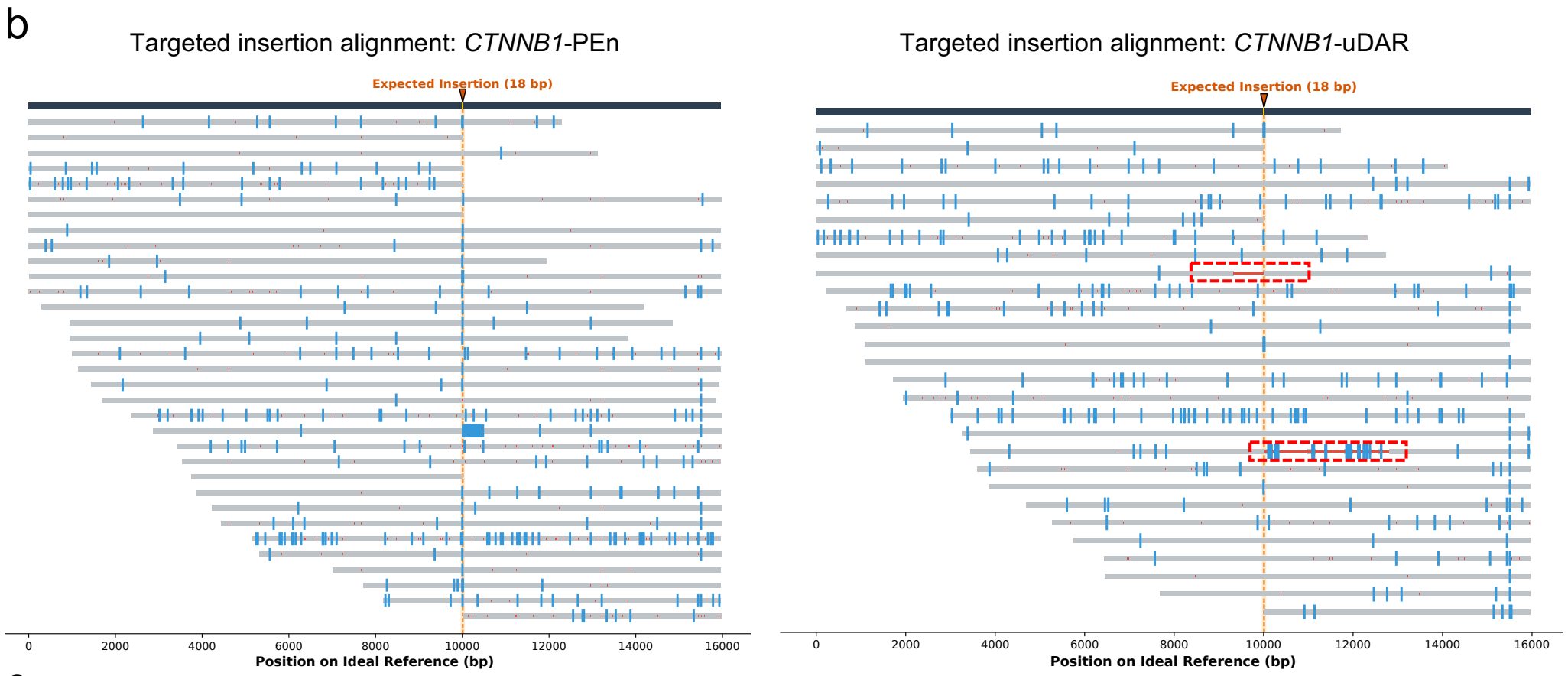

c

Sample	Sequenced reads	Mapping rate	Duplication	Mean depth (X)
AAVS1-PEn	7691011	100.00%	0.00%	38.9
AAVS1-uDAR	8235316	100.00%	0.00%	42.57
CTNNB1-PEn	7211528	100.00%	0.00%	37.68
CTNNB1-uDAR	8655211	100.00%	0.00%	44.06

d

	AAVS1-PEn	*AAVS1*-uDAR	*CTNNB1*-PEn	*CTNNB1*-uDAR
DEL	548	587	621	601
INS	1120	1149	1175	1143
DUP	77	76	72	73
INV	25	24	23	23
BND	114	118	96	116
Total_SVs	1884	1954	1987	1956

Supplementary Figure 15. Assessment of off-target large-fragment deletions mediated by PEn or uDAR at the *AAVS1* and *CTNNB1* sites in HEK293T cells.

a, b. The off-target large-fragment deletions analysis of PEn and uDAR at *AAVS1* (a) and *CTNNB1* (b) sites. The expected insertion has been clearly marked. Black lines indicate the ideal editing outcome; gray boxes indicate the matched region; red lines indicate the deletion sequences; blue lines indicate insertion sequences (Most of these

are systematic single base errors introduced by the sequencer). The red dashed boxes mark the off-target large-fragment deletions.

c. The information of four samples for PCR-free long-read sequencing. HEK293T cells are respectively transfected with PEn and uDAR systems for target fragment deletion at *AAVS1* and *CTNNB1* loci, followed by PCR-free long-read sequencing of the extracted genomic DNA.

d. Summary of total unique structural variants for each sample detected by PCR-free long-read sequencing in PEn and uDAR groups. Structural variants (SVs) include deletions (DEL), insertions (INS), duplications (DUP), inversions (INV) and translocations (BND).

Supplementary Tables

Supplementary Table S1. Sequences of pegF and pegR used in HEK293T and HeLa cells.

Sites	Spacer sequence of pegF/pegR	3' extension sequence of pegF/pegR	Intended edits
Reporter	GAAGTTCGAGGGCGACACCC	CTCGATGCGGTTCACCAGGGTGTCGCCCTCGAA	743-bp del/16-bp ins
	CGATGCCCTTCAGCTCGATG	GACACCCTGGTGAACCGCATCGAGCTGAAGGGC	
HEK3	GGCCCAGACTGAGCACGTGA	ATTACCCTGTTATCCCTACGTGCTCAGTCTG	991-bp del/18-bp ins
	TGATCACCTGCCCAAATGTG	TAGGGATAACAGGGTAATATTTGGGCAGGTG	
AAVS1	CCTCATCTGGCGATTTCCAC	ATTACCCTGTTATCCCTAGAAATCGCCAGAT	864-bp del/18-bp ins
	GGGGAATGGTAAGGAGGCCT	TAGGGATAACAGGGTAATCCTCCTTACCATT	
CTNNB1	CAAGATGATGGTCTGCCAAG	ATTACCCTGTTATCCCTAGGCAGACCATCAT	838-bp del/18-bp ins
	CCATGTGAAGGACGTGTCCA	TAGGGATAACAGGGTAATACACGTCCTTCAC	
RUNX1	ACTTTGGCAACAGACCAACT	ATTACCCTGTTATCCCTATGGTCTGTTGCCA	889-bp del/18-bp ins
	GGAGGAGCCGTACAGACAGA	TAGGGATAACAGGGTAATGTCTGTACGGCTC	
SEC61B	TATCCCAGGTACCCACCATA	ATTACCCTGTTATCCCTAGGTGGGTACCTGG	890-bp del/18-bp ins
	CACCTCATCAAGCATGGAGA	TAGGGATAACAGGGTAATCCATGCTTGATGA	
EMX1	CGGAGGTAGATTCCCAGGCA	ATTACCCTGTTATCCCTACTGGGAATCTACC	905-bp del/18-bp ins
	AGCCATAGACCCTTCTCACA	TAGGGATAACAGGGTAATGAGAAGGGTCTAT	
VISTA	ATGTGACCTTCTACAAGACG	ATTACCCTGTTATCCCTACTTGTAGAAGGTC	728-bp del/18-bp ins
	CACACCTCTATCCGAACCAT	TAGGGATAACAGGGTAATGTTCGGATAGAGG	
HNRNPK	ATTCAACTCTGCTTCAGTTC	ATTACCCTGTTATCCCTACTGAAGCAGAGTT	933-bp del/18-bp ins
	TCCACAGCATCAGATTCGAG	TAGGGATAACAGGGTAATGAATCTGATGCTG	
RNF43	CTGCACGGACATCAGCCTAC	ATTACCCTGTTATCCCTAGGCTGATGTCCGT	812-bp del/18-bp ins
	TGGCAGTGCGGATAAGGGCA	TAGGGATAACAGGGTAATCCTTATCCGCACT	
KCNA1	AGGAGGCCATGGAGAAGTTC	ATTACCCTGTTATCCCTACTTCTCCATGGCC	697-bp del/18-bp ins
	CACCGCCCACCAGAAAGCAT	TAGGGATAACAGGGTAATCTTTCTGGTGGGC	
LINC01948	CGGAGCCAGGCGAGTTCACA	AGAGCGGAGGCAGCACTGTGAACTCGCCTGGC	24-kb del/19-bp ins
	TCCTCAGATGTCTGGGAAGA	ACAGTGCTGCCTCCGCTCTTCCCAGACATCTG	
LINC01525	AAGAGTTCTGTTGGAACTCT	AAGACCCAAAGACCGCTAGAGTTCCAACAGAAC	26-kb del/7-bp ins
	AATGAAGACCCAAAGACCCA	GTTCTGTTGGAACTCTAGCGGTCTTTGGGTCTT	
SEC61B	TATCCCAGGTACCCACCATA	TCGTCGTCATCCTTGTAATCATTACCCTGTTATCCCTAGGTGGGTACCTGG	890-bp del/44-bp ins
	CACCTCATCAAGCATGGAGA	GATTACAAGGATGACGACGATAAGTGCCATGCTTGATGA	
CTNNB1	CAAGATGATGGTCTGCCAAG	TACTGCTCAAGAGCCCGGCAACGGATTACCCTGTTATCCCTAGGCAGACCATCAT	838-bp del/64-bp ins
	CCATGTGAAGGACGTGTCCA	TGCCGGGCTCTTGAGCAGTAGAGACTTTCTGGGGGGTCTCGCACACGTCCTTCAC	
AAVS1	CCTCATCTGGCGATTTCCAC	CCTCGAGCTTGTCATCGTCGTCCTTGTAATCATTACCCTGTTATCCCTAGAAATCGCCAGAT	864-bp del/78-bp ins

	GGGGAATGGTAAGGAGGCCT	CGACGATGACAAGCTCGAGGATGGGCGAGGGCGAT GCCACCTACGGCAACCTCCTTACCATT	
PDCD1	TCCCCTCTTTGCTCCTGGGC	ATTACCCTGTTATCCCTACAGGAGCAAAGAG	8-kb del/18-bp ins
	CTGGGCATGTGTAAAGGTGG	TAGGGATAACAGGGTAATCCTTTACACATGC	
PDCD1	TCCCCTCTTTGCTCCTGGGC	ATTACCCTGTTATCCCTACAGGAGCAAAGAG	10-kb del/18-bp ins
	CATTTTCCATGGGACCTATC	TAGGGATAACAGGGTAATAGGTCCCATGGAA	
PDCD1	TCCCCTCTTTGCTCCTGGGC	ATTACCCTGTTATCCCTACAGGAGCAAAGAG	12-kb del/18-bp ins
	TTCCCCTGAGCTCATAAAGC	TAGGGATAACAGGGTAATTTATGAGCTCAGG	
MECP2	AGGCCCTCACTCCTGTTCTA	ATTACCCTGTTATCCCTAAACAGGAGTGAGG	8-kb del/18-bp ins
	AGAGAAGGCAAGAAAGGAGA	TAGGGATAACAGGGTAATCCTTTCTTGCCTT	
MECP2	AGGCCCTCACTCCTGTTCTA	ATTACCCTGTTATCCCTAAACAGGAGTGAGG	10-kb del/18-bp ins
	AGTGTAAAGTCCTGGCCCTC	TAGGGATAACAGGGTAATGGCCAGGACTTTA	
MECP2	AGGCCCTCACTCCTGTTCTA	ATTACCCTGTTATCCCTAAACAGGAGTGAGG	12-kb del/18-bp ins
	ACACAAGGAAAGGGCTGAGA	TAGGGATAACAGGGTAATCAGCCCTTTCCTT	
DMD	TGCTTTCCCACACTCTCACT	ATTACCCTGTTATCCCTAGAGAGTGTGGGAA	25-kb del/18-bp ins
	CGCTTAGCTAAATCCTTAGA	TAGGGATAACAGGGTAATAAGGATTTAGCTA	
DMD	TGCTTTCCCACACTCTCACT	ATTACCCTGTTATCCCTAGAGAGTGTGGGAA	50-kb del/18-bp ins
	TCTCCTCCTTCCTCTCATCT	TAGGGATAACAGGGTAATTGAGAGGAAGGAG	
DMD	TGCTTTCCCACACTCTCACT	ATTACCCTGTTATCCCTAGAGAGTGTGGGAA	75-kb del/18-bp ins
	TCCGCCAGGAATGTTTTCAG	TAGGGATAACAGGGTAATAAAACATTCCTGG	
DMD	TGCTTTCCCACACTCTCACT	ATTACCCTGTTATCCCTAGAGAGTGTGGGAA	100-kb del/18-bp ins
	GCCTAGCCAGGTATTAAATT	TAGGGATAACAGGGTAATTTAATACCTGGCT	
RUNX1	GCATTTTCAGGAGGAAGCGA	ATTACCCTGTTATCCCTACTTCCTCCTGAAA	25-kb del/18-bp ins
	TGCCTGATTTACAGGAGTGA	TAGGGATAACAGGGTAATCTCCTGTAAATCA	
RUNX1	GCATTTTCAGGAGGAAGCGA	ATTACCCTGTTATCCCTACTTCCTCCTGAAA	50-kb del/18-bp ins
	AGCCCAGTCCTCATCTCATC	TAGGGATAACAGGGTAATGAGATGAGGACTG	
RUNX1	GCATTTTCAGGAGGAAGCGA	ATTACCCTGTTATCCCTACTTCCTCCTGAAA	75-kb del/18-bp ins
	CTCAAATCCCTGCAACGTAC	TAGGGATAACAGGGTAATCGTTGCAGGGATT	
RUNX1	GCATTTTCAGGAGGAAGCGA	ATTACCCTGTTATCCCTACTTCCTCCTGAAA	100-kb del/18-bp ins
	CGTGGGATGTTGCTAACATT	TAGGGATAACAGGGTAATGTTAGCAACATCC	
SEC61B-uDAR	TATCCCAGGTACCCACCATA	CGTCGTCATCCTTGTAATCATTACAGGTGGGTACCTGG	890-bp del/32-bp ins
	CACCTCATCAAGCATGGAGA	GATTACAAGGATGACGACGATAAGTGCCATGCTTGATGA	
CTNNB1-uDAR	CAAGATGATGGTCTGCCAAG	ACTGCTCAAGAGCCCGGCAACGGTGTTATCCCTAGGCAGACCATCAT	838-bp del/49-bp ins
	CCATGTGAAGGACGTGTCCA	TGCCGGGCTCTTGAGCAGTAGAGACTTGTCTCGCACACGTCCTTCAC	
AAVS1-uDAR	CCTCATCTGGCGATTTCCAC	CCTCGAGCTTGTCATCGTCGTCCTTGTAATCATTACCCTGTTATCCCTAGAAATCGCCAGAT	864-bp del/62-bp ins
	GGGGAATGGTAAGGAGGCCT	CGACGATGACAAGCTCGAGGATGGGCGACCACCTACGGCAACCTCCTTACCAT	

SEC61B-TW	TATCCCAGGTACCCACCATA	ACTTATCGTCGTCATCCTTGTAATCATTACAGGTGGGTACCTGG	890-bp del/32-bp ins
	CACCTCATCAAGCATGGAGA	GTAATGATTACAAGGATGACGACGATAAGTGCCATGCTTGATGA	
CTNNB1-TW	CAAGATGATGGTCTGCCAAG	GCGAGACAAGTCTCTACTGCTCAAGAGCCCGGCAACGGTGTTATCCCTAGGCAGACCATCAT	838-bp del/49-bp ins
	CCATGTGAAGGACGTGTCCA	TAGGGATAACACCGTTGCCGGGCTCTTGAGCAGTAGAGACTTGTCTCGCACACGTCCTTCAC	
AAVS1-TW	CCTCATCTGGCGATTTCCAC	TTGCCGTAGGTGGTCGCCCATCCTCGAGCTTGTCATCGTCGTCCTTGTAATCGTTATCCCTAGAAATCGCCAGAT	864-bp del/62-bp ins
	GGGGAATGGTAAGGAGGCCT	TAGGGATAACGATTACAAGGACGACGATGACAAGCTCGAGGATGGGCGACCACCTACGGCAACCTCCTTACCAT	
SEC61B-PD	TATCCCAGGTACCCACCATA	TATCACCTCATCAAGCATGGCACTTATCGTCGTCATCCTTGTAATCATTACAGGTGGGTACCTGG	890-bp del/32-bp ins
	CACCTCATCAAGCATGGAGA	TCTATCCCAGGTACCCACCTGTAATGATTACAAGGATGACGACGATAAGTGCCATGCTTGATGA	
CTNNB1-PD	CAAGATGATGGTCTGCCAAG	TTGACCATGTGAAGGACGTGTGCGAGACAAGTCTCTACTGCTCAAGAGCCCGGCAACGGTGTTATCCCTAGGCAGACCATCAT	838-bp del/49-bp ins
	CCATGTGAAGGACGTGTCCA	AGAACAAGATGATGGTCTGCCTAGGGATAACACCGTTGCCGGGCTCTTGAGCAGTAGAGACTTGTCTCGCACACGTCCTTCAC	
AAVS1-PD	CCTCATCTGGCGATTTCCAC	AAGGGGAATGGTAAGGAGGTTGCCGTAGGTGGTCGCCCATCCTCGAGCTTGTCATCGTCGTCCTTGTAATCGTTATCCCTAGAAATCGCCAGAT	864-bp del/62-bp ins
	GGGGAATGGTAAGGAGGCCT	AGGCCCTCATCTGGCGATTTCTAGGGATAACGATTACAAGGACGACGATGACAAGCTCGAGGATGGGCGACCACCTACGGCAACCTCCTTACCAT	
SEC61B-HR5	TATCCCAGGTACCCACCATA	ATCCTTGTAATCATTACCCTGTTATCCCTAGGTGGGTACCTGG	890-bp del/54-bp ins
	CACCTCATCAAGCATGGAGA	AAGGATGACGACGATAAGTGATCTGGCGATCCATGCTTGATGA	
SEC61B-HR10	TATCCCAGGTACCCACCATA	TCATCCTTGTAATCATTACCCTGTTATCCCTAGGTGGGTACCTGG	890-bp del/54-bp ins
	CACCTCATCAAGCATGGAGA	TACAAGGATGACGACGATAAGTGATCTGGCGATCCATGCTTGATGA	
SEC61B-HR15	TATCCCAGGTACCCACCATA	TCGTCATCCTTGTAATCATTACCCTGTTATCCCTAGGTGGGTACCTGG	890-bp del/54-bp ins
	CACCTCATCAAGCATGGAGA	ATTACAAGGATGACGACGATAAGTGATCTGGCGATCCATGCTTGATGA	
SEC61B-HR20	TATCCCAGGTACCCACCATA	TCGTCGTCATCCTTGTAATCATTACCCTGTTATCCCTAGGTGGGTACCTGG	890-bp del/54-bp ins

	CACCTCATCAAGCATGGAGA	TGATTACAAGGATGACGACGATAAGTGATCTGGCGATCCATGCTTGATGA	
SEC61B-HR25	TATCCCAGGTACCCACCATA	TATCGTCGTCATCCTTGTAATCATTACCCTGTTATCCCTAGGTGGGTACCTGG	890-bp del/54-bp ins
	CACCTCATCAAGCATGGAGA	AATGATTACAAGGATGACGACGATAAGTGATCTGGCGATCCATGCTTGATGA	
SEC61B-HR30	TATCCCAGGTACCCACCATA	ACTTATCGTCGTCATCCTTGTAATCATTACCCTGTTATCCCTAGGTGGGTACCTGG	890-bp del/54-bp ins
	CACCTCATCAAGCATGGAGA	GTAATGATTACAAGGATGACGACGATAAGTGATCTGGCGATCCATGCTTGATGA	
SEC61B-HR40	TATCCCAGGTACCCACCATA	AGATCACTTATCGTCGTCATCCTTGTAATCATTACCCTGTTATCCCTAGGTGGGTACCTGG	890-bp del/54-bp ins
	CACCTCATCAAGCATGGAGA	ACAGGGTAATGATTACAAGGATGACGACGATAAGTGATCTGGCGATCCATGCTTGATGA	
SEC61B-TW	TATCCCAGGTACCCACCATA	ATCGCCAGATCACTTATCGTCGTCATCCTTGTAATCATTACCCTGTTATCCCTAGGTGGGTACCTGG	890-bp del/54-bp ins
	CACCTCATCAAGCATGGAGA	TAGGGATAACAGGGTAATGATTACAAGGATGACGACGATAAGTGATCTGGCGATCCATGCTTGATGA	
SEC61B-PD	TATCCCAGGTACCCACCATA	TATCACCTCATCAAGCATGGATCGCCAGATCACTTATCGTCGTCATCCTTGTAATCATTACCCTGTTATCCCTAGGTGGGTACCTGG	890-bp del/54-bp ins
	CACCTCATCAAGCATGGAGA	TTCTATCCCAGGTACCCACCTAGGGATAACAGGGTAATGATTACAAGGATGACGACGATAAGTGATCTGGCGATCCATGCTTGATGA	
RUNX1-HR5	ACTTTGGCAACAGACCAACT	TGTTATCCCTATGGTCTGTTGCCA	889-bp del/18-bp ins
	GGAGGAGCCGTACAGACAGA	TAACAGGGTAATGTCTGTACGGCTC	
RUNX1-HR10	ACTTTGGCAACAGACCAACT	CCTGTTATCCCTATGGTCTGTTGCCA	889-bp del/18-bp ins
	GGAGGAGCCGTACAGACAGA	GGATAACAGGGTAATGTCTGTACGGCTC	
RUNX1-HR15	ACTTTGGCAACAGACCAACT	TACCCTGTTATCCCTATGGTCTGTTGCCA	889-bp del/18-bp ins
	GGAGGAGCCGTACAGACAGA	AGGGATAACAGGGTAATGTCTGTACGGCTC	
RUNX1-HR20	ACTTTGGCAACAGACCAACT	ACATTACCCTGTTATCCCTATGGTCTGTTGCCA	889-bp del/18-bp ins
	GGAGGAGCCGTACAGACAGA	TAGGGATAACAGGGTAATGTCTGTACGGCTC	
RUNX1-HR25	ACTTTGGCAACAGACCAACT	AGACATTACCCTGTTATCCCTATGGTCTGTTGCCA	889-bp del/18-bp ins
	GGAGGAGCCGTACAGACAGA	CCATAGGGATAACAGGGTAATGTCTGTACGGCTC	
RUNX1-HR30	ACTTTGGCAACAGACCAACT	ACAGACATTACCCTGTTATCCCTATGGTCTGTTGCCA	889-bp del/18-bp ins
	GGAGGAGCCGTACAGACAGA	AGACCATAGGGATAACAGGGTAATGTCTGTACGGCTC	
RUNX1-HR40	ACTTTGGCAACAGACCAACT	AGCCGTACAGACATTACCCTGTTATCCCTATGGTCTGTTGCCA	889-bp del/18-bp ins
	GGAGGAGCCGTACAGACAGA	CAACAGACCATAGGGATAACAGGGTAATGTCTGTACGGCTC	
RUNX1-TW	ACTTTGGCAACAGACCAACT	ATTACCCTGTTATCCCTATGGTCTGTTGCCA	889-bp del/18-bp ins
	GGAGGAGCCGTACAGACAGA	TAGGGATAACAGGGTAATGTCTGTACGGCTC	

RUNX1-PD	ACTTTGGCAACAGACCAACT	GCTGGAGGAGCCGTACAGACATTACCCTGTTATCCCTATGGTCTGTTGCCA	889-bp del/18-bp ins
	GGAGGAGCCGTACAGACAGA	TGGACTTTGGCAACAGACCATAGGGATAACAGGGTAATGTCTGTACGGCTC	
AAVS1-HR5	CCTCATCTGGCGATTTCCAC	TTACCCTGTTATCCCTAGAAATCGCCAGAT	864-bp del/32-bp ins
	GGGGAATGGTAAGGAGGCCT	GGTAATGATTACAACTACGGCCTCCTTACCAT	
AAVS1-HR10	CCTCATCTGGCGATTTCCAC	TCATTACCCTGTTATCCCTAGAAATCGCCAGAT	864-bp del/32-bp ins
	GGGGAATGGTAAGGAGGCCT	AGGGTAATGATTACAACTACGGCCTCCTTACCAT	
AAVS1-HR15	CCTCATCTGGCGATTTCCAC	TAATCATTACCCTGTTATCCCTAGAAATCGCCAGAT	864-bp del/32-bp ins
	GGGGAATGGTAAGGAGGCCT	ACAGGGTAATGATTACAACTACGGCCTCCTTACCAT	
AAVS1-HR20	CCTCATCTGGCGATTTCCAC	TTGTAATCATTACCCTGTTATCCCTAGAAATCGCCAGAT	864-bp del/32-bp ins
	GGGGAATGGTAAGGAGGCCT	TAACAGGGTAATGATTACAACTACGGCCTCCTTACCAT	
AAVS1-HR25	CCTCATCTGGCGATTTCCAC	AGTTGTAATCATTACCCTGTTATCCCTAGAAATCGCCAGAT	864-bp del/32-bp ins
	GGGGAATGGTAAGGAGGCCT	GGATAACAGGGTAATGATTACAACTACGGCCTCCTTACCAT	
AAVS1-HR30	CCTCATCTGGCGATTTCCAC	CGTAGTTGTAATCATTACCCTGTTATCCCTAGAAATCGCCAGAT	864-bp del/32-bp ins
	GGGGAATGGTAAGGAGGCCT	AGGGATAACAGGGTAATGATTACAACTACGGCCTCCTTACCAT	
AAVS1-HR40	CCTCATCTGGCGATTTCCAC	GAGGCCGTAGTTGTAATCATTACCCTGTTATCCCTAGAAATCGCCAGAT	864-bp del/32-bp ins
	GGGGAATGGTAAGGAGGCCT	TTTCTAGGGATAACAGGGTAATGATTACAACTACGGCCTCCTTACCAT	
AAVS1-TW	CCTCATCTGGCGATTTCCAC	CCGTAGTTGTAATCATTACCCTGTTATCCCTAGAAATCGCCAGAT	864-bp del/32-bp ins
	GGGGAATGGTAAGGAGGCCT	TAGGGATAACAGGGTAATGATTACAACTACGGCCTCCTTACCAT	
AAVS1-PD	CCTCATCTGGCGATTTCCAC	GAAGGGGAATGGTAAGGAGGCCGTAGTTGTAATCATTACCCTGTTATCCCTAGAAATCGCCAGAT	864-bp del/32-bp ins
	GGGGAATGGTAAGGAGGCCT	GGCCCTCATCTGGCGATTTCTAGGGATAACAGGGTAATGATTACAACTACGGCCTCCTTACCAT	
HEK3-HR5	GGCCCAGACTGAGCACGTGA	TCATCCTTGTAATCCGTGCTCAGTCTG	991-bp del/24-bp ins
	TGATCACCTGCCCAAATGTG	GATGACGACGATAAGATTTGGGCAGGTG	
HEK3-HR10	GGCCCAGACTGAGCACGTGA	TCGTCATCCTTGTAATCCGTGCTCAGTCTG	991-bp del/24-bp ins
	TGATCACCTGCCCAAATGTG	AGGATGACGACGATAAGATTTGGGCAGGTG	
HEK3-HR15	GGCCCAGACTGAGCACGTGA	TCGTCGTCATCCTTGTAATCCGTGCTCAGTCTG	991-bp del/24-bp ins
	TGATCACCTGCCCAAATGTG	CAAGGATGACGACGATAAGATTTGGGCAGGTG	
HEK3-HR20	GGCCCAGACTGAGCACGTGA	TATCGTCGTCATCCTTGTAATCCGTGCTCAGTCTG	991-bp del/24-bp ins
	TGATCACCTGCCCAAATGTG	TTACAAGGATGACGACGATAAGATTTGGGCAGGTG	

HEK3-HR25	GGCCCAGACTGAGCACGTGA	TCTTATCGTCGTCATCCTTGTAATCCGTGCTCAGTCTG	991-bp del/24-bp ins
	TGATCACCTGCCCAAATGTG	GATTACAAGGATGACGACGATAAGATTTGGGCAGGTG	
HEK3-HR30	GGCCCAGACTGAGCACGTGA	AATCTTATCGTCGTCATCCTTGTAATCCGTGCTCAGTCTG	991-bp del/24-bp ins
	TGATCACCTGCCCAAATGTG	ACGGATTACAAGGATGACGACGATAAGATTTGGGCAGGTG	
HEK3-HR40	GGCCCAGACTGAGCACGTGA	GCCCAAATCTTATCGTCGTCATCCTTGTAATCCGTGCTCAGTCTG	991-bp del/24-bp ins
	TGATCACCTGCCCAAATGTG	TGAGCACGGATTACAAGGATGACGACGATAAGATTTGGGCAGGTG	
HEK3-TW	GGCCCAGACTGAGCACGTGA	CTTATCGTCGTCATCCTTGTAATCCGTGCTCAGTCTG	991-bp del/24-bp ins
	TGATCACCTGCCCAAATGTG	GATTACAAGGATGACGACGATAAGATTTGGGCAGGTG	
HEK3-PD	GGCCCAGACTGAGCACGTGA	CATTGATCACCTGCCCAAATCTTATCGTCGTCATCCTTGTAATCCGTGCTCAGTCTG	991-bp del/24-bp ins
	TGATCACCTGCCCAAATGTG	TGGGGCCCAGACTGAGCACGGATTACAAGGATGACGACGATAAGATTTGGGCAGGTG	

Note: The PBS sequence are shown in blue. The RT templates are shown in orange.

Supplementary Table S2. Sequences of pegF and pegR used in N2a and hESC cells.

Sites	Spacer sequence of pegF/pegR	3' extension sequence of pegF/pegR	Intended edits
Tspan2	CTACACCTGCTCATCCAACA	ATTACCCTGTTATCCCTATGGATGAGCAGGT	1064-bp del/18-bp ins
	GAGTTAAACCCACCACCCAC	TAGGGATAACAGGGTAATGGTGGTGGGTTTA	
Col12a1	CTCAATCACCCAAGCAGCGT	ATTACCCTGTTATCCCTACTGCTTGGGTGAT	699-bp del/18-bp ins
	GTTGCCATCTACATCACTGT	TAGGGATAACAGGGTAATGTGATGTAGATGG	
Casp1	ATAGAGCCTTACTGAACTAT	ATTACCCTGTTATCCCTAGTTCAGTAAGGCT	991-bp del/18-bp ins
	TCTAGCCTTTCCACTCTAGG	TAGGGATAACAGGGTAATAGAGTGGAAAGGC	
Angpt1	AGGACACAGGTTGAGGGACC	ATTACCCTGTTATCCCTACCCTCAACCTGTG	1124-bp del/18-bp ins
	CAAGAGCTGTCTGGTTCCAG	TAGGGATAACAGGGTAATGAACCAGACAGCT	
AGXT-hESC	GTTCCCATCCAGCTGCAGCC	CCTGAGTGCAGTTCTCACTGCAGCTGGATGG	733-bp del/6-bp ins
	GAATCCCTGAGTGCAGTGAG	CATCCAGCTGCAGTGAGAACTGCACTCAGGG	
MECP2-hESC	AAAGGTAGGCGACACATCCC	GGTACGCAGGTCGAGGGATGTGTCGCCTAC	721-bp del/17-bp ins
	TCTCGGGCTCAGGTGGAGGT	CCCTCGACCTGCGTACCTCCACCTGAGCCC	

Note: The PBS sequence are shown in blue. The RT templates are shown in orange.

Supplementary Table S3. Primers for plasmids construction

Description	Sequence
Backbone pegRNA-F	AGCTAGGTCTCCTTTTTTTAAAGAATTCTCGACCTCGAGAC
Backbone pegRNA-R	TCTCTCGGTCTCACGGTGTTTCGT
sgRNA scaffold-top	AGAGCTAGAAATAGCAAGTTAAAATAAGGCTAGTCCGTTATCAACTTGAAAAAGTGGCACCGAGTCG
sgRNA scaffold-bottom	GCACCGACTCGGTGCCACTTTTTCAAGTTGATAACGGACTAGCCTTATTTTAACTTGCTATTTCTAG

Supplementary Table S4. Primers used for genomic DNA amplification.

Description	Forward Primer (5'-3')	Reverse Primer (5'-3')
HEK3	TAAGCAAGGGCTGATGTGGG	TGTGGTGCCTTTTCGGCTTA
AAVS1	CAACTCTGCCTCTCCCCGCA	GAGGGCAGCGCAAAGTGACA
CTNNB1	CTGGTGCCATGGGAATAGAG	ATGCATGATGAGGCAAGGGT
RUNX1	GATGCCTGGAGTATGATTTGGT	AAGGGTGAGTCTCAGAACAG
SEC61B	CTTTCTCCCTCATCTCAAGG	TTAGGAGAGCAAGAACACCA
EMX1	TTCCAGGGTGAGTGTCCACG	TGCCTGACAAATCGGGACCT
VISTA	GCTCTGGGGTTGCCAGGAGT	CCCCAGGTTCTCACGCTAGT
Tspan2	TACTGCTCCTGTCAACACAG	TTCAGTGCACGGACACAATG
Col12a1	ACTGTGGGTCTCTATCTGTT	ACATCTCCTCCTGGATAAGG
Casp1	TATCCTTAAGGCCTTGCATC	CAAGTCACAAGACCAGGCAT
Angpt1	CATCCTCACTGAAGTGTAAC	TCCAGAGCTCACTATTGTCA
HNRNPK	TCTTGCAGAGCCCATTAGTG	CATGGAGCAGGGTCAACAGT
RNF43	CCACCTCACAGCACCCTGCT	CCAGCATGATACGCTGTCCC
KCNA1	CAGTTCCCCAACACGCTGCT	ACAATGACAGGTACGGGCAG
LINC01948	CTGAAGATCTGCCTGGACCC	GAGACTTGTCATAGTCCCAG
LINC01525	AGTTCACTGGAATCCCTGGT	ACCCATCATAAGGGTGATGC
PDCD1-del8k	GGTTCTTAGGTAGGTGGGGT	CCCTAGGTGCCTGCACTTCT
PDCD1-del10k	GGTTCTTAGGTAGGTGGGGT	AGTGGGTGTTTGAGAGCTGT
PDCD1-del12k	GGTTCTTAGGTAGGTGGGGT	ATCTCCTGACCTTGTGATCCA
MECP2-del8k	CCAGGTAAACTTCTTGCCGC	GATAACAGCACAGCACAGCA
MECP2-del10k	CCAGGTAAACTTCTTGCCGC	AGATCATGTTCAGCGGATGC
MECP2-del12k	CCAGGTAAACTTCTTGCCGC	ACAAAGGACGGGGTGGAACT
AGXT-hESC	CAACGTGGGCCACACACAGT	CCAGGGTGGGGCATTTGACT
MECP2-hESC	TCCGCTCTGCCCTATCTCTG	GAGCCTCCTCTGGGCATCTT
AAVS1-cutF	CAACTCTGCCTCTCCCCGCA	CGCTGGGAAATGGAGTCCAT
AAVS1-cutR	GAGGGCAGCGCAAAGTGACA	CGACCACCCCACTTCCGAAT
DMD-del25k	CAACTCCCGCTGGAAATCAT	ACTGCATCCAAGCTTGTTGT
DMD-del50k	CAACTCCCGCTGGAAATCAT	TTACAGGCGTGCACTACCAT
DMD-del75k	CAACTCCCGCTGGAAATCAT	TTTCCTCTGTGAGAAGCCAT
DMD-del100k	CAACTCCCGCTGGAAATCAT	ACCGAGGCTTTGCCCTATAT
RUNX1-del25k	GGTGCATTTTTTAATAGGGCT	GGATGACTGTCTCCTCCCTA

RUNX1-del50k	GGTGCATTTTTTAATAGGGCT	ATCAGCCCCATTTTCCTGAG
RUNX1-del75k	GGTGCATTTTTTAATAGGGCT	CCATCTGTCTCTTAGAGACT
RUNX1-del100k	GGTGCATTTTTTAATAGGGCT	GTTCATAAACATGTCCCATG

Supplementary Table S5. Primers for qPCR.

Description	Sequence
HEK3-P1	AAGTGGCCTTCTAGAGCTGG
HEK3-P2	CTAGCTAGAGTGCTTGGGGC
HEK3-P3	TTTTCTGCTTCTCCAGCCCTG
HEK3-P4	CTGTGAACCTGTATAGGCGACT
HEK3-P5	CTGAGCACGTAGGGATAACAGG
HEK3-P6	GGGCTTCGGATACAAGAGGAC
AAVS1-P1	TCCACCAACGCCGACGGTAT
AAVS1-P2	ACGGGGTGTCAGCATAGGGT
AAVS1-P3	CTCCGGCCTGTAGACTCCAT
AAVS1-P4	GAAGTCGAGGGAGGGATGGT
AAVS1-P5	GGCGATTTCTAGGGATAACAGGG
AAVS1-P6	GACAAAGTCCAGGACCGGCT

Supplementary Table S6. Primers used for on/off-targeted deep sequencing.

Description	Forward Primer (5'-3')	Reverse Primer (5'-3')
293T-HEK3	NNNNNCTGCTGCAAGTAAGCATGCA	GGGCTTCGGATACAAGAGGAC
293T-hAAVS1	NNNNNCTCCGGCCTGTAGACTCCAT	GAAGTCGAGGGAGGGATGGT
293T-CTNNB1	NNNNNCATAGGAAGGGATGGAAGGT	CTAAGGAAGTGGGCCTTTGT
293T-RUNX1	NNNNNACCTACCAGACATCATGGAT	AAGGGTGAGTCTCAGAACAG
293T-SEC61B	NNNNNGTCTGTAAGCAGCAGTAGCT	TTAGGAGAGCAAGAACACCA
293T-EMX1	NNNNNTTCCAGGGTGAGTGTCCACG	CCGTCACATTAGCTCCTGGA
293T-VISTA	NNNNNCAAGGTCGCCACGCCGTATT	CCTGCACACGTGTGCACCCA
HeLa-HNRNPK	NNNNNCTCCAGATACAATGTTCACC	TCGATCCTATGGGCCAGGCT
HeLa -KCNA1	NNNNNAGCTTCGACGCCATCCTCTA	GCACACAAGGAGCCCACGAT
HeLa -RNF43	NNNNNCCCCTGACAGCAGTGGATCT	AGCTGTAGTCTCCTCTCCCT
HeLa -SEC61B	NNNNNGTCTGTAAGCAGCAGTAGCT	TTAGGAGAGCAAGAACACCA
HeLa -EMX1	NNNNNTTCCAGGGTGAGTGTCCACG	CCGTCACATTAGCTCCTGGA
HeLa -VISTA	NNNNNCAAGGTCGCCACGCCGTATT	CCTGCACACGTGTGCACCCA
N2a-Tspan2	NNNNNAGCAGAGCTGGGTCTCCAGT	ATGCCAGGGAACAGAAGGCT
N2a-Col12a1	NNNNNGGCACAGATCTATGTGTATAG	GATAGACTATACTACAGCACT
N2a-Casp1	NNNNNAGCCTACACCTATATTGGGT	AGGATGTCTCCAAGACACAT

N2a-Angpt1	NNNNNATAGAATGAGTCCTCAGACT	TCTACTTCTAAGTGTCTGCT
MECP2-8Kdel	NNNNNCAGAGAGCACAGAAGCAAGCT	ATGCCTGGCCTCCAATTTGT
MECP2-10Kdel	NNNNNCAGAGAGCACAGAAGCAAGCT	GGGCACTTGAGCCTAGCTGA
MECP2-12Kdel	NNNNNCAGAGAGCACAGAAGCAAGCT	CTCCCATCAACTCGTGAGGT
PDCD1-8Kdel	NNNNNCTTACCCTGAGCTGTGTGGCT	GTGAGGGGCTGGGGTGGAAT
PDCD1-10Kdel	NNNNNCTTACCCTGAGCTGTGTGGCT	AGTGGGTGTTTGAGAGCTGT
PDCD1-12Kdel	NNNNNCTTACCCTGAGCTGTGTGGCT	AGATGGCTATCCCTATCCCT
LINC01948	NNNNNCTGAAGATCTGCCTGGACCC	GAGACTTGTCATAGTCCCAG
LINC01525	NNNNNCCTGGTGTCATTCAGTGCCT	GGGTGATGCCTGATACTTCT
CTNNB1-64bp-ins	NNNNNTCAGCTTCTGGGTTCAGATG	CTAAGGAAGTGGGCCTTTGT
SEC61B-44bp-ins	NNNNNCCCCATAGCATTTTGTTCAT	TTAGGAGAGCAAGAACACCA
AAVS1-78bp-ins	NNNNNCACACTTCCTAGTATGCCGTG	CCAAAGCCAGGGCCAGTTAAAG
hESC-MECP2	NNNNNTCCGCTCTGCCCTATCTCTG	GAGCCTCCTCTGGGCATCTT
hESC-AGXT	NNNNNCAACGTGGGCCACACACAGT	CCAGGGTGGGGCATTTGACT
293T-HEK3(F2/R2)	NNNNNAGGGAAACGCCCATGCAATT	CCTGCCAGTCAGTTCTGTGA
293T-hAAVS1(F2/R2)	NNNNNCACACTTCCTAGTATGCCGT	AACATCGGAAGAGGGGAAGT
293T-CTNNB1(F2/R2)	NNNNNTGGAAGGTCTCCTTGGGACT	CTAAGGAAGTGGGCCTTTGT
293T-RUNX1(F2/R2)	NNNNNCCAGACATCATGGATGAGAG	AAGCCTGCAAGGGTGAGTCT
HEK3-pegF-OT1	TGACTCAGAAATTGTCAGCGA	TCGCTGACAATTTCTGAGTCA
HEK3-pegF-OT2	TTGGGCCTTTGGACCAGGAATCT	AGATTCCTGGTCCAAAGGCCCAA
HEK3-pegF-OT3	AGTCACTTAGGGTTGGATCCGTG	CACGGATCCAACCCTAAGTGACT
HEK3-pegF-OT4	ACTACACCTGCAGCCCACCTA	TAGGTGGGCTGCAGGTGTAGT
HEK3-pegF-OT5	ACCTTCCACGAAACCAGTGC	GCACTGGTTTCGTGGAAGGT
HEK3-pegF-OT6	AAAGCTGTGGGGAGGGAGGAAGA	TCTTCCTCCCTCCCCACAGCTTT
HEK3-pegR-OT1	ATGCCTGGGTCAGAGACAAGGGA	TCCCTTGTCTCTGACCCAGGCAT
HEK3-pegR-OT2	TATCTTTGGTTCTCAAAGGT	ACCTTTGAGAACCAAAGATA
HEK3-pegR-OT3	CCTGATAAAGCAAACACAGA	TCTGTGTTTGCTTTATCAGG
HEK3-pegR-OT4	AGGACGGCACTAGGACTAGCTG	CAGCTAGTCCTAGTGCCGTCCT
HEK3-pegR-OT5	TGCACTTACCAGAGCAATTC	GAATTGCTCTGGTAAGTGCA
HEK3-pegR-OT6	TGAGCCCCTTGGCTGATTAA	TTAATCAGCCAAGGGGCTCA
RUNX1-pegF-OT1	AGTGGACTGAGGATGATGATG	CATCATCATCCTCAGTCCACT
RUNX1-pegF-OT2	TAAGGAATCACAGCAGATGGC	GCCATCTGCTGTGATTCCTTA
RUNX1-pegF-OT3	TGAGAATGATGATACCTACCT	AGGTAGGTATCATCATTCTCA
RUNX1-pegF-OT4	TGCACCTTCTTAGTTTGTGGG	CCCACAAACTAAGAAGGTGCA
RUNX1-pegF-OT5	AACCTGGGGATCATCCTGGTA	TACCAGGATGATCCCCAGGTT
RUNX1-pegF-OT6	AGATACTAACATTGCTTGTCT	AGACAAGCAATGTTAGTATCT
RUNX1-pegR-OT1	AATAGCTAGAAGAGTGGGAAG	CTTCCCACTCTTCTAGCTATT
RUNX1-pegR-OT2	TGCTTTCGTCGGAGACGTAGCCT	AGGCTACGTCTCCGACGAAAGCA
RUNX1-pegR-OT3	AGATCATGGGATCACGTTAGACA	TGTCTAACGTGATCCCATGATCT
RUNX1-pegR-OT4	CTGCGGGGAGATGAGCTCAA	TTGAGCTCATCTCCCCGCAG
RUNX1-pegR-OT5	ATCTCATCTCCAGTTGTAATC	GATTACAACTGGAGATGAGAT
RUNX1-pegR-OT6	AGAGATGTGATCACAGCATGG	CCATGCTGTGATCACATCTCT
CTNNB1-pegF-OT1	CTAACAGTCTGACACTAACC	AAAGACATTGTGCTCCATTG

CTNNB1-pegF-OT2	CTGTTTAAATGATGGTGAGCA	AGTGAGTTCAGTCTGACTGC
CTNNB1-pegF-OT3	AGACTTCAGGGACTCTAAAT	GTCTGCTGCATCTATTAATC
CTNNB1-pegF-OT4	AGACACACACACCGGATGT	CACGGTGGGTTTCTGAGCCT
CTNNB1-pegF-OT5	GTTAACATAA ATTAGAAAGTTGGAG	ACATGGACCATTCTCTCTCT
CTNNB1-pegF-OT6	TGGCTGATCGTGGTGCTTAC	TGTTGGTGGATGTGGGAGTA
CTNNB1-pegR-OT1	AATGTTGAAGAGGAGAGAGC	TGCTGTAGGTATTAGGAATGC
CTNNB1-pegR-OT2	CATCCTTGTCATGGCCTCAT	CTCTACAGATCAGCTGGCAG
CTNNB1-pegR-OT3	CACTTACCAGATGAAAATGA	GAAAGGTTAATGGATCCAAG
CTNNB1-pegR-OT4	CACCAACAATAAGCAAGCAGAGA	CATGATATTGATTCTTCCTATCCAT
CTNNB1-pegR-OT5	TTAAGTCATGAGGGCACAGC	TTTCTCACAGTTCAGGAGGA
CTNNB1-pegR-OT6	CAGCCTGGCCTTAAAGGACATTACCA	TCAAAACTCTTTCCTTCACG
SEC61B-pegF-OT1	TCAACCCCACAGCCACCCAG	CTGCCATCCACAAAACCAAG
SEC61B-pegF-OT2	CCTTACCCTTCAAATTGTCT	CATGGTTTCCCCTACCCTCA
SEC61B-pegF-OT3	ACCTGGCCTGTCACTCAGCA	CTGTCATTAGTCACCTGAGT
SEC61B-pegF-OT4	GCCAATGCTTGGTGCCAGCT	GAGGCTGTTTCTGAAGCCTT
SEC61B-pegF-OT5	GGGACATGTGCTTTTGGAGC	AGTGGCTGGAACCTCCTCAG
SEC61B-pegF-OT6	GACATCTTCTTAACCCACCC	TCTGCTCCCAGAACTCTAAA
SEC61B-pegR-OT1	CCATTCATTTTGCTCCTGTG	CAACGCAAGTAATGAGAGTT
SEC61B-pegR-OT2	CCAGGTGAGAGAAACTATGG	CTCTTGGCACCTGGTCCTTC
SEC61B-pegR-OT3	TATAGGACCAACAGGCTCAT	TGCCCCGACAAATCAACAAC
SEC61B-pegR-OT4	AGGGCACCCCCACCTCCCAC	ACCCCGTCAGGCCAGCTTCC
SEC61B-pegR-OT5	TTGCCCTTCAAGTCCAGGCT	GAAGTTCCCTGTAATAAAAG
RUNX1-pegR-OT6	TTGTTCCATGAACCAATGGG	TATGAACTGAAGTTGTGCTT

Supplementary Notes

EGFP reporter

EF-1α promoter

EGFP sequence

mCherry insertion sequence

GGCTCCGGTGCCCGTCAGTGGGCAGAGCGCACATCGCCCACAGTCCCCGAGAAGT
TGGGGGGAGGGGTCGGCAATTGATCCGGTGCCTAGAGAAGGTGGCGCGGGGTAA
ACTGGGAAAGTGATGTCGTGTACTGGCTCCGCCTTTTTCCCGAGGGTGGGGGAGAA
CCGTATATAAGTGCAGTAGTCGCCGTGAACGTTCTTTTTCGCAACGGGTTTGCCGC
CAGAACACAGGTAAGTGCCGTGTGTGGTTCCCGCGGGCCTGGCCTCTTTACGGGTT
ATGGCCCTTGCGTGCCTTGAATTACTTCCACTGGCTGCAGTACGTGATTCTTGATCC
CGAGCTTCGGGTTGGAAGTGGGTGGGAGAGTTCGAGGCCTTGCGCTTAAGGAGCC
CCTTCGCCTCGTGCTTGAGTTGAGGCCTGGCCTGGGCGCTGGGGCCGCCGCGTGC
GAATCTGGTGGCACCTTCGCGCCTGTCTCGCTGCTTTCGATAAGTCTCTAGCCATTT
AAAATTTTTGATGACCTGCTGCGACGCTTTTTTTCTGGCAAGATAGTCTTGTAAATGC
GGGCCAAGATCTGCACACTGGTATTTCGGTTTTTGGGGCCGCGGGCGGCGACGGG
GCCCGTGCGTCCCAGCGCACATGTTCGGCGAGGCGGGGCCTGCGAGCGCGGCCA
CCGAGAATCGGACGGGGGTAGTCTCAAGCTGGCCGGCCTGCTCTGGTGCCTGGCC
TCGCGCCGCCGTGTATCGCCCCGCCCTGGGCGGCAAGGCTGGCCCGGTCGGCAC
CAGTTGCGTGAGCGGAAAGATGGCCGCTTCCCGGCCCTGCTGCAGGGAGCTCAAA
ATGGAGGACGCGGCGCTCGGGAGAGCGGGCGGGTGAGTCACCCACACAAAGGAA
AAGGGCCTTTCCGTCCTCAGCCGTCGCTTCATGTGACTCCACGGAGTACCGGGCG
CCGTCCAGGCACCTCGATTAGTTCTCGAGCTTTTGGAGTACGTCGTCTTTAGGTTGG
GGGGAGGGGTTTTATGCGATGGAGTTTCCCCACACTGAGTGGGTGGAGACTGAAG
TTAGGCCAGCTTGGCACTTGATGTAATTCTCCTTGGAATTTGCCCTTTTTGAGTTTGG
ATCTTGGTTCATTCTCAAGCCTCAGACAGTGGTTCAAAGTTTTTTTCTTCCATTTCAG
GTGTCGTGACGTACGGCCACCATGGTGAGCAAGGGCGAGGAGCTGTTCACCGGGG
TGGTGCCCATCCTGGTCGAGCTGGACGGCGACGTAAACGGCCACAAGTTCAGCGT
GTCCGGCGAGGGCGAGGGCGATGCCACCTACGGCAAGCTGACCCTGAAGTTCATC
TGCACCACCGGCAAGCTGCCCGTGCCCTGGCCCACCCTCGTGACCACCCTGACCT
ACGGCGTGCAGTGCTTCAGCCGCTACCCCGACCACATGAAGCAGCACGACTTCTTC
AAGTCCGCCATGCCCGAAGGCTACGTCCAGGAGCGCACCATCTTCTTCAAGGACGA
CGGCAACTACAAGACCCGCGCCGAGGTGAAGTTCGAGGGCGACACCCTGGTGGGT
GAGCAAGGGCGAGGAGGATAACATGGCCATCATCAAGGAGTTCATGCGCTTCAAG
GTGCACATGGAGGGCTCCGTGAACGGCCACGAGTTCCTCGACATTACCCTGTTATC
CCTACGCAAATAAGAGCTCGAGGGCCGCCCCTACGAGGGCACCCAGACCGCCAAG
CTGAAGGTGACCAAGGGTGGCCCCCTGCCCTTCGCCTGGGACATCCTGTCCCCTC
AGTTCATGTACGGCTCCAAGGCCTACGTGAAGCACCCCGCCGACATCCCCGACTAC
TTGAAGCTGTCCTTCCCCGAGGGCTTCAAGTGGGAGCGCGTGATGAACTTCGAGGA
CGGCGGCGTGGTGACCGTGACCCAGGACTCCTCCCTGCAGGACGGCGAGTTCATC
TACAAGGTGAAGCTGCGCGGCACCAACTTCCCCTCCGACGGCCCCGTAATGCAGA
AGAAGACCATGGGCTGGGAGGCCTCCTCCGAGCGGATGTACCCCGAGGACGGCG
CCCTGAAGGGCGAGATCAAGCAGAGGCTGAAGCTGAAGGACGGCGGCCACTACGA
CGCTGAGGTCAAGACCACCTACAAGGCCAAGAAGCCCGTGCAGCTGCCCGGCGCC

TACAACGTCAACATCAAGTTGGACATCACCTCCCACAACGAGGACTACACCATCGTG
GAACAGTACGAACGCGCCGAGGGCCGCCACTCCACCGGCGGCATGGACGAGCTG
TACAAGAACCGCATCGAGCTGAAGGGCATCGACTTCAAGGAGGACGGCAACATCCT
GGGGCACAAGCTGGAGTACAACTACAACAGCCACAACGTCTATATCATGGCCGACA
AGCAGAAGAACGGCATCAAGGTGAACTTCAAGATCCGCCACAACATCGAGGACGGC
AGCGTGCAGCTCGCCGACCACTACCAGCAGAACACCCCCATCGGCGACGGCCCCG
TGCTGCTGCCCGACAACCACTACCTGAGCACCCAGTCCGCCCTGAGCAAAGACCC
CAACGAGAAGCGCGATCACATGGTCCTGCTGGAGTTCGTGACCGCCGCCGGGATC
ACTCTCGGCATGGACGAGCTGTACAAGTGA

uDAR editor

NLS
Cas9 nuclease
Linker
P2A
Reverse transcriptase
i53(UbvG08(I44A))

gagagccgccaccatgaaacggacagccgacggaagcgagttcgagtcaccaaagaagaagcgga
aagtcgacaagaagtacagcatcggcctggacatcggcaccaactctgtgggctgggccgtgatcaccg
acgagtacaaggtgcccagcaagaaattcaaggtgctgggcaacaccgaccggcacagcatcaagaa
gaacctgatcggagccctgctgttcgacagcggcgaaacagccgaggccacccggctgaagagaacc
gccagaagaagatacaccagacggaagaaccggatctgctatctgcaagagatcttcagcaacgagat
ggccaaggtggacgacagcttcttccacagactggaagagtccttcctggtggaagaggataagaagca
cgagcggcaccccatcttcggcaacatcgtggacgaggtggcctaccacgagaagtaccccaccatcta
ccacctgagaaagaaactggtggacagcaccgacaaggccgacctgcggctgatctatctggccctggc
ccacatgatcaagttccggggccacttcctgatcgagggcgacctgaaccccgacaacagcgacgtgga
caagctgttcatccagctggtgcagacctacaaccagctgttcgaggaaaaccccatcaacgccagcggc
gtggacgccaaggccatcctgtctgccagactgagcaagagcagaaagctggaaaatctgatcgcccag
ctgcccggcgagaagaagaatggcctgttcggaaacctgattgccctgagcctgggcctgacccccaactt
caagagcaacttcgacctggccgaggatgccaaactgcagctgagcaaggacacctacgacgacgac
ctggacaacctgctggcccagatcggcgaccagtacgccgacctgtttctggccgccaagaacctgtccg
acgccatcctgctgagcgacatcctgagagtgaacaccgagatcaccaaggcccccctgagcgcctctat
gatcaagagatacgacgagcaccaccaggacctgaccctgctgaaagctctcgtgcggcagcagctgcc
tgagaagtacaaagagattttcttcgaccagagcaagaacggctacgccggctacattgacggcggagc
cagccaggaagagttctacaagttcatcaagcccatcctggaaaagatggacggcaccgaggaactgct
cgtgaagctgaagagagaggacctgctgcggaagcagcggaccttcgacaacggcagcatcccccac
cagatccacctgggagagctgcacgccattctgcggcggcaggaagatttttacccattcctgaaggacaa
ccgggaaaagatcgagaagatcctgaccttccgcatcccctactacgtgggccctctggccaggggaaac
agcagattcgcctggatgaccagaaagagcgaggaaaccatcaccccctggaacttcgaggaagtggt
ggacaagggcgcttccgcccagagcttcatcgagcggatgaccaacttcgataagaacctgcccaacga
gaaggtgctgcccaagcacagcctgctgtacgagtacttcaccgtgtataacgagctgaccaaagtgaaat
acgtgaccgagggaatgagaaagcccgccttcctgagcggcgagcagaaaaaggccatcgtggacctg
ctgttcaagaccaaccggaaagtgaccgtgaagcagctgaaagaggactacttcaagaaaatcgagtgc
ttcgactccgtggaaatctccggcgtggaagatcggttcaacgcctccctgggcacataccacgatctgctg

aaaattatcaaggacaaggacttcctggacaatgaggaaaacgaggacattctggaagatatcgtgctga
ccctgacactgtttgaggacagagagatgatcgaggaacggctgaaaacctatgcccacctgttcgacga
caaagtgatgaagcagctgaagcggcggagatacaccggctggggcaggctgagccggaagctgatc
aacggcatccgggacaagcagtccggcaagacaatcctggatttcctgaagtccgacggcttcgccaac
agaaacttcatgcagctgatccacgacgacagcctgacctttaaagaggacatccagaaagcccaggtgt
ccggccagggcgatagcctgcacgagcacattgccaatctggccggcagccccgccattaagaagggc
atcctgcagacagtgaaggtggtggacgagctcgtgaaagtgatgggccggcacaagcccgagaacat
cgtgatcgaaatggccagagagaaccagaccacccagaagggacagaagaacagccgcgagagaa
tgaagcggatcgaagagggcatcaaagagctgggcagccagatcctgaaagaacaccccgtggaaaa
cacccagctgcagaacgagaagctgtacctgtactacctgcagaatgggcgggatatgtacgtggaccag
gaactggacatcaaccggctgtccgactacgatgtggacgctatcgtgcctcagagctttctgaaggacga
ctccatcgacaacaaggtgctgaccagaagcgacaagaaccggggcaagagcgacaacgtgccctcc
gaagaggtcgtgaagaagatgaagaactactggcggcagctgctgaacgccaagctgattacccagag
aaagttcgacaatctgaccaaggccgagagaggcggcctgagcgaactggataaggccggcttcatca
agagacagctggtggaaacccggcagatcacaaagcacgtggcacagatcctggactcccggatgaac
actaagtacgacgagaatgacaagctgatccgggaagtgaaagtgatcaccctgaagtccaagctggtgt
ccgatttccggaaggatttccagttttacaaagtgcgcgagatcaacaactaccaccacgcccacgacgcc
tacctgaacgccgtcgtgggaaccgccctgatcaaaaagtaccctaagctggaaagcgagttcgtgtacg
gcgactacaaggtgtacgacgtgcggaagatgatcgccaagagcgagcaggaaatcggcaaggctac
cgccaagtacttcttctacagcaacatcatgaactttttcaagaccgagattaccctggccaacggcgagat
ccggaagcggcctctgatcgagacaaacggcgaaaccggggagatcgtgtgggataagggccgggatt
ttgccaccgtgcggaaagtgctgagcatgccccaagtgaatatcgtgaaaaagaccgaggtgcagacag
gcggcttcagcaaagagtctatcctgcccaagaggaacagcgataagctgatcgccagaaagaaggac
tgggaccctaagaagtacggcggcttcgacagccccaccgtggcctattctgtgctggtggtggccaaagt
ggaaaagggcaagtccaagaaactgaagagtgtgaaagagctgctggggatcaccatcatggaaaga
agcagcttcgagaagaatcccatcgactttctggaagccaagggctacaaagaagtgaaaaaggacctg
atcatcaagctgcctaagtactccctgttcgagctggaaaacggccggaagagaatgctggcctctgccgg
cgaactgcagaagggaaacgaactggccctgccctccaaatatgtgaacttcctgtacctggccagccact
atgagaagctgaagggctcccccgaggataatgagcagaaacagctgtttgtggaacagcacaagcact
acctggacgagatcatcgagcagatcagcgagttctccaagagagtgatcctggccgacgctaatctgga
caaagtgctgtccgcctacaacaagcaccgggataagcccatcagagagcaggccgagaatatcatcc
acctgtttaccctgaccaatctgggagcccctgccgccttcaagtactttgacaccaccatcgaccggaaga
ggtacaccagcaccaaagaggtgctggacgccaccctgatccaccagagcatcaccggcctgtacgag
acacggatcgacctgtctcagctgggaggtgactccggcggaagctctggtggcagcaagcggaccgcc
gacggctctgaattcgagagccctaagaagaaaagaaaggtgagcggaggctctagcggcggaagca
ccctgaacattgaagacgagtatagactgcatgaaacaagcaaggaacccgacgtgtccctgggctcca
cctggctgtccgactttccccaggcctgggccgagacaggaggaatgggcctggccgtgcggcaggcac
ccctgatcatccctctgaaggccacctctacacccgtgagcatcaagcagtaccctatgtctcaggaggcc
agactgggcatcaagcctcacatccagaggctgctggaccagggcatcctggtgccatgccagagcccct
ggaacacaccactgctgcccgtgaagaagccaggcaccaatgactatagacccgtgcaggatctgaga
gaggtgaacaagagggtggaggatatccaccccaccgtgcccaacccttacaatctgctgtccggcctgc
cccccttctcaccagtggtatacagtgctggacctgaaggatgccttcttttgtctgagactgcaccctaccagc
cagccactgttcgcctttgagtggagggaccctgagatgggcatctctggccagctgacctggacacgcct
gcctcagggcttcaagaatagcccaacactgtttaacgaggccctgcaccgcgacctggcagatttccgga

tccagcacccagatctgatcctgctgcagtacgtggacgatctgctgctggccgccaccagcgagctggatt
gccagcagggaacacgcgccctgctgcagaccctgggaaacctgggatatagggcatccgccaagaa
ggcccagatctgtcagaagcaggtgaagtacctgggctatctgctgaaggagggccagagatggctgac
agaggccaggaaggagacagtgatgggccagccaacacccaagaccccaagacagctgagggagtt
cctgggcaaagcaggattttgcaggctgttcatcccaggattcgcagagatggcagcacctctgtacccact
gaccaagccgggcaccctgtttaattggggccctgaccagcagaaggcctatcaggagatcaagcaggc
cctgctgacagcaccagccctgggcctgccagacctgaccaagcctttcgagctgtttgtggatgagaagc
agggctacgccaagggcgtgctgacccagaagctgggaccatggagacggcccgtggcctatctgtcca
agaagctggacccagtggcagcaggatggccaccatgcctgaggatggtggcagcaatcgccgtgctga
caaaggatgccggcaagctgaccatgggacagccactggtcatcctggcaccacacgcagtggaggcc
ctggtgaagcagcctccagatcgctggctgtctaacgcccggatgacacactaccaggccctgctgctgga
caccgatcgcgtgcagtttggccctgtggtggccctgaatccagccaccctgctgcctctgccagaggagg
gcctgcagcacaactgtctggacatcctggcagaggcacacggaacaaggccagacctgaccgatcag
cccctgcctgacgccgatcacacatggtataccgatggaagctccctgctgcaggagggccagaggaag
gcaggagcagcagtgaccacagagacagaagtgatctgggccaaggccctgccagcaggcacatccg
cccagcgggccgagctgatcgccctgacccaggccctgaagatggccgagggcaagaagctgaacgt
gtacacagactccagatatgccttcgccaccgcacacatccacggagagatctacaggcgccggggctg
gctgacctctgagggcaaggagatcaagaacaaggatgagatcctggccctgctgaaggccctgtttctgc
ccaagcggctgagcatcatccactgtcctggacaccagaagggacactccgccgaggcaaggggcaat
cggatggccgaccaggccgccagaaaggctgctattactgaaactcccgacacttccactctgctgattga
aaactcctcccttctggcggctcaaaaagaaccgccgacggcagcgaattcgagtctcccaagaagaa
gaggaaagtcggctctggccctgccgctaagagagtgaagctggacggatccggcgcaacaaacttctct
ctgctgaaacaagccggagatgtcgaagagaatcctggaccgatgcatatgaaacggacagccgacgg
aagcgagttcgagtcaccaaagaagaagcggaaagtcgccgccagtttaaacggcgcgccattaattaa
ggatccaatgttgattttcgtgaaaacccttaccgggaaaaccatcaccctcgaggttgaaccctcggatac
gatagaaaatgtaaaggccaagatccaggataaggaaggaattcctcctgatcagcagagactggccttt
gctggcaaatcgctggaagatggacgtactttgtctgactacaatattctaaaggactctaaacttcatcctct
gttgagacttcgttaa

Original blots for Gel results are shown below.

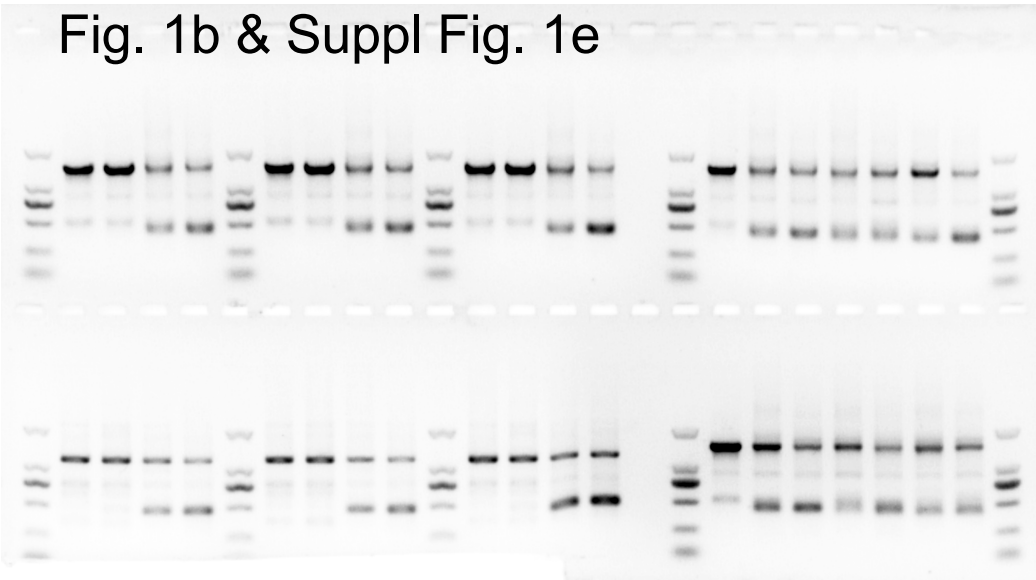

Fig. 1b & Suppl Fig. 1e

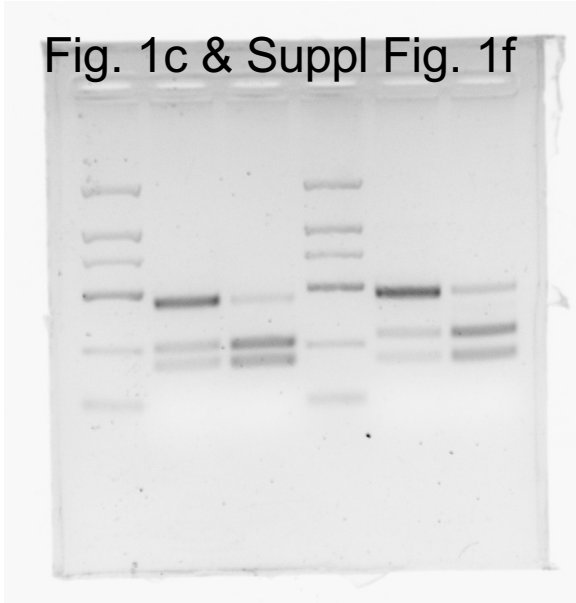

Fig. 1c & Suppl Fig. 1f

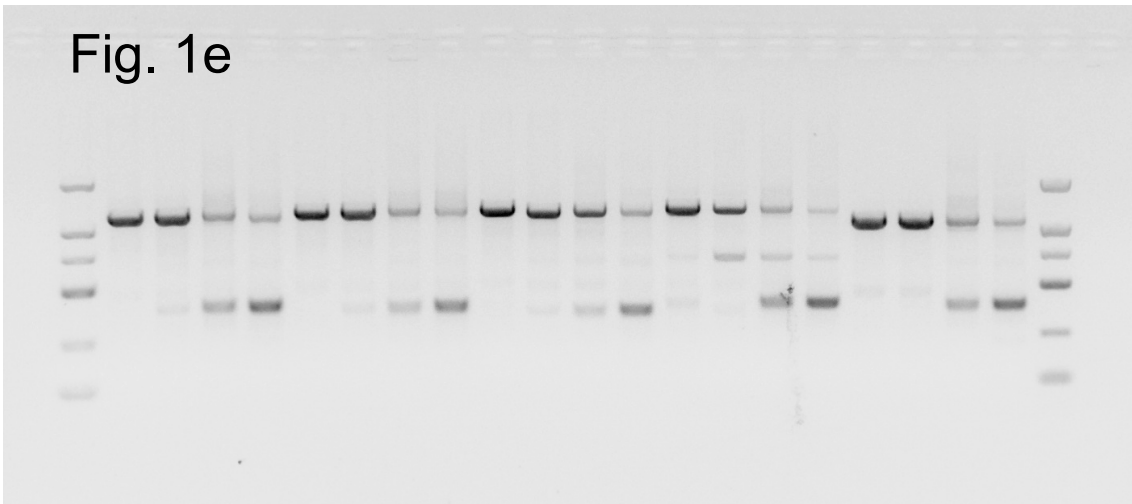

Fig. 1e

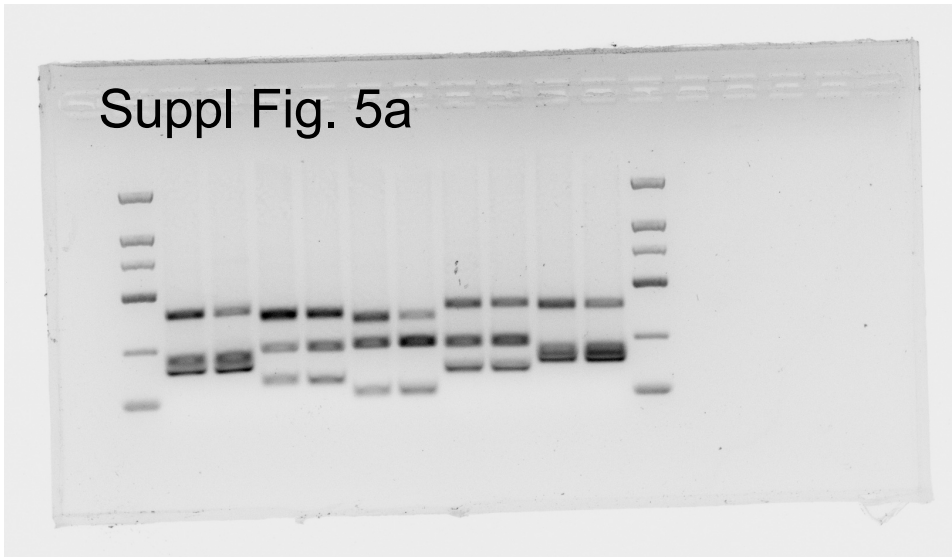

Suppl Fig. 5a

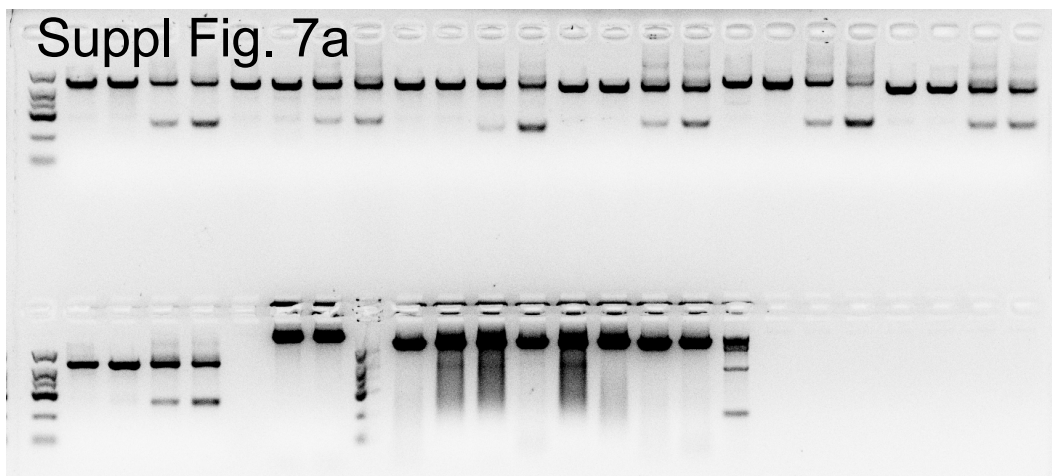

Suppl Fig. 7a

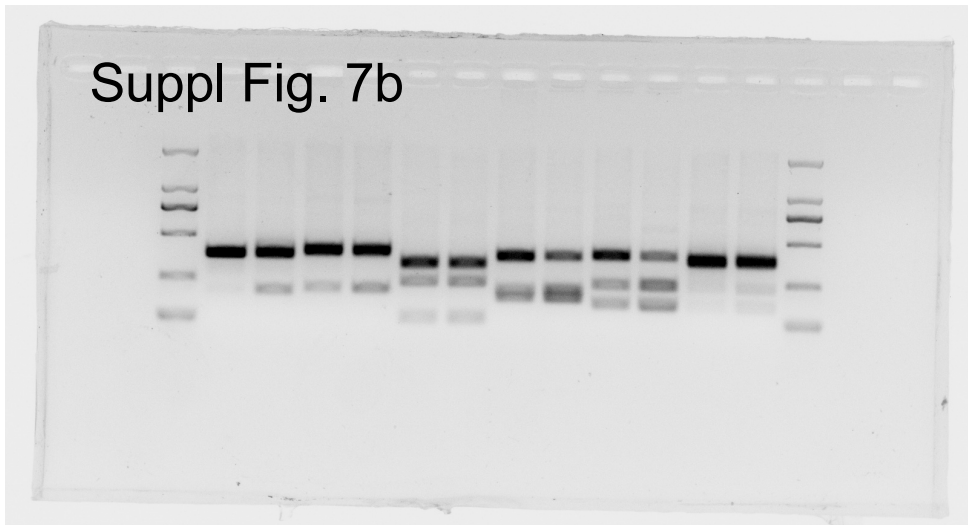

Suppl Fig. 7b

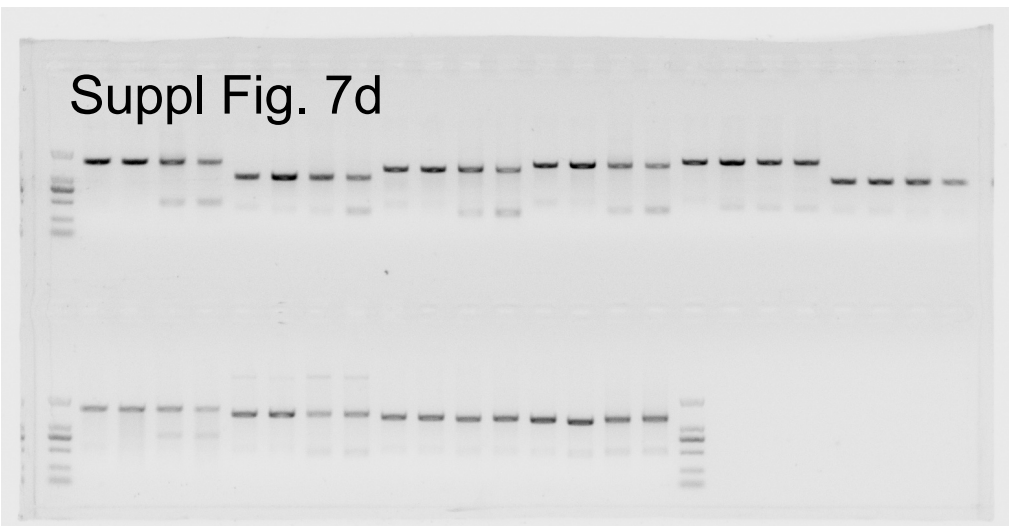

Suppl Fig. 7d

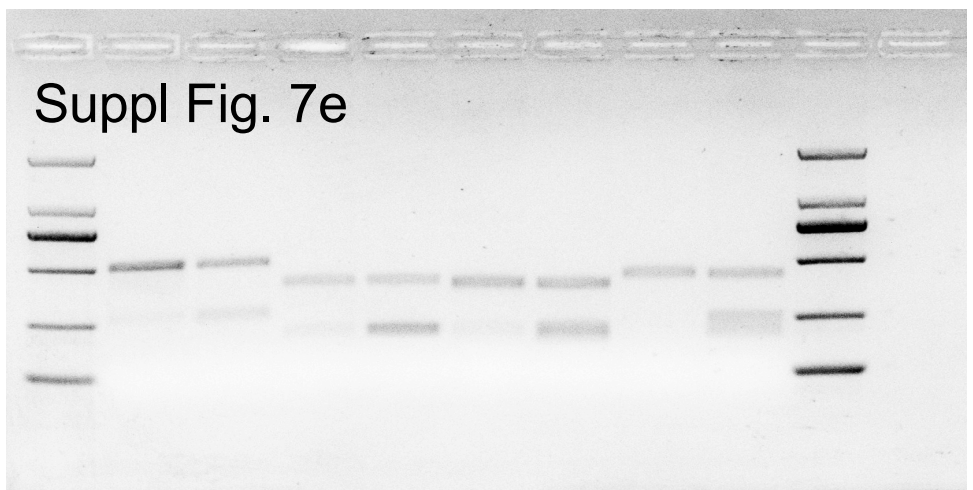

Suppl Fig. 7e

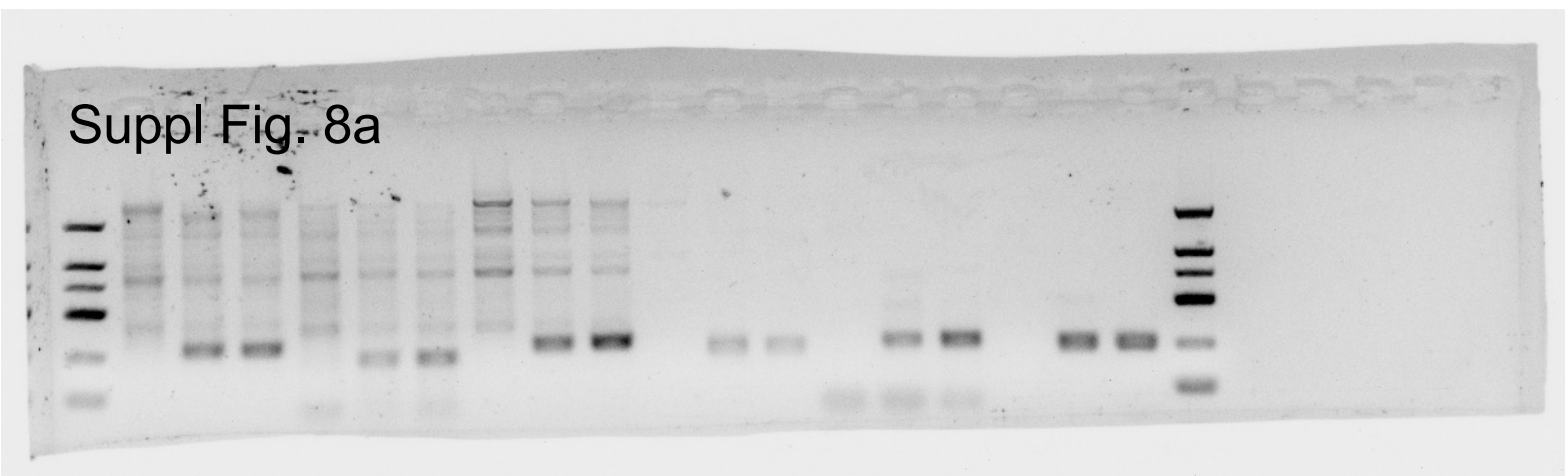

Suppl Fig. 8a

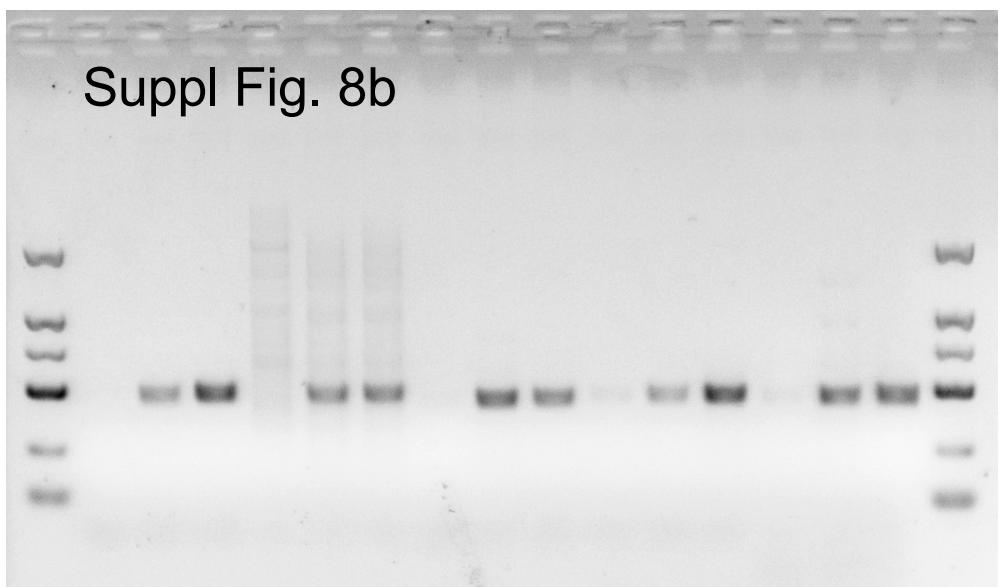

Suppl Fig. 8b

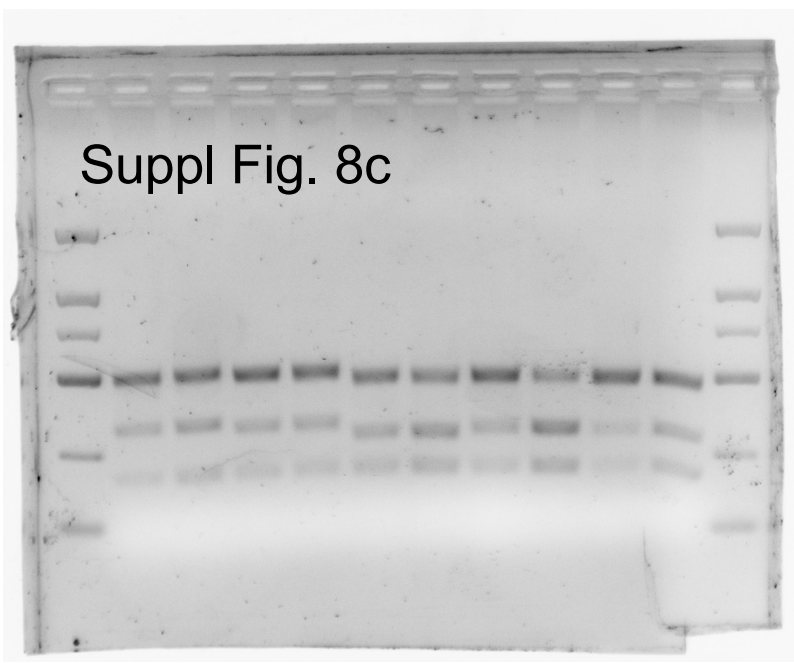

Suppl Fig. 8c

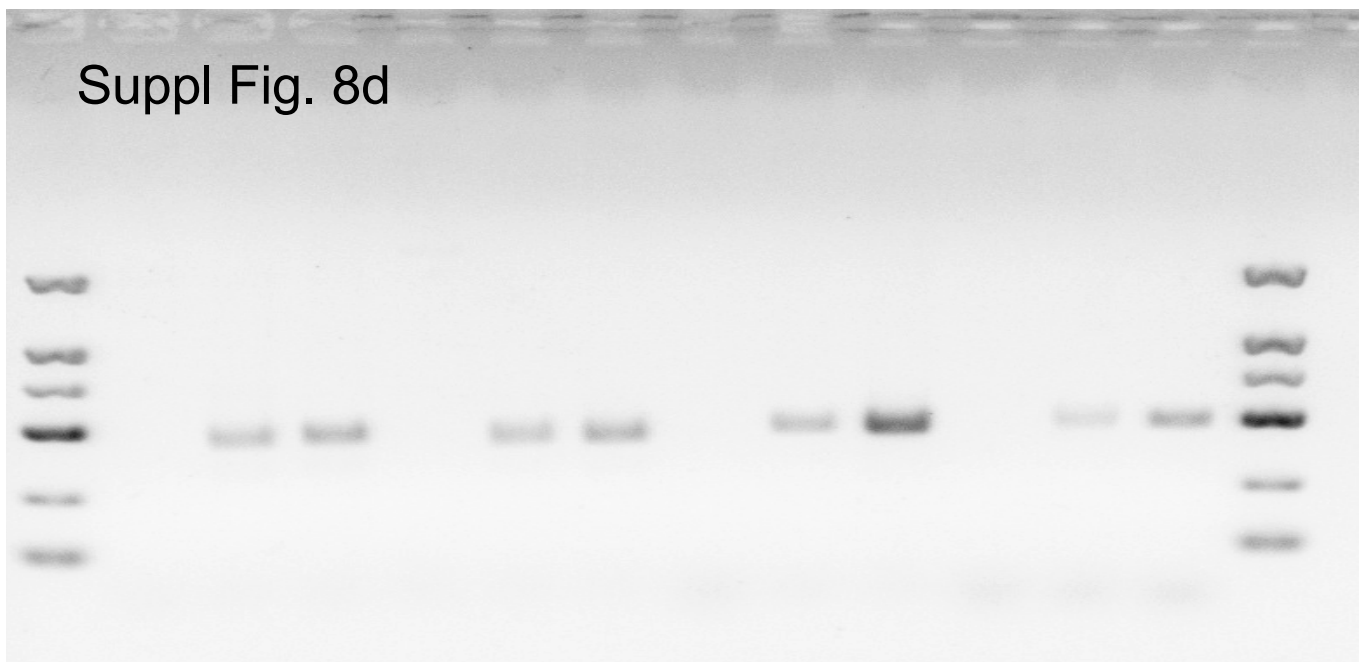
Suppl Fig. 8d

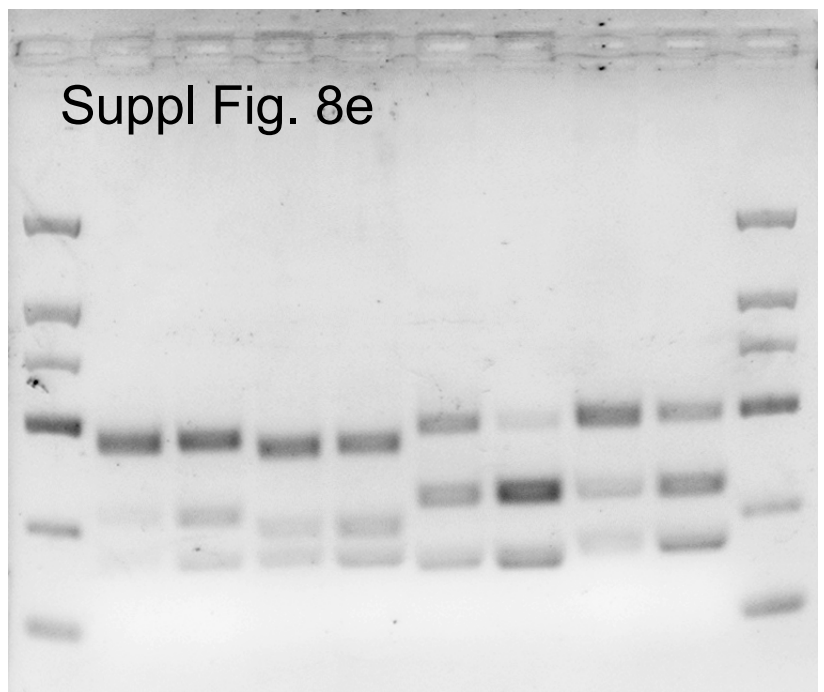
Suppl Fig. 8e

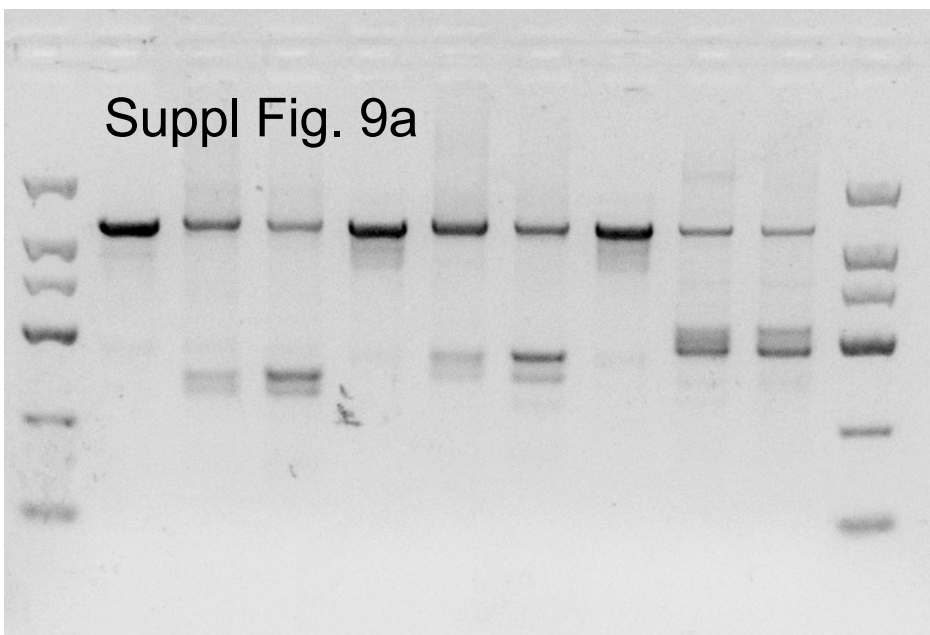
Suppl Fig. 9a

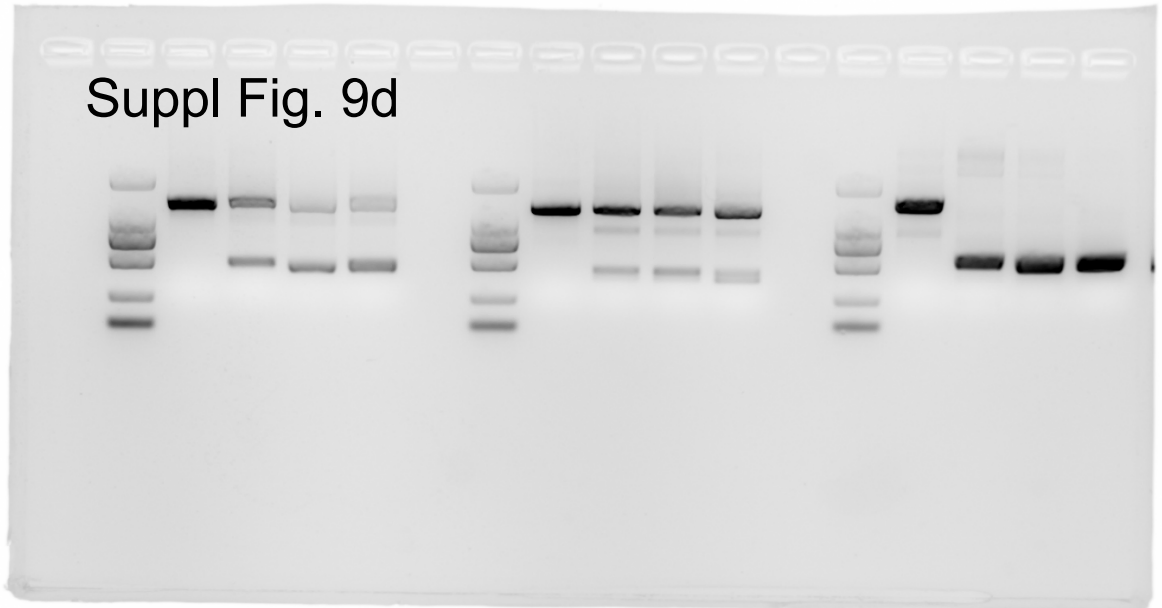
Suppl Fig. 9d

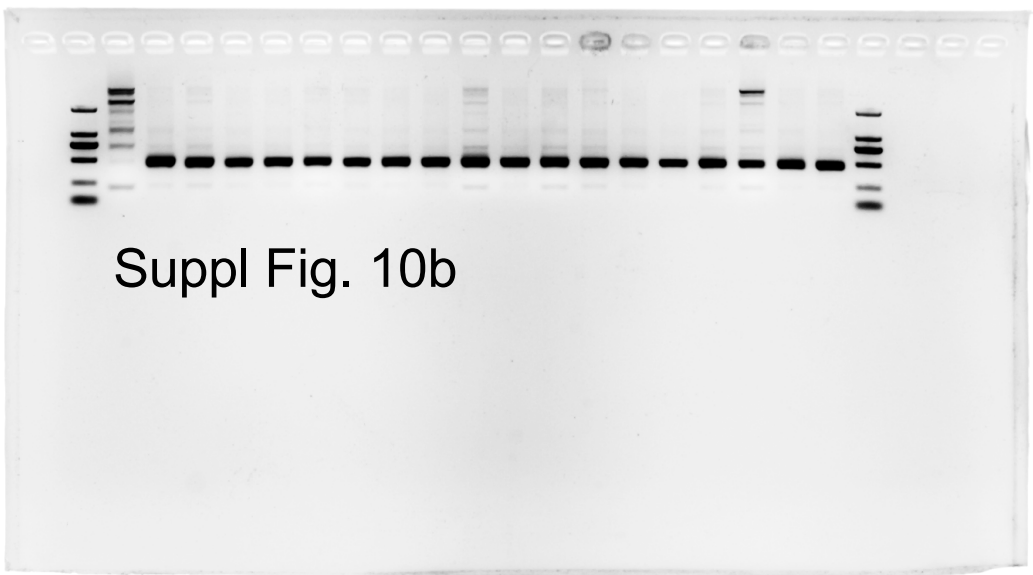
Suppl Fig. 10b

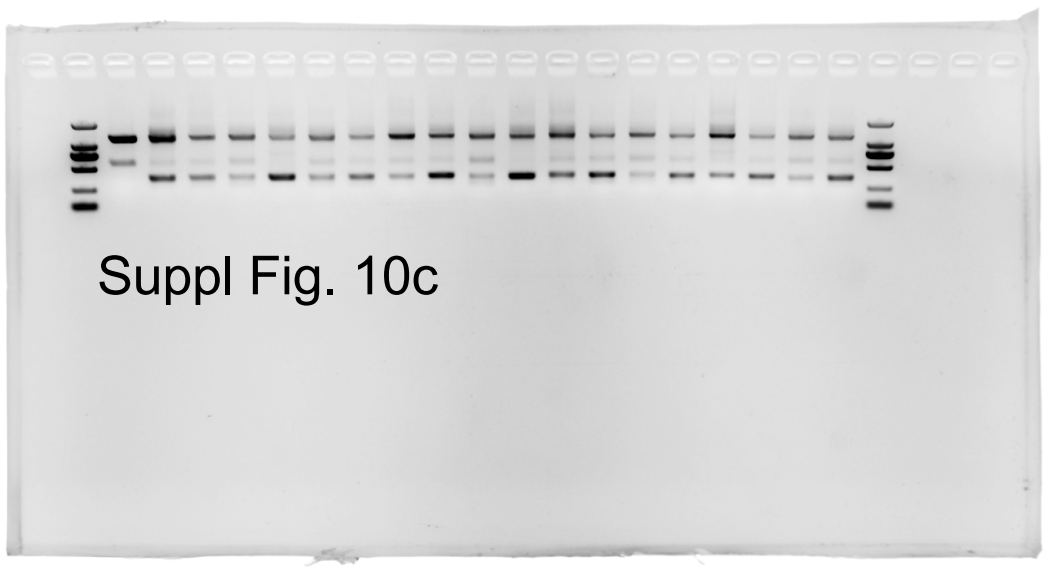
Suppl Fig. 10c

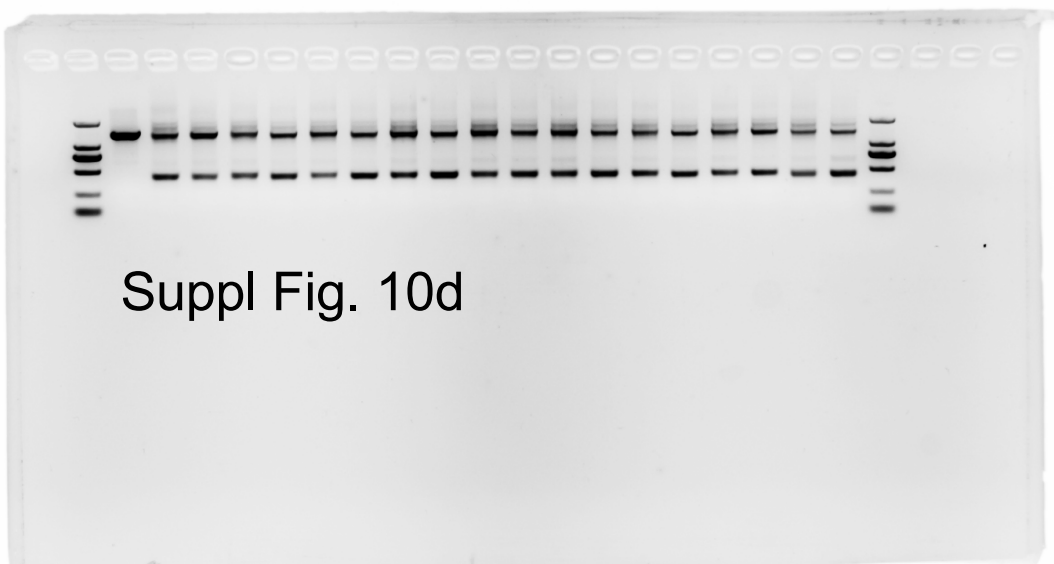
Suppl Fig. 10d

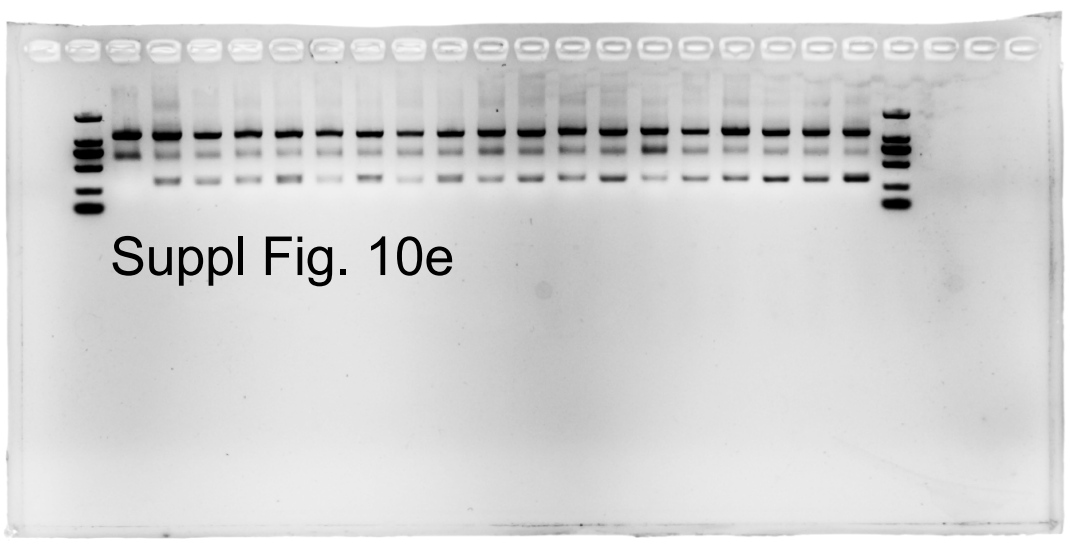
Suppl Fig. 10e

Suppl Fig. 11a

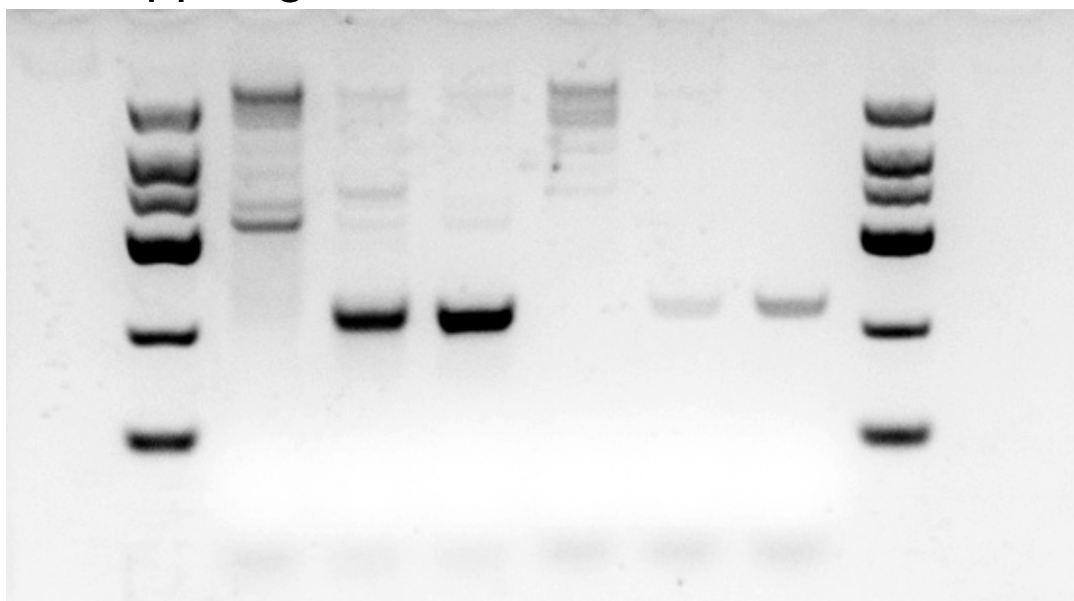

Suppl Fig. 11c

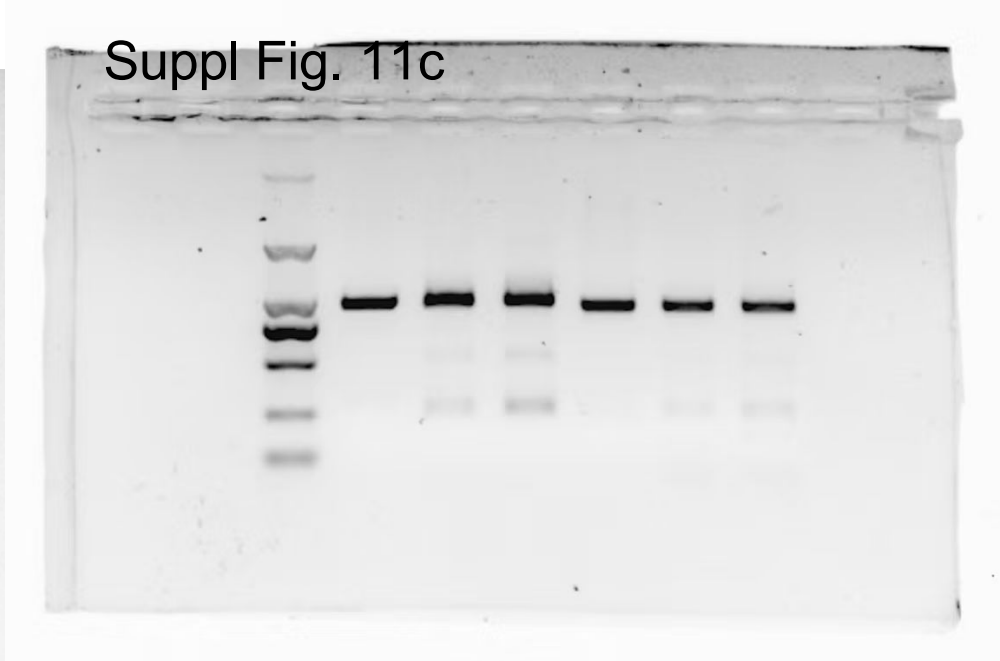

Suppl Fig. 14a

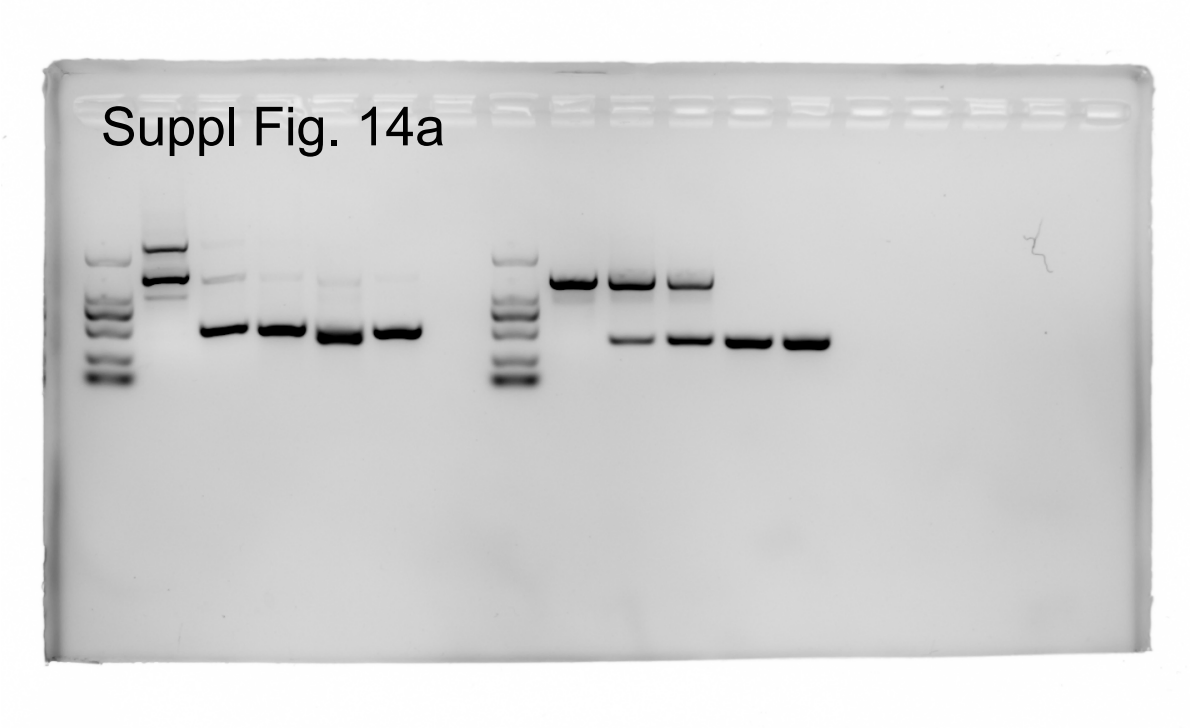

Supplement: Supplementary file 1 [file 41421_2026_923_MOESM1_ESM.pdf]
